# Baricitinib in patients admitted to hospital with COVID-19 (RECOVERY): a randomised, controlled, open-label, platform trial and updated meta-analysis

**DOI:** 10.1016/S0140-6736(22)01109-6

**Published:** 2022-07-30

**Authors:** RECOVERY Collaborative Group, Obbina Abani, Ali Abbas, Fatima Abbas, Joshua Abbas, Kasim Abbas, Mustafa Abbas, Sadia Abbasi, Hakam Abbass, Alfie Abbott, Alison Abbott, Nabeel Abdallah, Ammar Abdelaziz, Ashraf Abdelaziz, Mohamed Abdelfattah, Bushra Abdelqader, Audrey Abdul, Basir Abdul, Siddiqui Abdul, Althaf Abdul Rasheed, Ajibode Abdulakeem, Rezan Abdul-Kadir, Abdullah Abdullah, Abdulfatahi Abdulmumeen, Rasheed Abdul-Raheem, Niyaz Abdulshukkoor, Kula Abdusamad, Yazeed Abed El Khaleq, Mai Abedalla, Abeer Ul Amna, Lynn Abel, Katrina Abernethy, Movin Abeywickrema, Chandra Abhinaya, Affyarsyah Abidin, Adebanke Aboaba, Abigail Aboagye-Odei, Heba Aboelela, Hani Abo-Leyah, Karim Abouelela, Ahmed Abou-Haggar, Mahmoud Abouibrahim, Ahmed Abousamra, Mona Abouzaid, Miriam Abraham, Tizzy Abraham, Abraheem Abraheem, Judith Abrams, Rebecca Abrams, Hyacinth-John Abu, Ahmed Abu-Arafeh, Syed Mohamed Abubacker, Akata Abung, Yousuf Abusamra, Yaa Aceampong, Amaka Achara, Devikumar Acharya, Prince Acheampong, Sarah Acheampong, Janet Acheson, Shiella Achieng, Andres Acosta, Rebecca Acquah, Catherine Acton, Jacqueline Adabie-Ankrah, Paul Adair, Fiona Adam, Matthew Adam, Huzaifa Adamali, Marta Adamczyk, Carol Adams, Charlotte Adams, Daniel Adams, Kate Adams, Laura Adams, Nikkita Adams, Richard Adams, Tim Adams, Laura Adamu-Ikeme, Krishma Adatia, Kirsty Adcock, Afua Addo, Oluwatobi Adeagbo, Ade Adebiyi, Ogunlana Adedeji, Yewande Adegeye, Ken Adegoke, Vicki Adell, Sherna Adenwalla, Femi W Adeoye, Oluwasegun A Adesemoye, Emmanuel O Adewunmi, Adedamola Adeyanju, Joyce Adeyemi, Tenifayo Adeyemo, Binay Adhikari, Rina Adhikary, Adhikarla Aditya, Gabrielle Adkins, Adnan Adnan, John Aeron-Thomas, Debbie Affleck, Carmel Afnan, Muhammad Afridi, Zainab A Aftab, Meenakshi Agarwal, Rachel Agbeko, Chris Agbo, Sunil Aggarwal, Arameh Aghababaie, Laura Aguilar Jimenez, Shafana Ahamed Sadiq, Mohamed H Ahammed Nazeer, Mohammad Ahmad, Syed Ahmad, Afshan Ahmed, Ashar Ahmed, Asim Ahmed, Basheer A R Ahmed, Bilal Ahmed, Forizuddin Ahmed, Hamze Ahmed, Hanad Ahmed, Irshad Ahmed, Khaled Ahmed, Khalil Ahmed, Liban Ahmed, Mahin Ahmed, Maria C Ahmed, Muhammad S Ahmed, Naseer Ahmed, Nausheen Ahmed, Osama Ahmed, Rajia A Ahmed, Rawya Ahmed, Rizwan Ahmed, Saif Ahmed, Sammiya Ahmed, Sana G Ahmed, Syed Ahmed, Syed H Ahmed, Roa Ahmed Ali, Bilal Ahmed Mohamud, Sana Ahmed, Sana Ahmer, Augustine Ahonia, Christine Aiken, Dhiraj Ail, Mark Ainsworth, Myriam Aissa, Lindianne Aitken, Bini Ajay, Abdulakeem Ajibode, Ayesha Ajmi, Nasim Akhtar, Nauman Akhtar, Suha Akili, Bolutito Akinbiyi, Oludoyinsola Akindolie, Yinka Akinfenwa, Olugbenga Akinkugbe, Ibrahim Akinpelu, Mohammad Akram, Olugbenro Aktinade, Uzayr Akudi, Ahmad S A R Al Aaraj, Asma Al Balushi, Majd Al Dakhola, Aladdin Al Swaifi, Eslam Al-Abadi, Adegoke Alabi, Narendra Aladangady, Mohamed Alafifi, Ayaz Alam, Sajid Alam, Abbas Al-Asadi, Kyriaki Alatzoglou, Paul Albert, Lorraine Albon, Angela Alcala, Gemma Alcorn, Stephen Alcorn, Aggie Aldana, David Alderdice, Abdullah Aldesouki, Rayan Aldouri, Jonathan Aldridge, Nicolas Aldridge, Ram M Ale, Ana Alegria, Alison Alexander, Courtney Alexander, John Alexander, Peter D G Alexander, Julyan Al-fori, Laith Alghazawi, Osama Alhabsha, Bahij Al-Hakim, Rafil Alhameed, Mohammed Al-Hayali, Shams Al-Hity, Ali Ali, Amanda Ali, Asad Ali, Fawzia R Ali, Jawad Ali, Mariam Ali, Mohamed Ali, Mohammad Ali, Muhammad S Ali, Nayab Ali, Oudai Ali, Ramla Ali, Sakina Ali, Syed Ali, Emilio Aliberti, Jade Alin, Abid Alina, Analyn Alipustain, Fine Aliyuda, Katrin Alizadeh, Maithem Al-Jibury, Saba Al-Juboori, Majid Al-Khalil, Abdullah Alkhudhayri, Moutaz Alkhusheh, Fiona Allan, Nataliya Allan, Alison Allanson, Robert Allcock, Eireann Allen, Jade Allen, Kerry Allen, Louise Allen, Poppy Allen, Rebecca Allen, Sam Allen, Sharon Allen, Simon Allen, Tania Allen, Abiola Alli, Kathryn Allison, Bethan Allman, Helen K Allsop, Lynne Allsop, David Allsup, Amal F T Almahroos, Hassan Al-Moasseb, Magda Al-Obaidi, Lina Alomari, Akram Al-Rabahi, Bahar Al-Ramadhani, Zayneb Al-Saadi, Riyam Al-Sammarraie, Inji Alshaer, Rustam Al-Shahi Salman, Warkaq Al-Shamkhani, Fadia Alsheikh, Bashar Al-Sheklly, Sara Altaf, Amanda Alty, Mary Alvarez, Maria Alvarez Corral, Edward Alveyn, Maysaa Alzetani, Susan Amamou, Noor Amar, Sakkarai Ambalavanan, Robert Ambrogetti, Chris Ambrose, Amir Ameen, Katharine Ames, Maria R Amezaga, Allison Amin, Amina Amin, Kanish Amin, Syed Amin, Tara Amin, Badshah Amit, Amjad Amjad, Neelma Amjad, Victoria Amosun, Muhammad Amsal, Khaled Amsha, Noelia Amutio Martin, Pugh Amy, Ann Anada, Atul Anand, Samantha Anandappa, Satya D Anantapatnaikuni, Ellen Anderson, Julie Anderson, Laura Anderson, Marina Anderson, Michelle Anderson, Nicola Anderson, Rachel Anderson, Rebecca Anderson, Rory Anderson, Samantha Anderson, Wendy Anderson, Prematie Andreou, Angela Andrews, Antonette Andrews, Jill Andrews, Joyann Andrews, Kanayochukwu Aneke, Andrew Ang, Wan Wei Ang, Tammy Angel, Aramburo Angela, Paola Angelini, Lazarus Anguvaa, Oleg Anichtchik, Millicent Anim-Somuah, Krishnan Aniruddhan, Jessica Annett, Louise Anning, Patrick J Anstey, Rebekah Anstey, Alpha Anthony, Aaron Anthony-Pillai, Philip Antill, Zhelyazkova Antonina, Varghese Anu, Muhammad Anwar, Elena Apetri, Aristeidis Apostolopoulos, Sarah Appleby, Diane Appleyard, Maia F Aquino, Bianca Araba, Samuel Aransiola, Mariana Araujo, Ann Archer, Denise Archer, Samuel Archer, Simon Archer, Daniele Arcoria, Christian Ardley, Ana-Maria Arias, Oluwatoyin Aribike, Ryoki Arimoto, Charlotte Arkley, Charlotte Armah, Ilianna Armata, Jennifer Armistead, Adam Armitage, Ceri Armstrong, Maureen Armstrong, Sonia Armstrong, Wendy Armstrong, Philippa Armtrong, Heike Arndt, Clare Arnison-Newgass, David Arnold, Rachael Arnold, Andrew Arnott, Dhawal Arora, Kavan Arora, Pardeep Arora, Rishi Arora, Arslam Arter, Armena Arumaithurai, Ayush Arya, Rita Arya, Denisa Asandei, Adeeba Asghar, Malik Asghar, Arif Ashab, Catherine Ashbrook-Raby, Helen Ashby, Jan Ashcroft, John Ashcroft, Samuel Ashcroft, Georgia Asher, Zakariya Ashfak, Ayesha Ashfaq, Abdul Ashish, Dawn Ashley, Sally Ashman-Flavell, Sundar Ashok, Abd-El-Aziz Ashour, Muhammad Z Ashraf, Saima Ashraf, Mohammad B Ashraq, Deborah Ashton, Susan Ashton, Andrew Ashworth, Fiona J Ashworth, Rebecca Ashworth, Arshia Aslam, I Aslam, Suhail Aslam, Laura Aslett, Harshini Asogan, Atif Asrar, Omar Assaf, Raine Astin-Chamberlain, Deborah Athorne, Billie Atkins, Christopher Atkins, Stacey Atkins, John Atkinson, Vicki Atkinson, Abolaji Atomode, Brygitta Atraskiewicz, Abdul A Attia, Eva Attubato, Marie Attwood, Paula Aubrey, Zoltan Auer, Avinash Aujayeb, Aye C T Aung, Hnin Aung, Hnin W W Aung, Ko K Aung, Kyaw T Aung, Nwe Aung, Yin Aung, Zaw M Aung, Emily Austin, Karen Austin, Abdusshakur Auwal, Malcolm Avari, Miriam Avery, Nicholas Aveyard, Joanne Avis, Georgina Aviss, Cristina Avram, Paula Avram, Abuzar Awadelkareem, Gabriel Awadzi, Mahmoud Awaly, Atia Awan, Aszad Aya, Eman Ayaz, Amanda Ayers, Jawwad Azam, Ahmed Azeem, Mohammed Azharuddin, Ammar Aziz, Ghazala Aziz, Imran Aziz, N Aziz, Ali Azkoul, Ashaari Azman Shah, Giada Azzopardi, Hocine Azzoug, Fiyinfoluwa Babatunde, Melvin Babi, Babiker Babiker, Gayna Babington, Matthew Babirecki, Marta Babores, Adetona O Babs-Osibodu, Sammy Bacciarelli, Roudi Bachar, Michel-Elie Bachour, Gina Bacon, Jenny Bacon, Bibi Badal, Maryuma Bader, Gurpreet R Badhan, Shreya Badhrinarayanan, Joseph P Bae, Alice Baggaley, Amy Baggott, Graham Bagley, Dinesh Bagmane, Lynsey Bagshaw, Kasra Bahadori, Angela Bailey, James Bailey, Katie Bailey, Lindsey Bailey, Liz Bailey, Lucy Bailey, Maria A Bailey, Morgan Bailey, Pippa Bailey, Sarah Bailey, Hamish Baillie, J Kenneth Baillie, Jennifer Bain, Vikram Bains, David Baird, Eilidh Baird, Kevin Baird, Susan Baird, Tracy Baird, Yolanda Baird, Aiysha Bajandouh, Danielle C Baker, Elizabeth Baker, Evelyn Baker, Johanne Baker, Josephine Baker, Kenneth Baker, Mark Baker, Rebecca Baker, Terri-Anne Baker, Victoria Baker, Hugh Bakere, Nawar Bakerly, Michelle Baker-Moffatt, Ameet Bakhai, Nauman Bakhtiar, Panos Bakoulas, Dhanalakshmi Bakthavatsalam, Niranjan Balachandran, Andrea Balan, Prasath Balasingam, Theodosios Balaskas, Madhu Balasubramaniam, Nicola Balatoni, Alison Balcombe, Alexander Baldwin, Ashley Baldwin, Caron Baldwin, Danielle Baldwin, Fiona Baldwin, Rebekah Baldwin-Jones, Nichola Bale, James Balfour, Matthew Ball, Robert Ball, K Ballard, Ismael Balluz, Craig Balmforth, Emese Balogh, Abeir Baltmr, Amir Baluwala, Gabby Bambridge, Alasdair Bamford, Amy Bamford, Peter Bamford, Adefunke Bamgboye, Elizabeth Bancroft, Hollie Bancroft, Joyce Banda, Krishna Bandaru, Srini Bandi, Nageswar Bandla, Somaditya Bandyopadhyam, Amit Banerjee, Ritwik Banerjee, Omar Bani-Saad, Harrison Banks, Luke Banks, Paul Banks, Claudia Bann, Helen Bannister, Oliver Bannister, Laura Banton, Tran Bao, Mariamma Baptist, Tanya Baqai, Ananya M Baral, Desislava Baramova, Russel Barber, Emma Barbon, Monica Barbosa, Jamie Barbour, Alexander Barclay, Amanda Barclay, Claire Barclay, George Bardsley, Stephanie Bareford, Shahedal Bari, Morris Barimbing, Amy Barker, Debbie Barker, Erandi Barker, Helen Barker, Joseph Barker, Leon Barker, Lesley Barker, Oliver Barker, Kerry Barker-Williams, Sinha Barkha, Juliana Barla, Gavin Barlow, Richard Barlow, Valerie Barlow, James Barnacle, Alex Barnard, Debi Barnes, Nicky Barnes, Rory Barnes, Theresa Barnes, Calum Barnetson, Amy Barnett, Ashton Barnett-Vanes, William Barnsley, Andrew Barr, David Barr, James Barr, Charlotte Barr, Nina Barratt, Shaney Barratt, Manuella Barrera, Amy Barrett, Fiona Barrett, Jessica Barrett, Sue Barrett, Esther Barrow, Jazz Bartholomew, Claire Bartlett, Georgina Bartlett, James Bartlett, Louise Bartlett, Shauna Bartley, Sandra Bartolmeu-Pires, Anna Barton, Greg Barton, Jill Barton, Lorna Barton, Rachael Barton, Rosaleen Baruah, Sonia Baryschpolec, Hannah Bashir, Archana Bashyal, Betsy Basker, Buddha Basnyat, Ayten Basoglu, Alexander Basran, John Bassett, G Bassett, Chris Bassford, Bengisu Bassoy, Victoria Bastion, Anup Bastola, Anupam Basumatary, Precious Basvi, Vipula R Bataduwaarachchi, Tristan Bate, Harry J Bateman, Kathryn Bateman, Vhairi Bateman, Eleanor Bates, Hayley Bates, Michelle Bates, Simon Bates, Sally Batham, Ana Batista, Amit Batla, Dushyant Batra, Harry Batty, Thomas Batty, Alice Batty, Miranda Baum, Rachel Baumber, Carina Bautista, Fareha Bawa, Tanveer Bawa, Fatima S Bawani, Simon Bax, Matt Baxter, Nicola Baxter, Zachary Baxter, Hannah Bayes, Lee-Ann Bayo, Farid Bazari, Rohit Bazaz, Ahmad Bazli, Laura Beacham, Wendy Beadles, Kirsten Beadon, Philip Beak, Andy Beale, Kathy Beard, Jack Bearpark, Alanna Beasley, Sandee Beattie, Karen Beaumont, Dawn Beaumont-Jewell, Theresa Beaver, Sarah Beavis, Christy Beazley, Sarah Beck, Virginia Beckett, Rosie Beckitt, Sarah Beckley, Heidi Beddall, Seonaid Beddows, Deborah Beeby, Sophia Beeby, Gail Beech, Michelle Beecroft, Nigel Beer, Sally Beer, Jane Beety, Gabriela Bega, Alison Begg, Susan Begg, Sara Beghini, Ayesha Begum, Salman Begum, Selina Begum, Teresa Behan, Roya Behrouzi, Jon Beishon, Claire Beith, James Belcher, Holly Belfield, Katherine Belfield, Ajay Belgaumkar, Dina Bell, Gareth Bell, Gillian Bell, Jessica Bell, Lauren Bell, Louise Bell, Nicholas Bell, Pippa Bell, Stephanie Bell, Jennifer Bellamu, Mary Bellamy, Tammy Bellamy, Arianna Bellini, Amanda Bellis, Fionn Bellis, Lesley Bendall, Naveena Benesh, Nicola Benetti, Leonie Benham, Guy Benison-Horner, Sarah Benkenstein, Tracey Benn, Ann Bennett, Caroline Bennett, Denise Bennett, Gillian Bennett, Kaytie Bennett, Kristopher Bennett, Lorraine Bennett, Miriam R Bennett, Sara Bennett, Karen Bennion, Gautham Benoy, Vivienne Benson, Andrew Bentley, James Bentley, Ian Benton, Eva Beranova, Matthew Beresford, Colin Bergin, Malin Bergstrom, Jolanta Bernatoniene, Thomas Berriman, Zoe Berry, Frida Best, Kimberley Best, Ans-Mari Bester, Yvonne Beuvink, Emily Bevan, Sarah Bevins, Tom Bewick, Andrew Bexley, Sonay Beyatli, Fenella Beynon, Arjun Bhadi, Sanjay Bhagani, Shiv Bhakta, Rekha Bhalla, Khushpreet Bhandal, Kulbinder Bhandal, Ashwin Bhandari, Lila N Bhandari, Sangam Bhandari, Jennifer Bhanich Supapol, Aashutosh Bhanot, Ravina Bhanot, Swati Bhasin, Abdul Bhat, Prashanth Bhat, Rahul Bhatnagar, Karan Bhatt, Janki Bhayani, Deepika Bhojwani, Parminder Bhuie, Salimuzzaman Bhuiyan, Anna Bibby, Fatima Bibi, Naheeda Bibi, Salma Bibi, Tihana Bicanic, Sarah Bidgood, Julie Bigg, Sarah Biggs, Alphonsa Biju, Andras Bikov, Sophie Billingham, Jessica Billings, Alice Binns, Muhammad BinRofaie, Oliver Bintcliffe, Catherine Birch, Janine Birch, Jenny Birch, Katherine Birchall, Sam Bird, Sumedha Bird, Sarah Bird, Mark Birt, Claire Bishop, Kilanalei Bishop, Linda Bishop, Lisa Bishop, Karen Bisnauthsing, Nibedan Biswas, Sahar Biuk, Karen Blachford, Ethel Black, Helen Black, Karen Black, Mairead Black, Polly Black, Virginia Black, Hayley Blackgrove, Bethan Blackledge, Joanne Blackler, Samantha Blackley, Helen Blackman, Caroline Blackstock, Cameron Blair, Francesca Blakemore, Helen Blamey, Alison Bland, Sujata Blane, Simon Blankley, Parry Blaxill, Katie Blaylock, Jane Blazeby, Natalie Blencowe, Ben Bloom, Jack Bloomfield, Angela Bloss, Alex Blowers, Samuel Blows, Hannah Bloxham, Susan Blrd, Louise Blundell, Andrew Blunsum, Mark Blunt, Tadhg Blunt, Ian Blyth, Kevin Blyth, Andrew Blythe, Karen Blythe, Marilyn Boampoaa, Sarah Board, Boniface Bobie, Karen Bobruk, Pritesh N Bodalia, Neena Bodasing, Marianne Boden, Tanya Bodenham, Gabriele Boehmer, Marta Boffito, Katherine Bohanan, Kristyna Bohmova, Niamh Bohnacker, Sumit Bokhandi, Maria Bokhar, Saba Bokhari, Sakina Bokhari, Syed O Bokhari, Idan Bokobza, Ambrose Boles, Clare Bolger, Charlotte Bond, Helena Bond, Stuart Bond, Thomas Bond, Alice Bone, Georgia Boniface, Lizzy Bonney, Lauren Booker, Sarah Boot, Matthew Boothroyd, Joanne Borbone, Naomi Borman, Samantha Bosence, Kirsty Bostock, Neil Botting, Fiona Bottrill, Hannah Bouattia, Laura Bough, Hayley Boughton, Zoe Boult, Tarek Boumrah, Miriam Bourke, Stephen Bourke, Michelle Bourne, Rachel Bousfield, Lucy Boustred, Alexandra Bowes, Amy Bowes, Philip Bowker, Tabitha Bowker, Helen Bowler, Louise Bowman, Simon Bowman, Rachel Bowmer, Angie Bowring, Helen Bowyer, Aileen Boyd, Jenny Boyd, Laura Boyd, Nicola Boyer, Namoi Boyle, Pauline Boyle, Rosalind Boyle, Louise Boyles, Leanna Brace, Abbey Bracken, Jodie Bradder, Clare J Bradley, Pamela Bradley, Patrick Bradley, Paul Bradley, Joanne Bradley-Potts, Lynne Bradshaw, Zena Bradshaw, Clare Brady, Rebecca Brady, Shirin Brady, Pedro Braga Sardo, Denise Braganza, Megan Braithwaite, Susan Brammer, Marie Branch, Thomas Brankin-Frisby, Jamie Brannigan, Sophie Brattan, Fiona Bray, Nancy Bray, Manny Brazil, Lucy Brear, Tracy Brear, Stephen Brearey, Laura Bremner, Morwenna Brend, Carolin Bresges, Catherine Bressington, Giovanna Bretland, Chris Brewer, Michael Bridgett, Gavin Bridgwood, Sara Brigham, John Bright, Christopher Brightling, Tracey Brigstock, Lutece Brimfield, Philip Brinksman, Elaine Brinkworth, Robin Brittain-Long, Vianne Britten, Hannah Britton, Lauren Broad, Sarah Broadhead, Rosie Broadhurst, Andrew Broadley, Marie Broadway, Christopher Brockelsby, Megan Brocken, Tomos Brockley, Mary Brodsky, Fiona Brogan, Liz Brohan, Felicity Brokke, Jacob Brolly, David Bromley, Hannah Brooke-Ball, Verity Brooker, Matthew Brookes, Deirdre Brooking, Alison Brooks, Daniel Brooks, Jolene Brooks, Karen Brooks, Nicole Brooks, Philip Brooks, Rachel Brooks, Samuel Brooks, Sophie Brooks, Malcolm Broom, Natalie Broomhead, Chloe Broughton, Nathaniel Broughton, Matt Brouns, Alison Brown, Ammani Brown, Andrew Brown, Ashley Brown, Caitlin Brown, Carly Brown, Catrin Brown, Ellen Brown, Heather Brown, Janet Brown, Jo Brown, Louise Brown, Niall Brown, Nicola Brown, Pauline Brown, Richard Brown, Robert Brown, Steven Brown, Tom Brown, Bria Browne, Charlotte Browne, Duncan Browne, Mitchell Browne, Stephen Brownlee, Alba Brraka, Johanna Bruce, Michelle Bruce, Wojciech Brudlo, Andrei Brunchi, Nigel Brunskill, Alan Brunton, Margaret Brunton, Meera Bryant, Emma Bryden, Hannah Brzezicki, April Buazon, Maya H Buch, Ruaridh Buchan, Danielle Buche, Amanda Buck, Lisa Buck, Matthew Buckland, Clare Buckley, Laura Buckley, Philip Buckley, Sarah Buckley, Carol Buckman, Alex Budds, George Bugg, Ramadan Bujazia, Marwan Bukhari, Shanze Bukhari, Richard Bulbulia, Alex Bull, Damian Bull, Kate Bull, Rhian Bull, Thomas Bull, Emily Bullock, Susan Bullock, Naomi Bulteel, Kasun Bumunarachchi, Roneleeh Bungue-Tuble, Oliver Burbidge, Caroline Burchett, Dorota Burda, Christy Burden, Thomas G Burden, Mika Burgess, Richard Burgess, Sophia Burgess, Hasan Burhan, Hannah Burke, Kerry Burke, Adrian Burman, Sara Burnard, Caroline Burnett, Sharon Burnett, Amy Burns, Collette Burns, James Burns, Karen Burns, Kiki Burns, Daniel Burrage, Kate Burrows, Claire Burston, Anna Burton, Ben Burton, Fiona Burton, Helen Burton, Matthew Burton, Mannaria Butar butar, Deborah Butcher, Aaron Butler, Emily Butler, Jessica Butler, Joanne Butler, Joshua Butler, Peter Butler, Susan Butler, Jenny Butler, Al-Tahoor Butt, Massawer Butt, Mohammad M Butt, Caryl Butterworth, Nicola Butterworth-Cowin, Robert Buttery, Tom Buttle, Heather Button, Daniel Buttress, Hannah Bye, Jane Byrne, Wendy Byrne, Victoria Byrne-Watts, Amanda Cabandugama, Luisa Cabrero, Simone Caddy, Ruth Cade, Anthony Cadwgan, Zhen Cahilog, Ajeng Cahyareny, Donna Cairney, James Calderwood, Darren Caldow, Emily Cale, Giorgio Calisti, Debbie Callaghan, Jennifer Callaghan, Claire Callens, Donaldson Callum, Caroline Calver, Melissa Cambell-Kelly, Tracey Camburn, David R Cameron, Eleanor Cameron, Fraser Cameron, Sheena Cameron, Christian Camm, Renee F D Cammack, Alison Campbell, Amy Campbell, Barbara Campbell, Bridget Campbell, Debbie Campbell, Helen Campbell, Hilary Campbell, Jonathan Campbell, Karen Campbell, Marion Campbell, Mark Campbell, Rachael Campbell, Robyn Campbell, Wynny Campbell, Quentin Campbell Hewson, Julie Camsooksai, Lisa Canclini, Shaula Mae Candido, Janie Candlish, Cielito Caneja, Alexandra Cann, Johnathon Cann, Ruby Cannan, Abigail Cannon, Emma Cannon, Michael Cannon, Petra Cannon, Vivienne Cannons, Ebnier Canonizado, Jane Cantliff, Ben Caplin, Santino Capocci, Noemi Caponi, Angelika Capp, Richard Capstick, Thomas Capstick, Catalin Caraenache, Aimee Card, Mary Cardwell, Charles Carey, Rachel Carey, Simon Carley, Ffion Carlin, Tammy Carlin, Samantha Carmichael, Margaret Carmody, Mandy Carnahan, Charlotte Caroline, Jodi Carpenter, Sharon Carr, Anna Carrasco, Zoe Carrington, Amy Carroll, Anne Carroll, Paul Carroll, Rachel Carson, Cindy Cart, Emma Carter, Jonathan Carter, Michael Carter, Natasha Carter, Paul Carter, Penny Carter, Douglas Cartwright, Jo-Anne Cartwright, Claire Carty, Lotoyah Carty, Jaime Carungcong, Charlotte Carver, Emma Carver, Rachel Carver, Susan Casey, Annie Cassells, Teresa Castiello, Gail Castle, Bridget Castles, Melanie Caswell, Ana Maria Catana, Heidi Cate, Anelise Catelan Zborowski, Susanne Cathcart, Katrina Cathie, Darwin Catibog, Christine Catley, Laura Catlow, Matthew Caudwell, Anna Cavazza, Ashleigh Cave, Luke Cave, Simon Cavinato, Frianne Cawa, Kathryn Cawley, Chloe Caws, Kay Cawthorne, Hankins Cendl, Hannah Century, Jeva Cernova, Mansur Cesay, Ed Cetti, Stephanie Chabane, Manish Chablani, Cathleen Chabo, Cyril Jacob Chacko, David Chadwick, Julie Chadwick, Robert Chadwick, Ela Chakkarapani, Arup Chakraborty, Mallinath Chakraborty, Mollika Chakravorty, Petya Chalakova, Bimal S Chalise, James Chalmers, Richard Chalmers, Georgina Chamberlain, Sarah Chamberlain, Emma Chambers, Jonathan Chambers, Lucy Chambers, Naomi Chambers, Alex Chan, Carmen Chan, Cheuk Chan, Evelyn Chan, Jay M Chan, Kawai Chan, Kayen Chan, Kimberley Chan, Ping Chan, Rebekah (Pui-Ching) Chan, Xin Hui S Chan, Chris Chandler, Heidi Chandler, Kim J Chandler, Stuart Chandler, Zoe Chandler, Sumit Chandra, Navin Chandran, Badrinathan Chandrasekaran, Yvonne Chang, Grace Chaplin, Josephine Chaplin, Graeme Chapman, John Chapman, Katie Chapman, Laura Chapman, Lianne Chapman, Matthew Chapman, Polly Chapman, Timothy Chapman, Lucy Chappell, Amanda Charalambou, Bethan Charles, Dianne Charlton, Sam Charlton, Kevin Chatar, Calvin Chatha, Debra Chatterton, Ritesh Chaube, Muhammad Y N Chaudhary, Iram Chaudhry, Zain Chaudhry, Nazia Chaudhuri, Muhammad Chaudhury, Anoop Chauhan, Ruchi S Chauhan, Alexander Chavasse, Nicola Chavasse, Vipal Chawla, Lindsay Cheater, James Cheaveau, Charlotte Cheeld, Michelle Cheeseman, Fang Chen, Hui Min Chen, Terence Chen, Floria Cheng, Lok Yin Cheng, Zhihang Cheng, Helen Chenoweth, Chun How Cheong, Shiney Cherian, Mary Cherrie, Helen Cheshire, Chee Kay Cheung, Elaine Cheung, Kayan Cheung, Michelle Cheung, Claire Cheyne, Swati Chhabra, Wei Ling Chia, Eric Chiang, Angela Chiapparino, Rosavic Chicano, Moses Chikungwa, Zviedzo A Chikwanha, Gemma Chilcott, Sam Chilcott, Alison Chilvers, Phillipa Chimbo, KokWai Chin, Wen Jie Chin, Rumbidzai Chineka, Amol Chingale, Ezomike Chinonso, Chloe Chin-Saad, Margaret Chirgwin, Heather Chisem, Claire Chisenga, Catherine Chisholm, Ben Chisnall, Carolyn Chiswick, Sunder Chita, Nihil Chitalia, Matthew Chiu, Laura Chiverton, Brenda Chivima, Catherine Chmiel, Soha Choi, Willy Choon Kon Yune, Meena Chopra, Michelle Chopra, Vandana Choudhary, Omur Choudhury, Sarah Choudhury, Bing-Lun Chow, Mahibbur Chowdhury, Shahid Chowdhury, Angela Chrisopoulou, Victoria Christenssen, Peter Christian, Alexander Christides, Fiona Christie, Daniel Christmas, Georgios Christoforou, Thereza Christopherson, Anna Christou, Mark Christy, Paris Chrysostomou, Yunli Chua, Dip Chudgar, Richard Chudleigh, Srikanth Chukkambotla, Michael E Chukwu, Izu Chukwulobelu, Chi Yee Chung, Elaine Church, Sara R Church, David Churchill, Nicole Cianci, Paola Cicconi, Paola Cinardo, Zdenka Cipinova, Bessie Cipriano, Sarah Clamp, Bridget Clancy, Melanie Clapham, Edel Clare, Sarbjit Clare, Andrew Clark, Charlotte Clark, Diane Clark, Emma Clark, Felicity Clark, Gabrielle Clark, James Clark, Julia Clark, Katherine Clark, Kaylea Clark, Louise Clark, Lucy Clark, Martyn Clark, Matthew Clark, Nicola Clark, Patricia Clark, Richard Clark, Thomas Clark, Tonia Clark, Zoe Clark, Alison Clarke, Andrea Clarke, Jude Clarke, Paul Clarke, Robert Clarke, Roseanne Clarke, Samantha Clarke, Sheron Clarke, Alleyna Claxton, Louis Claxton, Kate Clay, Catherine Clayton, Elizabeth Clayton, Olivia Clayton, Jill Clayton-Smith, Bethan Clearyb, Chris Cleaver, Rose Cleeton, Ian Clement, Carlota Clemente de la Torre, Jayne Clements, Suzanne Clements, Sue Clenton, Sheryl Cliff, Rachael Clifford, Sarah Clifford, Amelia Clive, Jonathan Clouston, Vincent Clubb, Samantha Clueit, Lisa Clutterbuck, Andrea Clyne, Michelle Coakley, Peter G L Coakley, Kathryn Cobain, Alexandra Cochrane, Patricia Cochrane, Laura Cockayne, Maeve Cockerell, Helen Cockerill, Shirley Cocks, Rachel Codling, Adam Coe, Samantha Coetzee, David Coey, Danielle Cohen, Jonathan Cohen, Oliver Cohen, David Cohen, Mike Cohn, Louise Coke, Olutoyin Coker, Nicholas Colbeck, Roghan Colbert, Esther Cole, Jade Cole, Joby Cole, Garry Coleman, Matt Coleman, Nick Coleman, Holly Coles, Macleod Colin, Alicia Colino-Acevedo, Julie Colley, Kathleen Collie, Alexandra Collier, Danielle Collier, Dawn Collier, Heather Collier, Terry Collingwood, Paul Collini, Emma Collins, Jaimie Collins, Joanne Collins, Kayleigh Collins, Megan Collins, Nicola Collins, Sally Collins, Vicky Collins, Andrew Collinson, Bernadette Collinson, Jennifer Collinson, Matthew Collis, Madeleine Colmar, Hayley E Colton, James Colton, Katie Colville, Carolyn Colvin, Edward Combes, David Comer, Alison Comerford, Dónal Concannon, Alison Condliffe, Robin Condliffe, Emma Connell, Lynne Connell, Natalie Connell, Karen Connelly, Gavin Connolly, Emma Connor, Antonia Conroy, Kevin Conroy, Veronica Conteh, Rory Convery, Francesca Conway, Grainne Conway, Rhiannon Conway, Robert Conway, Jo-Anna Conyngham, Amanda Cook, Amber Cook, Colette Cook, Eloise Cook, Gemma Cook, Helen Cook, Julie Cook, Martin Cook, Sarah Cook, Danielle Cooke, Graham Cooke, Hannah Cooke, Justin Cooke, Katrina Cooke, Tim Cooke, Vikki Cooke, Adele Cooper, Chris Cooper, David Cooper, Helen Cooper, Jamie Cooper, Lauren Cooper, Nick Cooper, Rowena Cooper, Samantha Cooray, Thomas Cope, Sinead Corbet, Carolyn Corbett, Ailsa Corbishley, John Corcoran, Chris Cordell, Jessica Cordle, Alasdair Corfield, John Corless, Alison Corlett, Joe Cornwell, Michael Cornwell, Diana Corogeanu, Aisling Corr, Mirella Corredera, Ruth Corrigan, P Corry, Rita Corser, Jon Cort, Denise Cosgrove, Tracey Cosier, Patricia Costa, Telma Costa, Charlie Coston, Susannah Cotgrove, Zoe Coton, Lisa-Jayne Cottam, Rhiannon Cotter, Donna Cotterill, Caroline Cotton, George Couch, Martina Coulding, Andrew Coull, David Counsell, David Counter, Cherry Coupland, Ellie Courtney, Julia Courtney, Rebecca Cousins, Amanda J Coutts, Alexander Cowan, Elena Cowan, Richard Cowan, Richard Cowell, Louise Cowen, Steve Cowman, Amanda Cowton, Ellie Cox, Emma Cox, Giles Cox, Helen Cox, Karina Cox, Miriam Cox, Karen Coy, Andrea Cradduck-Bamford, Hannah Craig, Jayne Craig, John Craig, Victoria Craig, Felicity Craighead, Matthew Cramp, Hayley Cranston, Sheetal S Crasta, Jacolene Crause, Angie Crawford, Emma Crawford, Isobel Crawford, Sarah Crawshaw, Ben Creagh-Brown, Andrew Creamer, Amanda Creaser-Myers, Joanne Cremona, Saveria Cremona, Anna Crepet, Janet Cresswell, Mark Cribb, Charles Crichton, Declan Crilly, Lauren Crisp, Nikki Crisp, Dominic Crocombe, Maria Croft, Jennifer Crooks, Harriet Crosby, Elizabeth Cross, Tim Cross, Amy Crothers, Stephen Crotty, Susan Crouch, Madeleine Crow, Amanda Crowder, Kate Crowley, Teresa Crowley, Rebecca Croysdill, Callum Cruickshank, Conor Cruickshank, Irena Cruickshank, James Cruise, Carina Cruz, Trino Cruz Cervera, Dominic Cryans, Guanguo Cui, Helen Cui, Lorraine Cullen, Gillian Cummings-Fosong, Victoria Cunliffe, Neil Cunningham, Nicola Cunningham, Jason Cupitt, Hollie Curgenven, Gerens Curnow, David Curran, Simon Curran, Craig Currie, Jacqueline Currie, Scarlett Currie, Susan Currie, Susie Currie, Jonathan Curtis, Katrina Curtis, Matthew Curtis, Olivia Curtis, Thomas Curtis, Rebecca Cuthbertson, Jennifer Cuthill, Sean Cutler, Sarah Cutts, Marta Czekaj, Patrycja Czylok, Shane D'Souza, Joana da Rocha, Mohammed Dafalla, Andrew Dagens, Helen Daggett, Jacqui Daglish, Sandeep Dahiya, Anne Dale, Katie Dale, Michaela Dale, Sam Dale, Susan Dale, Jolyon Dales, Helen Dalgleish, Helen Dallow, Carlo D'aloia, Dermot Dalton, Morag Dalton, Zoe Daly, Mansi Damani, Eleanor Damm, Akila Danga, Joanne Dangerfield, Amelia Daniel, Priya Daniel, Allison Daniels, Adela Dann, Sandra Danso-Bamfo, Suzanne Darby, Alex Darbyshire, Janet Darbyshire, Paul Dargan, Paul Dark, Kate Darlington, Sarah Darnell, Tom Darton, Guledew Darylile, Avisnata Das, Manjusha Das, Sukamal Das, Martin Daschel, Joanne Dasgin, Dibyendu Datta, Anna Daunt, Vaishali Dave, Emily Davenport, Mark Davey, Miriam Davey, Molly Davey, Mini David, Alexander Davidson, Laura Davidson, Neil Davidson, Richard Davidson, Albert Davies, Alison Davies, Amanda Davies, Amy Davies, Angela Davies, Bethan Davies, Caroline Davies, Carolyn Davies, Catrin Davies, Dawn Davies, Drew Davies, Elaine Davies, Elinor Davies, Ellie Davies, Ffyon Davies, Gwyneth Davies, Helen Davies, Jennifer Davies, Jim Davies, Karen Davies, Kelly Davies, Kim Davies, Llinos Davies, Louisa Davies, Matthew Davies, Michelle Davies, Nina Davies, Owen Davies, Patrick Davies, Rachel Davies, Rhys Davies, Ruth Davies, Sally Davies, Sarah Davies, Simon Davies, Sue Davies, Alison Davis, Julie-Ann Davis, Katherine Davis, Peter Davis, Andrea Davis-Cook, Alexander Davison, Christine Dawe, H Dawe, Mark Dawkins, Angela Dawson, Danielle Dawson, Elizabeth Dawson, Joy Dawson, Lucinda Dawson, Mike Dawson, Susan Dawson, Tom Dawson, Isla Dawson, Andrew Daxter, Andrew Day, Jacob Day, Jeremy N Day, Jamie D'Costa, Parijat De, Pradip De, Duneesha de Fonseka, Toni de Freitas, Roberta De Pretto, Frederico De Santana Miranda, Eleanor de Sausmarez, Shanika de Silva, Thushan de Silva, Jessica De Sousa, Paulo De Sousa, James de Souza, Phillip De Souza, Anthony De Soyza, Natasha de Vere, Johannes de Vos, Bethan Deacon, Sharon Dealing, Anna Dean, Julie Dean, Katrina Dean, Stephen Dean, Tessa Dean, Jill Deane, James Dear, Effie Dearden, Catherine Deas, Samuel Debbie, Gabor Debreceni, Vashist Deelchand, Matthew Deeley, Joanne Deery, Emmanuel Defever, Manuela Del Forno, Arnold Dela Rosa, Gonzalo De-La-Cedra, Amanda Dell, Pamela De Los Santos Dominguez, Carrie Demetriou, David DeMets, Jane Democratis, Jacqueline Denham, Emmanuelle Denis, Laura Denley, Craig Denmade, Alexandra Dent, Kathy Dent, Martin Dent, Elise Denton, Tom Denwood, Nishigandh Deole, Darshita Depala, Maria Depante, Susan Dermody, Amisha Desai, Asmita Desai, Purav Desai, Sanjeev Deshpande, Vai Deshpande, Sirjana Devkota, Usha Devkota, Debbie Devonport, Matthew Devonport, Prakash Dey, Vishal Dey, Rogin Deylami, Kevin Dhaliwal, Sundip Dhani, Amandeep Dhanoa, Mili Dhar, Ankush Dhariwal, Dawpadee Dharmasena, Devesh Dhasmana, Ekanjali Dhillon, Reiss Dhillon, Syra Dhillon, Meghnath Dhimal, Dipali Dhiru, Trupti Dhorajiwala, Priya Dias, Stephanie Diaz, Kayleigh Diaz-Pratt, Mohammed Dibas, Debbie Dickerson, Pamela Dicks, Matt Dickson, Stuart Dickson, J Digby, Ronneil Digpal, Sean Dillane, Sarah Diment, Paul Dimitri, Georgios Dimitriadis, Sajeel Din, Thai Ha Dinh, Alex Dipper, Sabiq Dipro, Laura Dirmantaite, Lorelle Dismore, Lisa Ditchfield, Sarah Diver, Lavanya Diwakar, Preeti Diwan, Caroline Dixon, Giles Dixon, Kate Dixon, Brice Djeugam, Petr Dlouhy, Andrea D'Mello, Paul Dmitri, Laurence Dobbie, Marinela Dobranszky Oroian, Charlotte Dobson, Lee Dobson, Marie Docherty, David Dockrell, Jacqueline Dodd, James Dodd, Jackie Dodds, Rebecca Dodds, Steve Dodds, Richi Dogra, Conor Doherty, Erin Doherty, Warren Doherty, Yumiko Doi, Iain Doig, Eleanor Doke, Daniel Dolan, Mark Dolman, Rozzie Dolman, Lisa Donald, Katie Donald, Callum Donaldson, Christopher Donaldson, Denise Donaldson, Gillian Donaldson, Kate Donaldson, Sinead Donlon, Joanne Donnachie, Eilish Donnelly, Ronan Donnelly, Phil Donnison, Aravindhan Donohoe, Gemma Donohoe, Bryan Donohue, Emma Dooks, Reece J Doonan, Grainne Doran, Rachael Dore, Kane Dorey, Sharon Dorgan, Kataryna Dos Santos, Moonira Dosani, Davinder Dosanjh, Paula Dospinescu, Isabelle Doss, Theonymfi Doudouliaki, Andrew Dougherty, Jill Doughty, Katie Douglas, Jonathan Douse, Allyn Dow, Lucy Dowden, Michelle Dower, Sud Dowling, Nicola Downer, Charlotte Downes, Rob Downes, Thomas Downes, Damian Downey, Robert Downey, Caroline Downing, Louise Downs, Simon Dowson, Cornel Dragan, Cristina Dragos, Maire Drain, Chelsea Drake, Victoria Drew, Olivia Drewett, Anthony Drexel, Celine Driscoll, Helena Drogan, Odysseas Drosos, Graham Drummond, Katie Drury, Kay Druryk, Ronald Druyeh, Jack Dryburgh-Jones, Simon Drysdale, An Du Thinh, Hazel Dube, Judith Dube, Stephen Duberley, Paige K Duckenfield, Hayley Duckles-Leech, Nicola Duff, Emma Duffield, Heather Duffy, Helen Duffy, Katharine Duffy, Lionel Dufour, Annette Duggan, Parveen Dugh, Rauand Duhoky, Janice Duignan, Jennifer Dulay, Simon Dummer, Alexis Duncan, Andrew Duncan, Christopher Duncan, Fullerton Duncan, Gregory Duncan, Holly Duncan, Robert Duncan, Stephanie Dundas, Aileen Dunleavy, Julia Dunleavy, Alessia Dunn, Charlotte Dunn, Damian Dunn, Laura Dunn, Paul Dunn, Charlene Dunne, Karen Dunne, Fiona Dunning, Aidan Dunphy, Venkat Duraiswamy, Beatriz Duran, Ingrid DuRand, Leanne Durdle, Natalie Duric, Alison Durie, Emily Durie, Samuel Durogbola, Chris Durojaiye, Laura Durrans, Katie Durrant, Hannah Durrington, Iheukwumere Duru, Haris Duvnjak, Akshay Dwarakanath, Laasya Dwarakanath, Ellen Dwyer, Zara Dyar, Claudia Dyball, Kristyn Dyer, Harvey Dymond, Tom Dymond, Chris Eades, Lauren Eadie, Rebekah Eadie, Laura Eagles, Beena Eapen, Naomi Earl, Joanne Early, Melissa Earwaker, Nicholas Easom, Clare East, Amy Easthope, Fraser Easton, Jack Easton, Patrick Easton, Ruth Eatough, Oluwadamilola Ebigbola, Martin Ebon, Alison Eccles, Sinan Eccles, Chloe Eddings, Michael Eddleston, Maureen Edgar, Katharine Edgerley, Nicholas Edmond, Mary Edmondson, Tracy Edmunds, Abby Edwards, Alexandra Edwards, Catherine Edwards, Joy Edwards, Kennedy Edwards, Mandy Edwards, Martin Edwards, Sophie Edwards, Jenny Eedle, Aline Eggink, Dawn Egginton, Sam Eggleston, Loveth Ehiorobo, Sarah Eisen, Ugochukwu Ekeowa, Mohamed Ekoi, Ayomide Ekunola, Soha El Behery, Nagla Elashhar, Lisa Elawamy, Mohamed Elbeshy, Kate El-Bouzidi, Jennifer Elder, Mohammed El-Din, Emma Eldridge, Diana Eleanor, Uchenna Elenwa, Ibrahim Eletu, Eman Elfar, Mayy M Elgamal, Amr Elgohary, Mohamed A A Elhadi, Stellios Elia, Jennifer Elias, Tania Elias, Nadia Elkaram, Andrew V Elkins, Julie Ellam, Nikki Ellard, Laura N Ellerton, Lucy Elliot, Amy Elliott, Fiona Elliott, Kerry Elliott, Scott Elliott, Sian Elliott, Annie Ellis, Christine Ellis, Kaytie Ellis, Leila Ellis, Rupert Ellis, Tak-Yan Ellis, Yvette Ellis, Alison C Ellwood, Rahma Elmahdi, Einas Elmahi, Hannah-May Elmasry, Mohammed El-Naggar, Najla Elndari, Omer Elneima, Mohamed Elokl, Ahmed Elradi, Mohamed Elsaadany, Mohammed A S A Elsayed, Sally El-Sayeh, Hana El-Sbahi, Mohammad Elsebaei, Tarek Elsefi, Karim El-Shakankery, Alguili Elsheikh, Hosni El-Taweel, Sarah Elyoussfi, Jonathan Emberey, Jonathan R Emberson, John Emberton, Anna Emery, Julian Emmanuel, Ingrid Emmerson, Michael Emms, Florence Emond, Marieke Emonts, Nicu Enachi, Dickson Enenche, Angila Engden, Katy English, Emma Entwistle, Hene Enyi, Marios Erotocritou, Peter Eskander, Hanif Esmail, Fatheha Essa, Brynach Evans, Chris Evans, Debra Evans, Emrys Evans, Gail Evans, Gareth Evans, Isabel Evans, Jack Evans, Jacqueline Evans, Jennifer Evans, John Evans, Jolanta Evans, Lisa Evans, Lynn Evans, Mim Evans, Morgan Evans, Ranoromanana Evans, Sharon Evans, Sian Evans, Teriann Evans, Tomos Evans, Caroline Everden, Serenydd Everden, Lynette Every, Hayley Evison, Lynsey Evison, Chiamaka Ezenduka, Jacqueline Faccenda, Leila Fahel, Youstina Fahmay, Ian Fairbairn, Sara Fairbairn, Terry Fairbairn, Andy Fairclough, Louise Fairlie, Mark Fairweather, Anne Fajardo, Naomi Falcone, Euan Falconer, John Fallon, Andrea Fallow, David Faluyi, Victoria Fancois, Ayaan Farah, Muna Farah, Qayyum Farah, Nowin Z Fard, Leila Fares, Amr Farg, Adam Farmer, Katie Farmer, Toni Farmery, Samantha Farnworth, Faiyaz Farook, Hadia Farooq, Sidrah Farooq, Fiona Farquhar, Holly Farr, Aaron Farrell, Barbara Farrell, Fayed Farrukh, James Farthing, Syeda Farzana, Rahmatu Fasina, Azam Fatemi, Mina Fatemi, Suman Fathima, Nibah Fatimah, Maria Faulkner, Saul N Faust, Clair Favager, Joe Fawke, Sinmidele Fawohunre, Abul Fazal, Aisthath Fazleen, Simon Fearby, Connie Fearnley, Alex Feben, Federico Fedel, Daria Fedorova, Christopher Fegan, Mae Felongo, Lynsey Felton, Tim Felton, Kate Fenlon, Andrea Fenn, Ruth Fennelly, Isabelle Fenner, Ciara Fenton, Melisa Fenton, Gloria Ferenando, Cameron Ferguson, Jenny Ferguson, Kathryn Ferguson, Katie Ferguson, Susan Ferguson, Susie Ferguson, Victoria Ferguson, Denzil Fernandes, Candida Fernandez, Eduardo Fernandez, Maria Fernandez, Sonia Fernandez Lopez, Jaime Fernandez Roman, Callum J Fernando, Jessica Fernando, Ahmed Feroz, Pietro Ferranti, Thais Ferrari, Eleanor Ferrelly, Alexandra Ferrera, Emma Ferriman, Sheldon Ferron, Nicholas Fethers, Ben Field, Janet Field, Rebecca Field, Karen Fielder, Lindsey Fieldhouse, Andra Fielding, Julie Fielding, Sarah Fielding, Asma Fikree, Santos Filipa, Sarah A Filson, Shona Finan, Sarah Finbow, Deborah J Finch, Joanne Finch, Laurie Finch, Simon Finch, Spencer Finch, Natalie Fineman, Jim Finlayson, Lauren Finlayson, Adam Finn, Joanne Finn, Dylan Finnerty, Clare Finney, Deborah Finucane, Sofia Fiouni, Jo Fiquet, Promise Firi, James Fisher, Neil Fisher, Daniel Fishman, Krystofer Fishwick, Carolyn Fitton, Felicity Fitzgerald, Fiona Fitzgerald, Karen Fitzjohn, Jan Flaherty, Michael Flanagan, Charles Flanders, Nikolaos Flaris, Gail Fleming, Julie Fleming, Lucy Fleming, Paul Fleming, William Flesher, Alison Fletcher, Anna Fletcher, Jonathan Fletcher, Lucy Fletcher, Simon Fletcher, Sophie Fletcher, Fiona Flett, Karen Flewitt, Sarah Flockhart, Christopher Flood, Ian Floodgate, Jonathan Flor, Vincent Florence, Mary Flowerdew, Sharon Floyd, Margaret J Flynn, Rachel Flynn, Claire Foden, Adama Fofana, Georgina Fogarty, Paul Foley, Linda Folkes, Tracey Fong, Daniela M Font, Aiwyne Foo, Jane Foo, Andrew Foot, Helen R Foot, Jayne Foot, Jane Forbes, Amber Ford, Jamie Ford, Imogen Fordham, Jennifer Foreman, Matthew Forester, Mohammad Forkan, Caroline Fornolles, Adam Forrest, Ellie Forsey, Miranda Forsey, Thomas Forshall, Elliot Forster, Abigail Forsyth, Julian Forton, Conrad Foster, Emily Foster, Joseph Foster, Rachel A Foster, Tracy Foster, Angela Foulds, Ian Foulds, Folakemi Fowe, Natasha Fowkes, Emily Fowler, Robert Fowler, Stephen Fowler, Amy Fox, Claire Fox, Heather Fox, Jonathan Fox, Laura Fox, Lauren Fox, Natalie Fox, Olivia Fox, Simon Fox, Sarah-Jane Foxton, Eva Fraile, Rebecca Frake, Alex Francioni, Olesya Francis, Rebecca Francis, Sarah Francis, Theodora Francis-Bacon, Helen Frankland, Jessica Franklin, Siobhan Franklin, Catherine Fraser, Andi Fratila, Sharon Frayling, Martyn Fredlund, Anna Freeman, Carol Freeman, Elaine Freeman, Hannah Freeman, Nicola Freeman, Clare Freer, Eleanor French, Thomas French, Karen Freshwater, Matthew Frise, Renate Fromson, Adam Frosh, John Frost, Victoria Frost, Oliver Froud, Rachel Frowd, Arun Fryatt, Andrzej Frygier, Bridget Fuller, Liz Fuller, Lucy Fuller, Tracy Fuller, Duncan Fullerton, Carrie Fung, Gayle Fung, Sarah Funnell, John Furness, Andrew Fyfe, Nytianandan G, Elizabeth Gabbitas, Claire Gabriel, Zoë Gabriel, Hadiza Gachi, Sophie Gaffarena, Sarah Gage, Joshua Gahir, Sarveen Gajebasia, Katarzyna Gajewska-Knapik, Zacharoula Galani, Christopher Gale, Hugo Gale, Linda Gale, Rebecca Gale, Swetha Gali, Bernadette Gallagher, Jude Gallagher, Rosie Gallagher, William Gallagher, Feroze Gallam, Joanne Galliford, Catherine Galloway, Chris Galloway, Emma Galloway, Jacqui Galloway, James Galloway, Anna Galvin, Victoria Galvis, Gareth Gamble, Laura Gamble, Liz Gamble, Brian Gammon, Chien Nee Gan, Muhammad B Ganaie, Jaikumar Ganapathi, Ramesh Ganapathy, Kaminiben Gandhi, Sarah Gandhi, Usha Ganesh, Thenugan Ganeshanathan, Saibal Ganguly, Abrar Gani, Pauline Ganley, Ukraina Garcia, Emma-James Garden, Antoni D Gardener, Emma Gardiner, Michael Gardiner, Phil Gardiner, Siobhan Gardiner, Caroline Gardiner-Hill, Jonathan Gardner, Louis Gardner, Mark Garfield, Atul Garg, Isha Garg, Nathan Garlick, Daniel Garner, Justin Garner, Lucie Garner, Zoe Garner, Rosaline Garr, Kathryn A Garrero, Florence Garty, Rachel Gascoyne, Hyeriju Gashau, Aoife Gatenby, Erin Gaughan, Alok Gaurav, Mariana Gavrila, Jane Gaylard, Sophie Gayle, Catherine Geddie, Ian Gedge, Sarah Gee, Fartuun Geele, Kalaichelvi Geerthan, Minnie Gellamucho, Karzan Gelly, Leila Gelmon, Sandra Gelves-Zapata, Gemma Genato, Neal Gent, Susan Gent, Natalie Geoghegan, Aparna George, Bini George, Sam George, Susan George, Tina George, Valerie P George, Simon Georges, Domonique Georgiou, Peter Gerard, Leigh Gerdes, Louise Germain, Helen Gerrish, Abel Getachew, Louise Gethin, Siobhan Gettings, Hisham Ghanayem, Bijan Ghavami Kia, Shaista Ghazal, Anca Gherman, Alison Ghosh, Dipansu Ghosh, Justin Ghosh, Sudhamay Ghosh, Sarra Giannopoulou, Malick Gibani, Chris Gibb, Ben Gibbison, Kerry Gibbons, Alex Gibson, Bethan Gibson, Jamie Gibson, Kimberley Gibson, Kirsty Gibson, Sian Gibson, Mary Gigi, Cat Gilbert, Jeanette Gilbert, Kayleigh Gilbert, Benjamin Giles, James Gilham, Mandy Gill, Lynne Gill, Paul Gillen, Annelies Gillesen, Katherine Gillespie, Elizabeth Gillham, Andrew Gillian, Deborah Gilliland, Robert Gillott, Danielle Gilmour, Kate Gilmour, Lesley Gilmour, Louis Ginn, Franciscus Ginting, Theodora Giokanini-Royal, Anna Gipson, Barbie Giri, Joanna Girling, Rhian Gisby, Angelena Gkioni, Aikaterini Gkoritsa, Effrossyni Gkrania-Klotsas, Amy Gladwell, James Glanville, Jessica Glasgow, Susannah Glasgow, Jon Glass, Lynn Glass, Sharon Glaysher, Lisa Gledhill, Eleanor Glenday, Ana Glennon, Jodie Glossop, John Glover, Kyle Glover, Michelle Glover, Jan Glover Bengtsson, Deborah Glowski, Sharon Glynn, Chevanthy Gnanalingam, Julie Goddard, Wendy Goddard, Emily Godden, Jo Godden, Sarah Godlee, Emma Godson, Gillian Godwin, Sukanya Gogoi, Aiky Goh, Manjinder Gohel, Rebeca Goiriz, Sriya Gokaraju, Raphael Goldacre, Arthur Goldsmith, Portia Goldsmith, Darren Gomersall, Lucia Gomez, Raquel Gomez-Marcos, Ali Gondal, Celia Gonzalez, Jack Goodall, Vicky Goodall, Bob Goodenough, Anna Goodfellow, Laura Goodfellow, James Goodlife, Camelia Goodwin, Elizabeth Goodwin, Jayne Goodwin, Paula Goodyear, Rajiv Gooentilleke, Michelle Goonasekara, Sheila Gooseman, Shameer Gopal, Chris Gordon, Sally Gordon, Robin Gore, Hugh Gorick, Caitlin Gorman, Claire Gorman, Stuart Gormely, Maria Gorniok, Diana Gorog, Michelle Gorst, Thomas Gorsuch, Jayshreebahen Gosai, Rebecca Gosling, Sally Gosling, Georgina Gosney, Vanessa Goss, Dzintars Gotham, Naomi Gott, Elizabeth Goudie, Nicole Gould, Susan Gould, Charlotte Goumalatsou, Lysander Gourbault, Abha Govind, Rajesh Govindan, Sharon Gowans, Girish Gowda, Rohit Gowda, Hannah Gower, Pankaj Goyal, Sunil Goyal, Sushant Goyal, Cheryl Graham, Clive Graham, Jonathan Graham, Justin Graham, Libby Graham, Rachel Graham, Sharon Graham, Matthew Graham-Brown, Julia Grahamslaw, Gianluca Grana, Tracyanne Grandison, Louis Grandjean, Alison Grant, Ann Grant, David Grant, Kirstie Grant, Matthew Grant, Pauleen Grant, Rhys Gravell, Jenny Graves, Alasdair Gray, Catherine Gray, Georgina Gray, Glaxy Gray, Jackie Gray, Karen Gray, Nicola Gray, Roxanne Gray, Sebastian Gray, Alan Grayson, Fiona Greaves, Paul Greaves, Alastair Green, Amy Green, Amy S Green, Charlotte Green, Christopher A Green, David Green, Frederick Green, Joel Green, Marie Green, Nicola Green, Stacey Green, Diarra Greene, Philippa Greenfield, Alan Greenhalgh, Daniel Greenwood, Sandra Greer, James Gregory, Jane Gregory, Katie Gregory, Tamsin Gregory, Jill Greig, Julia Greig, Rebecca Grenfell, Teena Grenier, Jack Grenville, Jo Gresty, Susan Grevatt, Glaxy Grey, Samuel Gribben, Andrew Gribbin, Amy Gribble, Natasha Grieg, Douglas Grieve, Ben Griffin, Denise Griffin, Mel Griffin, Sian Griffith, Andrew Griffiths, Daniel Griffiths, David Griffiths, Donna Griffiths, Isabel Griffiths, Mark Griffiths, Nicola Griffiths, Oliver Griffiths, Sandra Griffiths, Sarah Griffiths, Scott Griffiths, Yvonne Griffiths, Sofia Grigoriadou, Steph Grigsby, Paul Grist, Evelina Grobovaite, Dorothy Grogono, Clarissa Grondin, Rachel Groome, Pauline Grose, Liliana Grosu, Jenny Grounds, Margaret Grout, Helen Grover, Jayne Groves, Neil Grubb, Julie Grundy, Francesca Guarino, Sharada Gudur, Jacinta Guerin, Sharazeq Guettari, Shivang Gulati, Vikas Gulia, Harsha Gunasekara, Pumali Gunasekera, Malin Gunawardena, Kirun Gunganah, Jessica Gunn, Emma Gunter, Alok Gupta, Anup k Gupta, Atul Gupta, Rajeev Gupta, Richa Gupta, Rishi Gupta, Tarun Gupta, Vineet Gupta, Ankur Gupta-Wright, Victoria Guratsky, Alvyda Gureviciute, Sambasivarao Gurram, Anju Gurung, Bhawana Gurung, Lilly Gurung, Shraddha Gurung, Sabi Gurung Rai, Hazel Guth, Nick Guthrine, Pradip Gyanwali, Sushma Gyawali, Ruth Habibi, Berkin Hack, James Hackett, Pamela Hackney, Christian Hacon, Aiman Haddad, Denise Hadfield, Nicholas Hadfield, Sally Hadfield, Michalis Hadjiandreou, Nikolaos Hadjisavvas, Anna Haestier, Nauman Hafiz, Rana Hafiz-Ur-Rehman, Javed Hafsa, Samantha Hagan, Jack William Hague, Rosemary Hague, Nafeesah Haider, Kate Haigh, Victoria Haile, Jessica Hailstone, Christina Haines, Scott Hainey, Morton Hair, Brigid Hairsine, Juraj Hajnik, Danielle Hake, Lukman Hakeem, Anne Haldeos, Writaja Halder, Emily Hale, Jennie Hale, Carmel Halevy, Paul Halford, William Halford, Alaina Halim, Alistair Hall, Anthony Hall, Claire Hall, Elizabeth Hall, Fiona Hall, Helen Hall, Jennifer Hall, Kathryn Hall, Laura Hall, Jan Hallas, Kyle Hallas, Charles Hallett, Jackie Halliday, Akmal Hallman, Heather Halls, Maryam Hamdollah-Zadeh, ifedadepo A Hamed-Adekale, Bilal Hameed, Mohammed Hameed, Raph Hamers, Imran Hamid, Mohamad Hamie, Raymond Hamill, Bethany Hamilton, Fergus Hamilton, Gus Hamilton, Leigh Hamilton, Melanie Hamilton, Nicola Hamilton, Sara Hamilton, Ruth Hamlin, Eleanor Hamlyn, Beatrice Hammans, Shirley Hammersley, Kate Hammerton, Bev Hammond, Emily Hammond, Leah Hammond, Sally Hammond, Fiona Hammonds, Ibrahim Hamoodi, Karen Hampshire, James A Hampson, Jude Hampson, Lucy Hampson, Lisa Hamzah, Jenny Han, Ozan Hanci, Sadiyah Hand, Lala Handayani, Jasmine Handford, Soran Handrean, Nina K Handzewniak, Sarah Haney, Sheharyar Hanif, E Hanison, Esther Hanison, Jennifer Hannah, Amy Hannington, Merhej Hannun, Aidan Hanrath, Helena Hanratty, Daniel Hansen, Anita Hanson, Ashley Hanson, Helen Hanson, Jane Hanson, Kathryn Hanson, Steve Hanson, Mazhar Ul Haq, Ala Haqiqi, Monjurul Haque, Henry Harcourt, Lesley Harden, Zoe Harding, Simon Hardman, Marc Hardwick, Gareth Hardy, Joanna Hardy, Kumar Haresh, Rachel Harford, Beverley Hargadon, James Hargraves, Carolyn Hargreaves, Alice Harin, Mohammed Haris, Edward Harlock, Paula Harman, Tracy Harman, Mark Harmer, Muhammad A Haroon, Charlie Harper, Heather Harper, James Harper, Peter Harper, Rosemary Harper, Sarah Harrhy, Kate Harrington, Sian Harrington, Yasmin Harrington-Davies, Jade Harris, Jess Harris, John Harris, Laura Harris, Marie-Clare Harris, Nichola Harris, Sophie Harris, David Harrison, Laura Harrison, Melanie Harrison, Olakunbi A Harrison, Rachael Harrison, Rowan Harrison, Susie Harrison, Thomas Harrison, Wendy Harrison, Elizabeth Harrod, Ciaran Hart, Dominic Hart, Jessica Hartley, Lisa Hartley, Rosemary Hartley, Ruth Hartley, Tom Hartley, William Hartrey, Phillipa Hartridge, Stuart Hartshorn, Alice Harvey, Angela Harvey, Anna Harvey, Max Harvey, Catherine Harwood, Helen Harwood, Zoe Harzeli, Brigitte Haselden, K Hashem, Mohammed Hashimm, Tadaaki Hashimoto, Imranullah Hashmi, Jack Haslam, Zena Haslam, Grace Hasnip, Adil Hassan, Ali Hassan, Amro Hassan, Awab Hassan, Zeinab Hassan, Sapna Hassasing, Jane Hassell, Philip Hassell, Alex Hastings, Bethany Hastings, Janice Hastings, Sarah Hathaway-Lees, Jonathan Hatton, Jennifer Hau, May Havinden-Williams, Stefan Havlik, Daniel B Hawcutt, Kadean Hawes, Liz Hawes, Nicola Hawes, Lydia Hawker, Annie Hawkins, Joe Hawkins, Nancy Hawkins, W John Hawkins, Dan Hawley, Ed Hawley-Jones, Edward Haworth, Alasdair W Hay, Cathy Hay, Amna Hayat, Jamal Hayat, Mohamed-Riyal Hayathu, Anne Hayes, Jonas Hayes, Kate Hayes, Melony Hayes, Fiona Hayes, Patrick Hayle, Chloe Haylett, Antara Hayman, Melanie Hayman, Matthew Haynes, Richard Haynes, Rachel Hayre, Carole Hays, Sarah Haysom, James Hayward, Patrick Haywood, Tracy Hazelton, Phoebe Hazenberg, Zhengmai He, Elizabeth Headon, Carrie Heal, Brendan Healy, Jessica L Healy, Amy Hearn, David Heasman, Angela Heath, Debbie Heath, Rowan Heath, Diane Heaton, Adam Heavens, Kerry Hebbron, Catherine Heckman, Gemma Hector, Sadie Heddon, Andy Hedges, Katrine Hedges, Cheryl Heeley, Elaine Heeney, Richard Heinink, Rajdeep Heire, Ingvild Helgesen, Joanne Hemingway, Ulla Hemmila, Beth Hemmings, Scott Hemphill, Deborah Hemsley, Abigail Henderson, Eilidh Henderson, Jennifer Henderson, Steven Henderson, Joanne Henry, Karol Henry, Lavinia Henry, Margo Henry, Natalie Henry, David Henshall, Gillian Herdman, Rosaleen Herdman-Grant, Morag Herkes, Fe Marie Hernandez, Emma Heron, Lisa Heron, William Herrington, Emilia Heselden, Peta Heslop, Tracey Heslop, Simon Hester, Emily Hetherington, Joseph Hetherington, Chamila Hettiarachchi, Pramodh Hettiarachchi, Hayley Hewer, John Hewertson, Anna Hewetson, Sue Hewins, Nicola Hewitson, Claire Hewitt, Davina Hewitt, Richard Hewitt, Samuel Hey, Robert S Heyderman, Mathis Heydtmann, Joseph Heys, Jonathan Heywood, Meg Hibbert, John Hickey, Naomi Hickey, Peter Hickey, Alex Hicks, Jenny Hicks, Scott R Hicks, Daniel Higbee, Lucy Higgins, Andrew Higham, Martin Highcock, Judith Highgate, Mondy Hikmat, Amanda Hill, Helen Hill, Joanne Hill, Lisa Hill, Lucy Hill, Phoebe Hill, Uta Hill, Annette Hilldrith, Catherine Hillman-Cooper, Jon Hilton, Zoe Hilton, Sarah Hinch, Andrew Hindle, Esther Hindley, Alice Hindmarsh, Paul Hine, Kim Hinshaw, Clare Hird, Claire Hirst, Lindsay Hirst, Jemma Hives, Htet Myat Hlaing, Benson Ho, David K K Ho, Rosemary Ho, Michaela Hoare, David Hobden, Gill Hobden, Maria Hobrok, Simon Hobson, Christopher Hodge, Simon Hodge, Lesley Hodgen, George Hodgetts, Holly Hodgkins, Sally Hodgkinson, David Hodgson, Helen Hodgson, Luke Hodgson, Sheila Hodgson, Gemma Hodkinson, Kenneth Hodson, Mike Hodson, Alison Hogan, Matthew Hogben, Lucy Hogg, Lee Hoggett, Abigail Holborow, Catherine Holbrook, Rebecca Holbrook, Catherine Holden, Jill Holden, Melinda Holden, Sophie Holden, Thomas Holder, Niels Holdhof, Hannah Holdsworth, Lisa Holland, Maureen Holland, Nicky Holland, Poppy Holland, Susan Holland, Marie L Hollands, Elizabeth Holliday, Kyra Holliday, Mark Holliday, Nina Holling, Laszlo Hollos, Nora Hollos, Leah Holloway, Simon Holloway, Max Hollowday, Marcus Hollyer, Amy Holman, Ann Holmes, Megan Holmes, Raphael Holmes, Rebecca Holmes, Kelly Holroyd, Beth Holroyd-Hind, Lyndsey Holt, Siobhan Holt, Susie Holt, Alexandra Holyome, Marie Home, Renate Homewood, Kate Hong, Louise Hoole, Clare Hooper, Samantha Hope, Susan Hope, Bridget Hopkins, Peter W Horby, Stephanie Horler, Anil Hormis, Daniel Hornan, Nicola Hornby, Thomas Horne, Zoey Horne, Rebecca Horner, Caroline Horrobin, Latoya Horsford, Megan Horsford, Mark Horsford, Valana Horsham, Alexander Horsley, Ashley Horsley, Elizabeth Horsley, Sarah Horton, Jane Hosea, Toby Hoskins, Muhammad S Hossain, Rashed Hossain, Maxine Hough, Sarah Hough, Catherine Houghton, Kathryn Houghton, Rebecca Houlihan, Kay Housely, Hamish Houston, Roseanna Hovvels, Lee How, Laura Howaniec, Joanne Howard, Kate Howard, Laura Howard, Linda Howard, Lucy Howard, Megan Howard, Sarah Howard, Stuart Howard, Richard Howard-Griffin, Louise Howard-Sandy, Serena Howe, Alice Howell, Mark Howells, Lyn Howie, Kerry Howlett, Sophie Howlett, Ruth Howman, Josh Hrycaiczuk, Hein Htet, Naing Z Htoon, Su Htwe, Ying Hu, Chiang O H Huah, Sylvia Huang, Kelly Hubbard, Abby Huckle, Shahzya Huda, Alison Hudak, Lisa Hudig, Heather Hudson, Suzanne Hudson, Oli Hudson, Alison Hufton, Connor Huggins, Alistair Hughes, Caitlin Hughes, Emma Hughes, Gareth Hughes, Heather Hughes, Luke Hughes, Martin Hughes, Rachel Hughes, Rebecca Hughes, Samantha Hughes, Stephen Hughes, Vikki Hughes, Wesley Hughes, Lukas Huhn, Ching Hui, Ruth Hulbert, Diana Hull, Grace Hull, Robert Hull, Amanda Hulme, Peter Hulme, Wendy Hulse, George Hulston, Ryan Hum, Megan Hume, Charlotte Humphrey, Alasdair Humphries, Amy Humphries, Joanne Humphries, Charlotte Hunt, Fiona Hunt, Jane Hunt, Jonquil Hunt, Kristen Hunt, Luke Hunt, Molly Hunt, Sophie Hunt, Alexandra Hunter, Catherine Hunter, Ewan Hunter, Karl Hunter, Neil Hunter, Ross Hunter, Sophie Hunter, George Huntington, Fuad Huq, Elizabeth Hurditch, Judith Hurdman, Cian Hurley, Katrina Hurley, Mohammed A Husain, Syeda Y Husaini, Coralie Huson, Afreen Hussain, Chreif Hussain, Ibraar Hussain, Ifza Hussain, Mohammad Hussain, Mohammed Hussain, Muhammad Hussain, Reda Hussain, Rizwana Hussain, Samia Hussain, Sanniah Hussain, Yasmin Hussain, Mohammed Hussam El-Din, Samahir F E M Hussein, Ziad Hussein, Rebecca Hussey, Adiel H Hussien, Anja Hutchinson, Camille Hutchinson, Dorothy Hutchinson, Elizabeth Hutchinson, John Hutchinson, Claire Hutsby, Paula Hutton, Thuong Huyen, Hieu Huynh, Daniella Hydes, Jamie Hyde-Wyatt, Niamh Hynes, Megan Hyslop, Angeliki Iakovou, Katherine Ibison, Mazen Ibraheim, Abdalla Ibrahim, Ahmed Ibrahim, Asil Ibrahim, Javed Ibrahim, Mamoun Ibrahim, Mohamed Ibrahim, Wadah Ibrahim, Becky Icke, Adetokunbo I Idowu, Muhammad Idrees, Nauman Idrees, Hina Iftikhar, Mawara Iftikhar, Chukwuemeka Igwe, Ogechukwu Igwe, Mohammad Ijaz, Amaju Ikomi, Clare Iles, Stamatina Iliodromiti, Mary Ilsley, Lorna Ilves, Fatima M Ilyas, La'ali Imam-Gutierrez, Mohamed Iman, Christopher Imray, Haider Imtiaz, Jack Ingham, Jo Ingham, Julie Ingham, Rory Ingham, Tejas Ingle, Jennifer Inglis, Samantha Inglis-Hawkes, Anne Ingram, Luke Ingram, Tanya Ingram, Nicholas Innes, Peter Inns, Vithusha Inpadhas, Ken Inweregbu, Andreea A Ionescu, Ana Ionita, Ilian P Iordanov, Anil Ipe, Javaid Iqbal, Madiha Iqbal, Mishal Iqbal, Mohammed Iqbal, Faisal Iqbal Sait, Iziegbe Irabor, Jane Ireland, Jamie Irisari, Robert Irons, Mohannad Irshad, Muhammad S Irshad, Janice Irvine, Val Irvine, Robert Irving, Mina Ishak, Erica Isherwood, George Isitt, Aminul Islam, Tariqul Islam, Samsul Islam, Abdurrahman Islim, Ali Ismail, Omar Ismail, Caroline Ison, M'hamedi Israa, Sharon Isralls, Herny Istiqomah, Monica Ivan, Chineze Ivenso, Nicholas Ivin, Ashleigh Ivy, Sophie Iwanikiw, Karen Ixer, Menaka Iyer, Mia Iyer, Kashif Jabbar, Calum Jack, Joanne Jackman, Sophie Jackman, Amanda Jackson, Ben Jackson, Beth Jackson, Ella Jackson, Helen Jackson, Lauren Jackson, Melanie Jackson, Nicola Jackson, Shane Jackson, Yvonne Jackson, John Jacob, Patricia Jacob, Reni Jacob, Nicola Jacques, Anisa Jafar, Daniel Jafferji, Ali Jaffery, Chandrashekar Jagadish, Vijay Jagannathan, Mandeep Jagpal, Neemisha Jain, Seema Jain, Susan Jain, Swati Jain, Sanjay Jaiswal, Danyal Jajbhay, Thomas Jaki, Bintou Jallow, Yusuf Jaly, Rose Jama, Ashraf Jamal, Sabine Jamal, Zeba Jamal, Yasmin Jameel, Albie James, Christie James, Claire James, Kate James, Lee James, Linda James, Lorraine James, Mark James, Monica James, Nicholas James, Olivia James, Patricia James, Rebecca James, Ruth James, Sharon James, Somerset James, Tracy James, Jack Jameson, Louise Jamieson, Aaron Jamison, Phoebe Jane, Karen Janes, Azara Janmohamed, Deepa Japp, Peter Jaques, Victor Jardim, Catherine Jardine, Claire Jarman, Emma Jarnell, Ellie Jarvie, Claire Jarvis, Rosina Jarvis, Patrycja Jastrzebska, Hafsa Javed, Aristotle Javier, Mays Jawad, Lona Jawaheer, Anu Jayachandran, D Jayachandran, Anita Jayadev, Angelina Jayakumar, Deepak Jayaram, Ravi Jayaram, Geeshath Jayasekera, Thilina Jayatilleke, Abi Jayebalan, Jane Jeater, Saman Jeddi, Vandana Jeebun, Mohammad S Jeelani, Katie Jeffery, Helen Jeffrey, Rachel Jeffrey, Nathan Jeffreys, Benjamin Jeffs, Carol Jeffs, Jegan P Jeganathan Ponraj, Debbie Jegede, Taylor Jemima, Ifan Jenkin, Alison Jenkins, Christopher Jenkins, David Jenkins, Elinor Jenkins, Ian Jenkins, Paula Jenkins, Sarah Jenkins, Sian Jenkins, Stephen Jenkins, Felicia Jennings, Jacqui Jennings, Louise Jennings, Virginia Jennings, Ellen Jerome, Douglas Jerry, Greame Jervis, Ellen Jessup-Dunton, Jorge A Jesus Silva, Champa Jetha, Kishan Jethwa, Roshan K Jha, Shaman Jhanji, Khoo Jian, Zhixin Jiao, Laura Jimenez, Ana Jimenez Gil, Jithin Jith, Teishel Joefield, Navraj Johal, Simran Johal, Karine Johannessen, Aisyah Johari, Annie John, Anu John, Maya John, Navin John, Emma Johns, Margaret Johns, Antoinette Johnson, Emma Johnson, Gillian Johnson, Kathryn Johnson, Katie Johnson, Luke Johnson, Mark Johnson, Nelsonseelan Johnson, Oliver Johnson, Rachel Johnson, Brogan Johnston, Claire Johnston, Janet Johnston, Susan Johnston, Victoria Johnston, Dawn Johnstone, Ed Johnstone, Janet Johnstone, Manohar Joishy, Adam Jones, Alistair Jones, Amy Jones, Angela Jones, Annabel Jones, Ben Jones, Bryony Jones, Carys Jones, Ceri Jones, Charlotte Jones, Christine E Jones, Debra Jones, Emily Jones, Gareth Jones, Geraldine Jones, Jac Jones, James Jones, Jonathon Jones, Julie Jones, Kate E Jones, Kimberly Jones, Laura Jones, Laura M Jones, Louise Jones, Mathew Jones, Nancy Jones, Nicola Jones, Olivia Jones, Paul Jones, Philip H Jones, Rhianna Jones, Ruth E Jones, Samantha Jones, Sophie Jones, Stefanie Jones, Steve Jones, Susan Jones, Taya Jones, Thomas Jones, Tim Jones, Tracey Jones, Ramya Jonnalagadda, Rebecca Jordache, Michele Jordan, Sarah Jordan, Abi Jose, Litty Jose, Louise Jose, Sanal Jose, Anna Joseph, Gigee Joseph, P Aiden Joseph, Rosane Joseph, Sibet Joseph, Dhaara Joshi, Mehul Joshi, Pratichi Joshi, Tushar Joshi, Benz Josiah, Lijo Joy, Tiffany Joyce, Hong Ju, Adriel Ju Wen Kwek, Andrew Judd, Edward Jude, Parminder Judge, Jessica Juhl, Sirisha Jujjavarapu, Mark Juniper, Edmund Juszczak, Deepthi Jyothish, Kasamu Kabiru Dawa, Mark Kacar, Nikhil Kadam, Nicole Kader, Ajai Kailey, Matthew Kain, Gail Kakoullis, Azad Kala Bhushan, Richard J K Kalayi, Roobala Kaliannan Periyasami, Efthymia Kallistrou, Thomas Kalmus Eliasz, Seika Kalsoom, Elisa Kam, John Kamara, Ajay Kamath, Prakash Kamath, Ravindra Kamath, Siddharth A Kamerkar, Nick Kametas, Musaiwale Kamfose, Charlotte Kamundi, Dharmesh Kanabar, Leia Kane, Osei Kankam, Thogulava Kannan, Abhinav Kant, Vikas Kapil, Ritoo Kapoor, Sonal Kapoor, S Kaprapina, Sourjya Kar, Janaka Kara, Elshad Karbasi, Shivaali Karelia, Rona Kark, Abhilasha Karkey, Soheilali Karmali, Viji Karunanithi, Nicholas Karunaratne, Natashja Kasianczuk, Anbhu Kasiappan Balasubramanian, Vidya Kasipandian, Rizwan Kassam, Janarth Kathirgamachelvam, Victoria Katsande, Kulbinder Kaul, Daljit Kaur, Dervinder Kaur, Jasmin Kaur, Jaspreet Kaur, Simran Kaur, Zunaira Kausar, Lisa Kavanagh, susan Kavanagh, Mohammad A Al Kawser, Andrea Kay, Julie Kay, Robert Kay, Sarah Kay, Jossy N Kayappurathu, Callum Kaye, Ahemd Kazeem, Min Ke, Timothy Keady, Rachel Kearns, Nichola Kearsley, Joanne Keating, John Keating, Liza Keating, Elizabeth Keddie-Gray, Breffni Keegan, Rachel Keen, Natalie Keenan, Jonathan Kefas, Stephen Kegg, Laura Keith, Uzoamaka Keke, Joanne Kellett, Jo Kelliher, Alison Kelly, David Kelly, Diane Kelly, Dominic Kelly, Emma Kelly, Hannah Kelly, Laura Kelly, Louisa Kelly, Martin Kelly, Michael Kelly, Rosalind Kelly, Sinead Kelly, Stephanie Kelly, Stephen Kelly, Mary Kelly-Baxter, Olivia Kelsall, Marketa Keltos, Timothy Kemp, Emma Kendall, Alexandra Kendall-Smith, Sarah Kennard, Ann Kennedy, James Kennedy, Melanie Kennedy, Sophie Kennedy-Hay, Julia Kenny, Melanie Kent, Lynne Keogan, Alexander Keough, Dawn Kernaghan, A Kerr, Andrew Kerr, Caroline Kerrison, Anthony Kerry, Helen Kerslake, Ian Kerslake, Helen Kerss, Jocelyn Keshet-Price, Evelyne Kestelyn, Georgina Keyte, Abdul Khadar, Ali Khalid, Hadika Khalid, Muhammad U Khalid, Syed Khalid, Mariha Khalid, Tarek Khalifa, Amir Khalil, Asma Khalil, Sijjad Khalil, Abubakar Khan, Ali Khan, Al-Imran Khan, Arham Khan, Asad Khan, Aurangzeb Khan, Burhan Khan, Fatimah Khan, Kausik Khan, Malik A Khan, Marria Khan, Mehrunnisha Khan, Mohammad Khan, Myra Khan, Nayeem Khan, Nuzhath Khan, Omar Khan, Rabia Khan, Rahila Khan, Shabana Khan, Shahul Khan, Shoaib Khan, Tasaduksultan Khan, Waleed Khan, Waseem Khan, Zuhair Khan, Mohammad S Khan Tharin, Usman F Khatana, Januka Khatri, Jibran Khatri, Jyoti Khatri, Hafiza Khatun, Taslima Khatun, Mena Kheia, Jacyntha Khera, Htet Htet Ei Khin, Najaf Khoja, Kiran Khokhar, Joshua Khoo, Chloe Khurana, Joyce Kibaru, Faith Kibutu, Andrew Kidd, Michelle Kidd, Joe Kidney, Shane Kidney, Will Kieffer, James Kilbane, Caroline Kilby, Eliz Kilich, Eileen Killen, Beverley Kilner, Susan Kilroy, Bomee Kim, Jee Whang Kim, Minhee Kim, Aly Kimber, Sarah Kimber, Andy King, Barbara King, Hayley King, Jennifer King, Jenny King, Kirsten King, Matthew King, Rachel King, Sarah King, Victoria King, Emily King-Oakley, Laura Kingsmore, Deborah J Kinnear, Fiona Kinney, Sidra Kiran, Alison Kirby, Adam Kirk, Jeremy Kirk, Jill Kirk, Jodie Kirk, Amy Kirkby, Emily Kirkham, Gemma Kirkman, Lucy Kirkpatrick, Ursula Kirwan, Toby Kitching, Laura Kitto, Lauren Kittridge, Sarah Klaczek, Frieder Kleemann, Susan Kmachia, Christopher P Knapp, Lucy Knibbs, Alicia Knight, Fraser Knight, Marian Knight, Sarah Knight, Steven Knight, Tom Knight, Ellen Knights, Jane Knights, Martin Knolle, Paul Knopp, Charlotte Knowles, Karen Knowles, Laurence Knowles, Emily Knox, Lucy Knox, Oliver Koch, Marta Kocsor, Ronan Kodituwakku, Gouri Koduri, Yuan Jun Koe, Aisha Koirata, Eirene Kolakaluri, Magdalena Kolodziej, Eirini Kolokouri, Samantha Kon, Niladri Konar, Mari Kononen, Athanasios Konstantinidis, Rachel Kontogonis, Hui Fen Koo, Imogen Koopmans, Emmanuela Kopyj, Laura Korcierz, James Korolewicz, George Koshy, Chris Kosmidis, Csilla Kosztolanyi, Jalpa Kotecha, Easwari Kothandaraman, Katerina Koukou, Athanasia Kountourgioti, Koushan Kouranloo, Rukhsana Kousar, Margarita Kousteni, Anastasia Koutalopoula, Maja Kovac, Alex Kozak Eskenazia, Kestutis Krasauskas, Raghu Krishnamurthy, Vinodh Krishnamurthy, Manju Krishnan, Hari Krishnan, Nagaratna Krishnapalli, Suzanne Krizak, Stephen Krueper, Sean Krupej, James Krzowski, Ria Kubaisi, Agnieszka Kubisz-Pudelko, Shavita Kuckreja, Soren Kudsk-Iversen, Aurimas Kudzinskas, Chirag Kukadiya, Nainesha Kulkarni, Susantika Kumala Dewi, Aditi Kumar, Ayesha Kumar, Mayur Kumar, Ramesh Kumar, Ravi Kumar, Rita Kumar, Rupa Kumar, Satish Kumar, Vikas Kumar, Vimal Kumar, Arun Kundu, Heinke Kunst, Klaudia Kupiec, Amit Kurani, Mohammed Kurdy, Kevin Kuriakose, Rincy Kurian, Vimal Kurmars, Cameron Kuronen-Stewart, Ranganai S Kusangaya, Vlad Kushakovsky, Anna Kutera, Apexa Kuverji, Amma Kyei-Mensah, Thyra Kyere-Diabour, Moe Kyi, Nyan M Kyi, Laura Kyle, Karali-Tsilimpari Kyriaki, Julius Labao, Louise Lacey, Nikki Lack, Maria Lacson, Zahra Ladan, Emma Ladlow, Heather Lafferty, Alice Lagnado, Shondipon Laha, Sushil Lahane, Clement Lai, James Lai, Paula Laidler, Robert Laing, Inez Laing-Faiers, Emily Laity, Katharine Lake, Nicki Lakeman, David Lalloo, Fiona Lalloo, Alison Lam, Classy Lam, Fiona Lamb, Lucy Lamb, Thomas Lamb, Pauline Lambert, Claudia Lameirinhas, Mohammed K G Lami, Holly Lamont, Michal Lamparski, Djillali Lamrani, Christine Lanaghan, Ivone Lancona-Malcolm, Geraldine Landers, Martin J Landray, Matthew Lane, Nicholas Lane, Alidih Lang, Stephen Lang, Daniel Langer, Margaret Langley, Charles Langoya, Emma Langridge, Emily Langthorne, Helen Langton, Beatriz Lara, Taiya Large, Samuel Lassa, Anna Last, Scott Latham, Victoria Latham, Afzal Latheef, Larissa Latif, Nang Latt, Carmen Lau, Dawn Lau, Eva Lau, Gonzalez G Laura, Myra Laurenson, Eloise Lavington, Hou Law, Jessica Law, June Law, Kar Yee Law, Penny Law, Richard Law, Laura Lawless, Chris Lawrence, Emma Lawrence, Geoffrey Lawrence, Holly M Lawrence, Neil Lawrence, Ryan Lawrie, Louise Lawson, Natalie Lawson, Rod Lawson, Michael Lay, Stephen Laybourne, Christine Laycock, Reina Layug, Maria Lazo, Vietland Le, Amelia Lea, William Lea, Louisa E Leach, Ian Leadbitter, Thomas Leahy, Richard Lean, Lorna Leandro, Darren Leaning, Ruth Leary, Sandra Leason, Marie Anne Ledingham, Caroline Lee, Emma Lee, Hannah Lee, Irish Lee, Joshua Lee, Judith Lee, Sam Lee, Shannon Lee, Shi Han Lee, Simon Lee, Sindy Lee, Stephanie Lee, Tracey Lee, Xiang Lee, Robyn Lee, Diana Lees, Jennifer Lees, Helen Legge, Julian Leggett, Katie Leigh-Ellis, David Leitch, Nicky Leitch, Eleni Lekoudis, Petula Lemessy, Nicholas Lemoine, Rebecca Lenagh, Katy Leng, Katrina Lennon, Liz Lennon, Beth Leonard, Kelly Leonard, Wen Leong, Nicky Leopold, Oskar Lepiarczyk, Isla Leslie, Eleni Lester, Emma Levell, Chris Levett, Anastasia Levynska, Alice Lewin, Alison Lewis, Christopher Lewis, David Lewis, Dee Lewis, Heather Lewis, Joanne Lewis, Joseph Lewis, Kathryn Lewis, Keir Lewis, Leon Lewis, Mandy Lewis, Marissa Lewis, Nicolas Lewis, Robert Lewis, Catherine Lewis-Clarke, Adam Lewszuk, Penny Lewthwaite, Samantha Ley, Angela Liao, Victoria Licence, David Lieberman, Susan Liebeschuetz, Teresa Light, Nicky Lightfoot, Patrick Lillie, Ashling Lillis, Ben Lim, Carys Lim, Ee Thong Lim, Ivy Lim, Terence Lim, Wei Shen Lim, Wilson Lim, James Limb, Dipraj Limbu, Usha Limbu, Christian Linares, Dermot Linden, Gabriella Lindergard, Kate Lindley, Charlotte Lindsay, Emily Lindsay, Max Lindsay, Helen Lindsay- Clarke, Mirella Ling, Claire Lingam, Linette Linkson, Thinzar Linn, Mike Linney, Conrad Lippold, George Lipscomb, Karen Lipscomb, Laura Lipskis, Ana Lisboa, Evangeline Lister, Jeff Little, Sam Little, Lucy Littlejohn, Sky Liu, Xuedi Liu, Daniel K Llanera, Rhiannon Llewellyn, Martin Llewelyn, Adam Lloyd, Aimee Lloyd, Arwel Lloyd, Oliver Lloyd, Richard Lloyd, Su Lo, David Loader, Caroline Loan, Livia Lobosco, Lydianne Lock, Sara Lock, Stephen Lock, Angela Locke, Jacqueline Locke, Thomas Locke, Jacqueline Locke, Teresa Lockett, Jeorghino Lodge, Krishan Lodhia, Martina Lofthouse, Heather Loftus, Meg Logan, Chloe Alice Logue, Sook Yin Loh, Siddharth Lokanathan, Kaatje Lomme, Emily London, Gabriella Long, Natalie Long, Katherine Longbottom, Bev Longhurst, Mark Longshaw, Sarah Longstaffe, Jennifer Lonnen, Caroline Lonsdale, Laura Looby, Ronda Loosley, Liliana Lopes, Paola Lopez, Paula Lopez, Robert W Lord, Stephen Lord, Claire Lorimer, Francesco Loro, Rachel Lorusso, Catherine Loughlin, Wayne Lovegrove, Robert Loveless, Maxine Lovell, Angeliki Loverdou, Andrew Low, Jen Mae Low, Shona Low, Alastair Lowe, Catherine Lowe, Emily Lowe, Faye Lowe, Leanne Lowe, Michael Lowe, Richard Lowsby, Vicki Lowthorpe, Gamu Lubimbi, Alexandra Lubina Solomon, Georgia Lucas, Jacob Lucas, Alice Lucey, Olivia Lucey, Suzanne Luck, Lok Hin Lui, Akish Luintel, H Luke, Jane Luke, Naomi Lungu, Apurva Lunia, Muriel Lunn, Ji Luo, Mark Luscombe, Jocelyn Luveta, Cindy N Luximon, Khine Lwin, Myat Lwin, Alison Lye, Barrie Lyell, Elisavet Lyka, Audrey Lynas, Ceri Lynch, Daniel Lynch, Daniella Lynch, Stephen Lynch, Rea-Grace Maamari, Hannah Mabb, Louies Mabelin, George Mabeza, Jessica Macaro, Kateryna Macconaill, Chloe Macdonald, Andrew Macduff, Claire Macfadyen, James G Macfarlane, Jill Macfarlane, Laura Macfarlane, Irene Macharia, Lisa MacInnes, Iain MacIntyre, Jill MacIntyre, Kirsten Mack, Callum Mackay, Euan Mackay, Laura Mackay, Alexander Mackenzie, Matt Mackenzie, Robert MacKenzie Ross, Ami Mackey, Fiona Mackie, Jennifer Mackie, Robert Mackie, Carolyn Mackinlay, Claire Mackintosh, Katherine Mackintosh, Mary J MacLeod, Shona Macleod, Michael Macmahon, Andrew MacNair, Catherine Macphee, Iain Macpherson, Catriona Macrae, Allan MacRaild, Alannah Madden, Mary Madden, Christopher Madden-McKee, Sally Maddison, Norman Madeja, Pradeep Madhivathanan, Madhavi Madhusudhana, Alpha Madu, Lorraine Madziva, Marion Mafham, Nick Magee, Frederick Magezi, Negar Maghsoodi, Christopher Magier, Lessica M Magnaye, Marios Magriplis, Michelle Magtalas, Natasha Mahabir, Subramanian Mahadevan-Bava, Amitaa Maharajh, Anjanie Maharajh, Kijan Maharjan, Ajit Mahaveer, Bal Mahay, Kanta Mahay, Amy Mahdi, Hibo Mahdi, Noor Mahdi, Thushika Mahendiran, Siva Mahendran, Sarah Maher, Anistta Maheswaran, Shameera Maheswaran, Tina Maheswaran, Parisa Mahjoob-Afag, Ahmed Mahmood, Farhana Mahmood, Hussain Mahmood, Waheed Mahmood, Zahra Mahmood, Hager Mahmoud, Mohammed Mahmud, Ewan Mahony, Tabitha Mahungu, Luke Mair, Toluwani Majekdunmi, Kesson Majid, Amy Major, Rupert Major, Jaydip Majumdar, Mohammad K H Majumder, Tsz L A Mak, Annabel Makan, Esther Makanju, Stephen Makin, Wura-Ola Makinde, Yahya Makkeyah, Marius Malanca, Hannah Malcolm, Flora Malein, Neeraj Malhan, Agnieszka Malicka, Ayesha Malik, Gulshan Malik, Mohammed Maljk, Paul Mallett, Petrina Mallinder, Georgia Mallison, Louise Mallon, Edward Malone, Gracie Maloney, Madhu Mamman, Irene Man, Kathy Man, Rossana Mancinelli, Marco Mancuso-Marcello, Shrawan K Mandal, Sudeshna Mandal, Tracy Manders, Lauren Manderson, Justin Mandeville, Tara Mane, Roope Manhas, Carmen Maniero, Ravi Manikonda, Ilham Manjra, Rowan Mankiewitz, Bobby Mann, Jonathan Manning, Sarah Manning, Pascoe Mannion, Katherine Mansi, Katarina Manso, Dina Mansour, Mohamed Mansour, Ramy Mansour, Isheunesu T Mapfunde, Predeesh Mappa, Arrusaw Maqsood, Hemant Maraj, Clare Marchand, Neil Marcus, Anna Marcyniuk, Maria Marecka, Deborah Maren, Gomathi Margabanthu, Jordi Margalef, Lavinia Margarit, Georgios Margaritopoulos, Mike Margarson, Fernandez Maria del Rocio, Teresa Maria Pfyl, Victor Mariano, Ashleigh Maric, Grace Markham, Ben Marks, Maria Marks, P Marks, Emily Marler, Elisabeth Marouzet, Arran Marriott, Cheryl Marriott, Nemonie Marriott, Christopher Marsden, Karen Marsden, Paul Marsden, Sarah Marsden, Tracy Marsden, Cathryn Marsh, Glen Marsh, Robyn Marsh, Adam Marshall, Andrew Marshall, Gail Marshall, Henry Marshall, Jaimie Marshall, Jenna Marshall, Nicola Marshall, Riley Marshall, Steven Marshall, Jennifer Marshall, Emmeline Martin, Gaynor Martin, Hayley Martin, Hope Martin, Jane Martin, Karen Martin, Kate Martin, Laila Martin, Martha Martin, Michael Martin, Noelia Martin, Tim Martin, Winston Martin, Sarah Martin, Tim Martindale, Marcus Martineau, Lauren Martinez, Jose C Martinez Garrido, Juan Martin-Lazaro, Vijay K Maruthamuthu, Bukhari Marwan, Gemma Maryan, Roman Mary-Genetu, Sam Maryosh, Vidan Masani, Allison Mascagni, Diego Maseda, Zandile Maseko, Sheila Mashate, Yasaman Mashhoudi, Al Mashta, Izhaq Masih, Sanna Masih, Nicholas Maskell, Perry Maskell, Matthew Masoli, Julia Mason, Rebecca Mason, Richard Mason, Ruth Mason, Claire Mason, Mohammad Masood, Mohammad T Masood, Syed S M E Masood, Thalia Massa, Ian Massey, Joseph Masters, Aaqib Masud, Lear Matapure, Cristina Matei, Ropafadzo Matewe, Manraj Matharu, Stephy Mathen, Alex Mather, Nicole Mather, Jonathan Mathers, Joanna Matheson, Amal Mathew, Anna Mathew, Bijoy Mathew, Moncy Mathew, Verghese Mathew, Jesha Mathews, Kate Mathias, Alexander Mathioudakis, Darwin Matila, Wadzanai Matimba-Mupaya, Nashaba Matin, Elina Matisa, Ellen Matkins, Max Matonhodze, Elijah Matovu, Jaysankar Mattappillil, Alison J Matthews, Clive Matthews, Heather Matthews, Helen Matthews, Lehentha Mattocks, Charlotte Maughan, Emma Mawson, Fiona Maxton, Adam Maxwell, Veronica Maxwell, Emily May, James May, Joanne May, Philippa May, Irving Mayanagao, Matthew Maycock, Jordan Mayer, Graham Mayers, Victoria A Maynard, Thomas Mayo, Lioniza Mayola, Shelley Mayor, Ibreaheim Mazen, Thamani Mazhani, Andrea Mazzella, Nyambura Mburu, Angeline Mbuyisha, Celine Mc Cague, Eleanor McAleese, Paul McAlinden, Lesley McAllister, Audrey McAlpine, Graeme McAlpine, Jonathan McAndrew, Hamish McAuley, Sarah McAuliffe, Claire McBrearty, Erin McBride, Michael McBuigan, James McBurney, Laura McCabe, Gemma L McCafferty, Laura McCafferty, Amanda McCairn, Jake McCammon, Nicole McCammon, Conor McCann, Erin McCann, Alexandra McCarrick, Brendan McCarron, Eoghan McCarthy, Michelle McCarthy, Natalie McCarthy, Sinead McCaughey, Tara McClay, Beverley McClelland, Declan McClintock, Michael McCloskey, Kirsten McCollum, Alasdair McCorkindale, Patricia McCormack, Jacqueline McCormick, Jake McCormick, Wendy McCormick, Paul McCourt, Jame McCrae, Sharon McCready, Gordan McCreath, Helen McCreedy, Claire McCue, Iain J McCullagh, Liz McCullagh, Megan McCullagh, Conor McCullough, Katherine McCullough, Nicola McCullough, Sarah McCullough, Fiona McCurrach, John McDermott, Paula McDermott, Rory McDermott, Katharine McDevitt, Helen McDill, Basil McDonald, Carly McDonald, Claire McDonald, Debbie McDonald, Rob McDonald, Sam McDonald, Damhnaic McDonald, Natasha McDonnell, Catherine McDougall, Lilly McDougall, Rowan McDougall, Irene McEleavy, Fred McElwaine, Julie McEntee, Evanna McEvoy, Claire McEwan, Ruth McEwen, Margaret McFadden, Denise McFarland, Margaret McFarland, Rachel McFarland, Joe McFlynn, Erin McGarry, Lorcan McGarvey, Alex McGeachan, Frederick McGee, Laura McGenily, Clodagh McGettigan, Michael McGettrick, Christopher McGhee, Fiona McGill, Sarah McGinnity, Neil McGlinchey, Phil McGlone, Deborah McGlynn, Claire McGoldrick, Clare McGoldrick, Elizabeth McGough, Christopher McGovern, Roisin McGovern, Angela McGowan, Anne McGown, Brendan McGrath, Amanda McGregor, Annemarie McGregor, Michael P McGuigan, Heather McGuinness, Sean McGuire, Tara McHugh, Caroline McInnes, Neil McInnes, Jerome McIntosh, Karen McIntyre, Mhairi McIntyre, Lorna McKay, Conor P McKeag, Jacqueline McKeane, Madeleine McKee, Joseph McKeever, Judith McKenna, Shirley McKenna, Mary McKenzie, Donogh McKeogh, Caroline McKerr, Anthony M McKie, Hayley Mckie, Laura Mckie, Gerard McKnight, Heather McLachlan, Andrew McLaren, Barbara McLaren, Nicola McLarty, Danielle Mclaughlan, Maria McLaughlin, James McLay, Mary McLeish, Tina McLennan, Stewart McLure, Anne Marie McMahon, Genevieve McMahon, Mike McMahon, Stephen McMahon, Terence McManus, Moyra McMaster, Paddy McMaster, Faye Mcmeeken, Samuel McMeekin, Nicola McMillan, Katherine McMillen, Jason McMinn, Liam McMorrow, Heather McMullen, Christopher McMurran, Helen McNally, Fiona McNeela, Lynne McNeil, Claire McNeill, Jessica McNeill, Shea McNeill, Una McNelis, Melanie McNulty, Roisin McNulty, Christopher McParland, Mark McPhail, Alison McQueen, Anna McSkeane, Denise McSorland, Therese McSorley, Gini McTaggart, Jacqueline McTaggart, Joanna Mead, Paul Mead, Emma Meadows, Olivia Meakin, Ben Mearns, Claire Mearns, Kim Mears, William Mears, Manjula Meda, Ayren Mediana, Ross Medine, Thomas Medveczky, Sharon Meehan, Emily Meeks, Abbi Megan, Nevan Meghani, Salim Meghjee, Sharon Megson, Amina Mehar, Mehr N Mehmood, Rohan Mehra, James Meirill, James Meiring, Rayane Mejri, Ellen Mekonnen, Sabina Melander, Adriana-Stefania Melinte, Jennifer Mellersh, Lucy Melling, Christie Mellish, Francesca Mellor, Joe S Mellor, Samantha Mellor, Zoe Mellor, Katrina Mellows, Vladimir Melnic, Alice Melville, Dominique Melville, Julie Melville, Helen Membrey, Mark Mencias, Adam Mendelski, Cheryl Mendonca, Carron Meney, Winifred Mensah, Alexander Mentzer, Dan Menzies, Sarah Menzies, Sue Mepham, Oliver Mercer, Pauline Mercer, Arwa Merchant, Fatema Merchant, Mihaela Mercioniu, Megan Meredith, Marta Merida Morillas, Blair Merrick, Jack Merritt, Sharon Merritt, Simon Merritt, Paul-Peter Merron, Ekta Merwaha, Simon Message, Jenny Messenger, Gabriel Metcalf-Cuenca, Alexandra Metcalfe, Benjamin Metcalfe, Kneale Metcalfe, Stella Metherell, Alexsandra Metryka, Louise Mew, Simon Meyrick, Nhlanhla Mguni, Atiqa Miah, Jagrul Miah, Nahima Miah, Areeb Mian, Gabriela Mic, Dariush Micallef, Alice Michael, Angiy Michael, Shery Michael, Natalia Michalak, Loredana Michalca-Mason, Ola Michalec, Janet Middle, Hayley Middleton, Jennifer T Middleton, Maeve Middleton, Sophie Middleton, Shelley Mieres, Loredana Mihalca-Mason, Theresia Mikolasch, Sarah Milgate, Colin Millar, Jonathan Millar, James Millard, David Miller, Johnathan Miller, Lucy Miller, Rachel Miller, Naomi Miller-Biot, Alex Miller-Fik, Louise Millett, Barry Milligan, Hazel Milligan, Iain Milligan, Caitlin Milliken, Katherine Millington, Rhys Millington, Samuel Millington, Helen Mills, Janet Mills, Rebecca E Mills, Helen Millward, Rebecca Miln, Alice Milne, Charlotte Milne, Louise Milne, Joanne Milner, Leanne Milner, Zayar Min, Samuel Mindel, Chrissie Minnis, Paul Minnis, Konstantina Minou, Natalie Minskip, Jane Minton, Frederico Miranda, Mukaj Mirela, Taimur Mirza, Anjum Misbahuddin, Aseem Mishra, Biswa Mishra, Eleanor Mishra, Ritu Mishra, Sannidhya Misra, Deena Mistry, Heena Mistry, Dushyant Mital, Sarah Mitchard, Ben Mitchell, Caroline Mitchell, Luke J Mitchell, Piers Mitchell, Philip Mitchelmore, Andrew Mitra, Atideb Mitra, Sandip Mitra, Nomsa Mlambo, Emma Moakes, Kirsten Moar, Emma Moatt, Daniela Mock Font, Gita Modgil, Abdelrahman Mohamed, Arez Mohamed, Osab Mohamed, Akram Mohammad, Waheed Mohammad, Aliabdulla Mohammed, Omer Mohammed, Yaser N S Mohammed, Bilal A Mohamud, Amr Moharram, Hoi-Ping Mok, Jonathan Mok, Leslie Mokogwu, Marcelino Molina, Christine Moller-Christensen, Mateus Mollet, Malid Molloholli, Aoife Molloy, Linda Molloy, Andrew Molyneux, Rebekah Molyneux, Tasnim Momoniat, Holly Monaghan, Krista Monaghan, Shiva Mongolu, Tesha Monika, Katelyn Monsell, Mahmoud Montasser, Alan Montgomery, Hugh Montgomery, Prebashan Moodley, Margaret Moody, Nick Moody, Angela Moon, James Moon, Ji-Hye Moon, Maria Moon, May Moonan, Parvez Moondi, Suzanne Moorby, Jim Moorcroft, Alex Moore, Christopher Moore, David A J Moore, Faye Moore, Judith Moore, Laura Moore, Natalie Moore, Sally Moore, Siobhan Moore, Sonia Moore, Vanessa Moore, Rachel Moores, Ed Morab, Jose Morales, Nuria Moramorell, Louise Moran, Grishma Moray, Jeronimo Moreno-Cuesta, Alexander Morgan, Amy Morgan, Caitlin Morgan, Christine Morgan, Colin Morgan, Holly Morgan, Katie Morgan, Lauren Morgan, Leila Morgan, Lewis Morgan, Matthew Morgan, Patrick Morgan, Katie Morgan-Jones, Emily Morgan-Smith, Joseph Morilla, Anna Morley, Thomas Morley, Wendy Morley, Anna Morris, Damian Morris, Fiona Morris, Helen Morris, Juliet Morris, Katie Morris, Laura Morris, Lucy Morris, Mary-Anne Morris, Niall Morris, Paul Morris, Sheila Morris, Susan Morris, Douglas Morrison, Moira Morrison, Scott Morrison, Mary Morrissey, Abbie C Morrow, Anna Morrow, Franca Morselli, Gordon Mortem, Valerie Mortland, Chelsea Morton, Gordon Morton, Priti Morzaria, Daniel Mosby, Linda Moseley, Karyn Moshal, Ben Moshy, Alison Moss, Charlotte Moss, James Moss, Sarah Moss, Stuart Moss, Omar Mostafa, Georgia Moth, Nicki Motherwell, Sharon Mottershaw, Harmesh Moudgil, Johanna Mouland, Caroline Moulds, Hilary Moulton, Ginny Mounce, Elizabeth Mousley, Chris Mowatt, Karen Moxham, Borja Moya, Quberkani Moyo, Eunice Mshengu, Sheila Mtuwa, Ali Muazzam, Iqtedar A Muazzam, Nykki Muchenje, Dalia Mudawi, Girish Muddegowda, Rabia Mufti, Imran Mugal, Ahsan Mughal, Javaid Muglu, Fawad Muhammad, Javed Muhammad, Carol Muir, Aparna Mukherjee, Dipak Mukherjee, Jameel Mukhtar, Syed A A Mukhtar, Denise Mukimbiri, John Mulcahy, Michelle Mulcahy, Peter Mulgrew, Ben Mulhearn, Arafat Mulla, Dee Mullan, Dileepkumar Mullasseril Kutten, Niall Mullen, Rosemary Mullett, Ciara Mulligan, Sandra Mulligan, Lana Mumelj, Andrew Mumford, Mohammed Munavvar, Henry Munby, Hannah Munday, Anne-Marie Munro, Sheila Munt, McDonald Mupudzi, Arshid Murad, Oluwatosin H Muraina, Koteshwara Muralidhara, Mhairi Murdoch, Jennifer Murira, Alison Murphy, Ben Murphy, Carl Murphy, Cathal Murphy, Erin Murphy, Gail Murphy, Hannah Murphy, Peter Murphy, Rebecca Murphy, Sheenagh Murphy, Simon Murphy, Alison Murphy, Clare Murray, David Murray, Dawn Murray, Eleanor Murray, Katie Murray, Kenneth Murray, Lindy Murray, Lisa Murray, Lorna Murray, Tracey Murray, Eoin Murtagh, Mithun Murthy, Catherine Murton, Rosie Murton, Neeka Muru, Rosemary Musanhu, Maimuna Mushabe, Omaisa Mushtaq, Ahmed M M Mustafa, Elhaytham Mustafa, Mustafa Mustafa, Ibrahim Mustapha, Naveed Mustfa, Zhain Mustufvi, Callum Mutch, Rachel Mutch, Eric Mutema, Balakumar Muthukrishnan, Sheree Mutton, Natasha Muzengi, Memory Mwadeyi, Bettina Mwale, Esther Mwaura, Raji Myagerimath, Alice Myers, Sam Myers, James S Myerson, Khin Swe Myint, Yadee Myint, Georgina Mynott, Libor Myslivecek, Patricia Nabayego, Evelyn Nadar, Iftikhar Nadeem, Moosa Nadheem, Behzad Nadjm, Asma Naeem, Hassan Naeem, Salman Naeem, Samraiz Nafees, Mohamed Nafei, Thapas Nagarajan, Leah Naglik, Imrun Nagra, Deepak Nagra, Mina Naguib, Kirushthiga Naguleswaran, K Shonit Nagumantry, Kevin Naicker, Sarveshni Naidoo, Gireesha Naik, Rishi Naik, Samir Naik, Devu S Nair, Rajiv Nair, Tanushree Nair, Jay Naisbitt, Kerry Naismith, Deborah Nakiboneka-Ssenabulya, Sri Nallapareddy, Soum Nallapeta, Arumugan Nallasivan, Uttam Nanda, Aarti Nandani, Tara Nandwani, Ali Raza Naqvi, Asadullah Naqvi, Sara Naqvi, Sophia Nasa, Dominic Nash, Nader Nasheed, Abdul Nasimudeen, Umer Nasir, Tahir Nasser, Anuja Natarajan, Geetha Natarajan, Nalin Natarajan, Nikhila Natarajan, Rajkumar Natarajan, Preethy Nath, Noel Nathaniel, Mala Nathvani, Priyan Nathwani, George Nava, Neena Navaneetham, Jeya Navaratnam, Helen Navarra, Sadaf Naveed, John Navin, Khuteja Nawaz, Sarfaraz Nawaz, Shasta Nawaz, Bonilla Nayar, Suzanne Naylor, Moez Nayyar, Farrah Naz, Mobeena Naz, Babak Nazari, Abida Nazir, S Nazir, Sehar Nazir, Dumisani Ncomanzi, Onyinye Ndefo, Narcisse B Ndoumbe, Abigail Neal, Alan Neal, Elaine Neary, Mostafa Negmeldin, Jonathan Neil, Paula Neill, Hector E Neils, Avideah Nejad, Jeremy Nel, Louise Nel, Benjamin Nelson, Lauren Nelson, Marie Nelson, Memory Nelson, Richard Nelson, Scott Nelson, Sven Nelson, Erni Nelwan, Rajesh Nemane, Samiksha Nepal, Daniel Nethercott, Kimberley Netherton, Kimberley Nettleton, Alison Newby, Angela Newby, David Newby, Tracy Newcombe, Helen Newell, Charlotte Newman, Diana Newman, Hannah Newman, Julie Newman, Oscar Newman, Tabitha Newman, Thomas Newman, Rachel Newport, Maria Newton, Anthony Y K C Ng, Hui E J Ng, Ka Wing Ng, Maxine Ng, Sarah Ng, Wee Jin Ng, Yee W M Ng, Thomas Ngan, Gabriel CE Ngui, Alice Ngumo, Quang Nguyen, Keith Ngwenya, Caoimhe Nic Fhogartaigh, Nathalie Nicholas, Philip Nicholas, Rachel Nicholas, Rebecca Nicholas, Donna Nicholls, Lisa Nicholls, Sarah Nicholls, Alice Nicholson, Anne Nicholson, Annette Nicholson, Ian Nickson, Eileen Nicol, Elizabeth Nicol, Rebecca Nicol, Pantelis Nicola, Antony Nicoll, Tania Nightingale, Faria Nikita, Pantzaris Nikolaos, Georgii Nikonovich, Annette Nilsson, Kofi Nimako, Louise Nimako, Camus Nimmo, Preethy Ninan, Mahesh Nirmalan, Attiya Nisar, Muhammad Nisar, Tosia Nisar, Toby Nisbett, Aksinya Nisha James, Sabaahat Nishat, Tomoko Nishiyama, Sara Nix, Jennifer Nixon, Maxine Nixon, Khwaja Nizam Ud Din, Maria Nizami, Serafeim Nizamis, Raymond Njafuh, India Noakes, Lyrics Noba, Jennifer Noble, Jonathan Noble, Harriet Noble, Hsu M Noe, Jerry Nolan, Jackson Nolasco, Zahid Noor, Zaid Noori, Jayne Norcliffe, Louis Norman, Rachel Norman, Emma Norris, Karen Norris, Lillian Norris, Sally Ann Nortcliffe, Fiona North, Julie North, Thomas North, John Northfield, Samantha Northover, Jurgens Nortje, Donna Norton, Rowen Norton, Holly Notman, Khalid Nourein, Timea Novak, Nuria Novas Duarte, Catherine Novis, Justyna A Nowak, Khin Phwe Nu, Mohamed Nugdallah, Anne M Nugent, Justine Nugent, Numbere Numbere, Kribashnie Nundlall, Arvind Nune, Kieran Nunn, Michelle Nunn, Jane Nunnick, Yvonne Nupa, Fadumo Nur, Zubeir Nurgat, Amy Nuttall, Lorenza Nwafor, Paul C Nwajiugo, Godfrey Nyamugunduru, Maggie Nyirenda, Kerry Nyland, Donal O Rinn, Daire O Shea, Megan O Toole, Marianne O'Hara, Chloe O'Hara, Laura O'Keefe, Kevin O'Reilly, William O'Rourke, Caroline Oakley, Naomi Oakley, Susan Oakley, Begho Obale, Clements Oboh, Clare O'Brien, Julie O'Brien, Kirsty O'Brien, Linda O'Brien, Neale O'Brien, Rachel O'Brien, Tracey O'Brien, Emma O'Bryan, Ross Obukofe, Christopher O'Callaghan, Lorcan O'Connell, Tadg OConnor, Chris O'Connor, Grainne O'Connor, Miranda Odam, Sam Oddie, Sharon Oddy, Yejide Odedina, Krishma Odedra, Sven W Odelberg, Natasha Odell, Omolola Oderinde, Jessica Odone, Lynn O'Donohoe, Catherine O'Donovan, Isabel Odysseos-Beaumont, Stephen O'Farrell, Pamela Offord, Morgan O'Flaherty, Tanwa Ogbara, Catherine Ogilvie, Ciaran O'Gorman, Ibrahim Ogunjembola, Oluwatomilola Ogunkeye, Udeme Ohia, Shalini Ojha, Sushma Ojha, Sushma Ojhas, Ohiowele Ojo, Fiona O'kane, Mark O'Kane, Tolu Okeke, Eleanor OKell, Alicia Okines, Iheoma Okpala, Ernest Okpo, F Okpoko, Maryanne Okubanjo, Chiamaka Oladipo, Liel A Olaivar, Raphael Olaiya, Jacob Olatujoye, Tim Old, Gregory Oleszkiewicz, Annie Oliver, Catherine Oliver, Jesse Oliver, Lucinda Oliver, Martyn Oliver, Zoe Oliver, Nurudeen O Olokoto, Folusho Olonipile, Olumide Olufuwa, Olatomiwa Olukoya, Akinlolu Oluwole-Ojo, Laura O'Malley, Maryam Omar, Mohamad Omar, Zohra Omar, Nimca Omer, Eti Omoregie, Connaire O'Neill, Lauran O'Neill, Chon S Ong, Onyinye Onuoha, Chidera Onyeagor, Chan N Oo, Zin N Oo, Huah C Ooi, Sze H Ooi, Amin Oomatia, Maria Opena, Richard Oram, Christy Ord, Jonathan Ord, Clare Oreilly, Lola Orekoya, Devaki O'Riordan, Sean O'Riordan, Izabela Orlikowska, Amy Orme, Hannah Orme, Laura O'Rourke, Charlotte Orr, Sarah Orr, Christopher Orton, Anna Osadcow, Rawlings Osagie, Rostam Osanlou, Lynn Osborne, Nigel Osborne, Rebecca Osborne, Wendy Osborne, William Osborne, Charles Osbourne, Jennifer Osei-Bobie, Joseph Osman, Wa'el Osman, Bashir Osman, G Osoata, Marlies Ostermann, Eoin O'Sullivan, Susan O'Sullivan, Noor Otey, Otheroro K. Otite, Marie O'Toole, Jingxiu Ouyang, Rachel Owen, Stephanie Owen, Emma Owens, Yetunde Owoseni, Michael Owston, Ruth Oxlade, Feray Ozdes, Jamie Pack, Alice Packham, Sophie Packham, Piotr Paczko, Grace Padden, Anand Padmakumar, Catrin Page, Iain Page, Joseph Page, Shannon Page, Valerie Page, Jodi Paget, Katherine Pagett, Lee Paisley, Susannah Pajak, Susie Pajak, Glykeria Pakou, Angela Pakozdi, Soubhik Pal, Sushi Pal, April Palacios, Vishnu B Palagiri Sai, Vadivu Palaniappan, Priya Palanivelu, Adrian Palfreeman, Heather Palfrey, Vinod Palissery, Deepshikha Palit, Sherly Pallipparambil Antony, Jason Palman, Alistair Palmer, Helen Palmer, Janet Palmer, Lynne Palmer, Ross Palmer, Andrea Pambouka, Ian Pamphlett, Daniel Pan, Anmol Pandey, Nithya Pandian, Krishnaa Pandya, Tej Pandya, Alice Panes, Jessica Pang, Yee Wei Pang, Laura Pannell, Kanwar Pannu, Suman Pant, Sathianathan Panthakalam, Charles T Pantin, Norman Pao, Helen Papaconstantinou, Padmasayee Papineni, Kitty Paques, Abdul W Paracha, Kerry Paradowski, Vinay Parambil, Supathum Paranamana, Siddhant R Parashar, Ian Parberry, Amy Parekh, Dhruv Parekh, Louise Parfitt, Helen Parfrey, Omi Parikh, Gemma Parish, John Park, Vivak Parkash, Angela Parker, Ben Parker, Emma Parker, Helen Parker, Jacob Parker, Julie Parker, Laura Parker, Lucy Parker, Natasha Parker, Nicola Parker, Sara Parker, Sean Parker, Kirstin Parkin, Anna Parkinson, Molly Parkinson, Valerie Parkinson, Chetan Parmar, Viraj Parmar, Victoria Parris, Chloe Parrish, Bethan Parry, Helen C Parry, Siobhan Parslow-Williams, Maria Parsonage, Georgie Parsons, Joseph Parsons, Penny Parsons, Richard Partridge, Zeeshan Parvez, Kevin Parvin, Lauren Passby, Samuel Passey, Heather Passmore, Juan Pastrana, Jonathan Patachako, Mital Patal, Sarah Patch, Aamie Patel, Alkesh Patel, Amisha Patel, Bijal Patel, Dakshesh Patel, Darshna Patel, Hemani Patel, Jaymik Patel, Kamal Patel, Kayur Patel, Kiran Patel, Krish Patel, Manish Patel, Margi Patel, Martyn Patel, Mehul Patel, Naleem Patel, Nehalbhai Patel, Prital Patel, Saagar Patel, Sanjay Patel, Soonie Patel, Trishna Patel, Zaakirah Patel, Vishal Patel, Kirsteen Paterson, Sangeeta Pathak, Nazima Pathan, Alexandra Patience, Donna Patience, Russell Patmore, Sonia Patole, Lia Paton, Abigail Patrick, Georgie Patrick, Jean Patrick, Simon Patten, Ben Pattenden, Charlotte Patterson, Jean Patterson, Linda Patterson, Molly Patterson, Robert Patterson, Martin Pattrick, Kamala Paudel, Manu Paudel, Suman Paudel, Moriarty Paul, Suman Paul, Leigh Pauls, Stephane Paulus, Amelia Pavely, Matthew J Pavitt, Susan Pavord, Brendan Payne, Elizabeth Payne, Mark Payne, Ruth Payne, Linda Peacock, Louise Peacock, Sarah Peacock, Henry Peake, Jasmine Pearce, Rachel Pearse, Rupert Pearse, Andrew Pearson, Daniel Pearson, Harriet Pearson, Karen Pearson, Samuel A Pearson, Sandra Pearson, Alice Peasley, Hilary Peddie, Steven Peebles, Russell Peek, Adam Peer, Shahid Peerbhoy, Claire Pegg, Eleanor Peggie, Hannah Peggie, Suzannah Peglar, Benjamin H Peirce, Mary Peirse, Claire Pelham, Abigail Pemberton, Melchizedek Penacerrada, Anthony Pender, Carmel Pendlebury, Jessica Pendlebury, Rachel Penfold, Catherine Penman, Julie Penman, Rachel Penman, Justin Penner, Kristi Penney, Anna Pennington, James Penny, Justin Pepperell, Rachel Percival, Adriana Pereira, Rita Pereira, Carlota Pereira Dias Alves, Indika Perera, Marek Perera, Elena Perez, Jane Perez, Tanaraj Perinpanathan, Lakshmi Periyasamy, Emma Perkins, Ida Pernicova, Elizabeth Perritt, Alice Perry, Alison Perry, Emily Perry, Meghan Perry, Thomas M Perumpral, Guilherme Pessoa-Amorim, Ruth Petch, Lionel Peter, Cecilia Peters, Lucy Peters, Mark Peters, Steve Peters, Tim Peters, Remy Petersen, Alexandra Peterson, Leon Peto, Iulia Petras, Boyanka Petrova, Mirela Petrova, Ellen Petrovics, Tom Pettigrew, Marius Pezard-Snell, Paul Pfeffer, Gunjan Phalod, Mysore Phanish, Paul Phelan, Christopher Philbey, Jennifer Philbin, Aled Phillips, Alex Phillips, Bethan Phillips, Dylan Phillips, Nicola Phillips, Patrick Phillips, Rachael Phillips, Thomas Phillips, Marie Phipps, Michael Phipps, Virach Phongsathorn, Mandeep Phull, Anita Phuyal, Aye Kyaw Phyo, Myat T T PI, Sara Pick, James Pickard, Charlotte Pickering, Frances Pickering, Gillian Pickering, Thomas Pickett, Joanna Pickles, Shirley Pickstock, Benjamin Pickwell-Smith, Natalia Pieniazek, Charlie Piercy, Angelo Pieris, Samia Pilgrim, Paul A Pillai, Laura Pilling, Zoe Pilsworth, Heather Pinches, Stacey Pinches, Kirsty Pine, Muni T Pinjala, Stefania Pintus, Graeme Piper, Tasneem Pirani, Marie Pitchford, Marcus Pittman, Sally Pitts, Nicolene Plaatjies, Naomi Platt, Robert Pleass, Megan Plowright, Laura Plummer, Charles Plumptre, Jonathan Pobjoy, Tatiana Pogreban, Stephen Poku, Petra Polgarova, Rachel Pollard, Louisa Pollock, Oluwamayowa Poluyi, Gary John Polwarth, Fiona Pomery, Ida M F Ponce, Ponmurugan Ponnusamy, Suresh Ponnusamy, Aravind Ponnuswamy, Inês Ponte Bettencourt dos Reis, Suman Pooboni, Alice Poole, Lynda Poole, Michele Poole, Sharon Poon, Tajinder Poonian, Jack Porteous, Mark Porteous, David Porter, Jo Porter, Linda Porter, Ross Porter, Anna Posada, Kelly Postlethwaite, Manoj Potdar, Carla Pothecary, Narayana Pothina, Priyadarshan Potla, Dorota Potoczna, Jason Pott, Alison Potter, Andrew Potter, Jean Potter, Sarah Potter, Tracey Potter, Elspeth Potton, Joanne B Potts, Julie Potts, Kathryn Potts, Beli Poudyal, Una Poultney, Katherine Poulton, Vanessa Poustie, James Powell, Jordan Powell, Natassia Powell, Sandra Powell, Deborah Power, Nick Power, Sharon Power, Joseph Poxon, Emma Poyner, Robin Poyner, Aishwarya Prabhu, Sandhi Prabowo, Vidushi Pradhan, Gyanwali Pradip, Helena Prady, Aalekh Prasad, Krishna Prasad, Usha Prasad, Fredy Prasanth Raj, Sangeetha Prasath, Anezka Prately, Nicky G Pratiwi, Steven Pratt, David Preiss, Claire Prendergast, Lynn Prentice, Peter Prentice, Verity Prescott, Laura Presland, Catharine Prest, Stephen Preston, Martha Pretorius, Natalie Prevatt, Sandra Prew, Ashley Price, Carly Price, Claire Price, David Price, Elizabeth Price, Katie Price, Laura J Price, Nathan Price, Vivien Price, Rachael Price-Eland, Anne Priest, Jimena Prieto, Lorraine Primrose, Clare Prince, John Prince, Judith Prince, Laura Prince, Shirley Pringle, Melissa Prior-Ong, Veronika Pristopan, Kelly Pritchard, Lucy Pritchard, Simon Pritchard, Verma Priyash, Andrew Procter, Clare Proctor, Mike Protopapas, Rebecca Proudfoot, Benjamin Prudon, David Pryor, Solomon Pudi, Angela Puffett, Joanne Pugh, Lawrence Pugh, Mark T Pugh, Nichola Pugh, Richard Pugh, Veronika Puisa, Safa Puliyakkadi, Jennifer Pullen, Kirandip Punia, Saleel Punnilath Abdulsamad, Laura Purandare, Daniel Purchase, Corrina Purdue, Rachel Purdy, Bally Purewal, Rebecca Purnell, Molly Pursell, Gregory Purssord, Rory Purves, Sarah Purvis, Khairunnisa Puspatriani, Daniel Putensen, Bhamini Puvaneswaran, Alex Puxty, Kathryn Puxty, Zoe Puyrigaud, Eleanor Pyart, Emily Pye, Michael Pynn, Tariq Qadeer, Mohammad Qayum, Corrine Quah, Sheena Quaid, Nathaniel Quail, Charlotte Quamina, Georgia Quartermaine, Tara Quasim, Alice Quayle, Eleanor Quek, Siobhan Quenby, Xinyi Qui, Vanessa Quick, Julie Quigley, Juan-Carlos Quijano-Campos, Jhanielle Quindoyos, Andrew Quinn, James Quinn, Tom Quinn, Quratulain Quratulain, Danya Qureshi, Ehsaan Qureshi, Hasanain Qureshi, Iram Qureshi, Khadija Qureshi, Nawaz Qureshi, Qurratulain Qurratulain, Saad Qutab, Muhammad S Rabbani, Simon Rabinowicz, Madalina Raceala, Alan Rachid, Raissa Rachman, Laura Rad, Jane Radford, Liz Radford, Jayachandran Radhakrishnan, Hannah Rafferty, Muhammad Y Rafiq, Shabana Rafiq, Cecillia Rafique, Jethin Rafique, Muhammad Rafique, Ravi Ragatha, Aiswarya Raghunathan, Abigail Raguro, Shankho D Raha, Sana Rahama, Mutia Rahardjani, Karen Rahilly, Faisal Rahim, Abdul H Rahimi, Haseena R Rahimi, Muhammad Rahman, Salim Ur Rahman, Sohail Rahmany, Prajan Rai, Sabi Rai, Supriya Rai, Lenka Raisova, Ajay Raithatha, Arjun Raj, Anithya Rajagopal, Pradeep Rajagopalan, Nithy Rajaiah, Kanendran Rajalingam, Arvind Rajasekaran, Aylur Rajasri, Sagar Rajbhandari, Thurkka Rajeswaran, Jyothi Rajeswary, Jeyanthy Rajkanna, Indhuja Rajkumar, Gayathri Rajmohan, Ruth Rallan, Katherine Ralston, Maximilian Ralston, Matsa Ram, Balaji Ramabhadran, Fathima Ramali, Mohamed Ramali, Athimalaipet Ramanan, Shashikira Ramanna, Maheshi Ramasamy, Irfah Rambe, Aradhana Ramchandani, Dhanishta Ramdin, Jozel Ramirez, Mylah Ramirez, Geshwin Ramnarain, Amrita Ramnarine, Lidia Ramos, Tommy Rampling, Shanthi Ramraj, Janine Ramsay, Alex Ramshaw, Aleem Rana, Ghulam F Rana, Naeem Rana, Rehman Rana, Abby Rand, James Rand, Harpal Randheva, Poonam Ranga, Manmeet Rangar, Harini Rangarajan, Sameer Ranjan, Hannah Rank, Poormina Ranka, Rajesh Rankhelawon, Alastair Rankin, Anita Rao, Sandhya Rao, Sanjay Rao, Deepak Rao, Althaf Abdul Rasheed, Khalid Rashid, Madeleine Rason, Valentina Raspa, Somil Rastogi, Fazila Rasul, Simbisai Ratcliff, Sam Ratcliffe, Sophy Ratcliffe, Priti Rath, Sanjeev Rath, Mohmad I Rather, Krishnaraj Rathod, Selina Rathore, Aravinden Ratnakumar, Jonathan Ratoff, Deepa Rattehalli, Davide Ravaccia, Malvi Raval, Phil Ravencroft, Jason Raw, Rachael Raw, Manisha Rawal, Sulaiman A Rawashdeh, Hywel Rawlins, Gautam Ray, Adam Raymond-White, Dana Raynard, Helen Rayner, Nicola Rayner, Amy Raynsford, Salman Razvi, Zarine Razvi, Kerry Read, Sarah Read, Michael Reay, Anne Reddington, Ajay Reddy, Anvesh Reddy, Harsha Reddy, Heidi Redfearn, Aine Redfern-Walsh, Ianto Redknap, Nell Redman, Alex Redome, Joan Redome, Anna Reed, John Reed, Andrew Rees, Caitlin Rees, James Rees, Martyn Rees, Sarah Rees, Stephanie Rees, Tabitha Rees, Elinor Rees-Jones, Fiona Regan, Karen Regan, Martin Regan, Susan Regan, Kanchan Rege, Ahmed Rehan, A Rehman, Hafiz Rehman, Shoib Rehman, Zainab Rehman, Ada Reid, Andrew Reid, Jennifer Reid, Jeremy Reid, Sharon Reid, Mkyla Reilly, Sharon Reilly, Christina Reith, Arisa Reka, Alda Remegoso, Dinakaran Rengan, Louise Renouf, Stephen Renshaw, Remya Renu Vattekkat, Henrik Reschreiter, Mark Revels, Adam Revill, Glynis Rewitzky, Severine Rey, Charles Reynard, Dominic Reynish, Heather Reynolds, Peter Reynolds, Piero Reynolds, Jonathan Rhodes, Naghma Riaz, Patricia Ribeiro, Emily Rice, Matthew Rice, Natalie Rice, Mel Rich, Alex Richards, Alison Richards, Liz Richards, Suzanne Richards, Celia Richardson, Emma Richardson, Fiona Richardson, Jamie Richardson, Julie Richardson, Madeleine Richardson, Neil Richardson, Nicky Richardson, Joanne Riches, Katie Riches, Leah Richmond, Ruth Richmond, William Ricketts, Hannah Rickman, Anna Riddell, Stephanie Ridgway, Mohamed Ridha, Carrie Ridley, Paul Ridley, Gudrun Rieck, Linsey Rigby, Myckala Rigby, Daniel Rigler, Samita Rijal, Nur Rika, Hannah Riley, Matthew Riley, Phil Riley, Atika Rimainar, Zwesty V P Rimba, Dominic Rimmer, Robert Rintoul, Andrew Riordan, David Ripley, Naomi Rippon, Chloe Rishton, Michael Riste, David Ritchie, Jane Ritchie, Andy Ritchings, Pilar Rivera Ortega, Vanessa Rivers, Batool Rizvi, Syed A S Rizvi, Syed H M Rizvi, James Robb, Edel Robbins, Gareth Roberts, Ian Roberts, Jane Roberts, Jean Roberts, Karen Roberts, Mark Roberts, Nicky Roberts, Philip Roberts, Rebecca Roberts, Victoria Roberts, Calum Robertson, James Robertson, Jamie Robertson, Kirsten Robertson, Nichola Robertson, Stuart Robertson, Mhairi Robertson, Nicole Robin, Caroline Robinson, Emma Robinson, Gisela Robinson, Hannah Robinson, Jemima Robinson, Kate Robinson, Liz Robinson, Matthew Robinson, Nicky Robinson, Richard Robinson, Ryan Robinson, Sandra Robinson, Alexandra Robinson, Steve Robson, Lisa Roche, Samantha Roche, Natalie Rodden, Alistair Roddick, Elin Roddy, Jack Roddy, Marion Roderick, Alison Rodger, Faye Rodger, Megan Rodger, Michael Rodger, Megan Rodger, Alicia Rodgers, Deirdre Rodgers, Natasha Rodgers, Penny Rodgers, Rocio Rodriguez-Belmonte, Nicholas Roe, Charles Roehr, Charles Roehr, Gill Rogers, Jason Rogers, Joanne Rogers, John Rogers, Leigh Rogers, Lindsay Rogers, Michaela Rogers, Paula Rogers, Susan Rogers, Thomas Rogers, Paula Rogers, Jana Rojkova, Sakib Rokadiya, Lee Rollins, Jennifer Rollo, Catherine Rolls, Alexander Rond-Alliston, Claire Rook, Kevin Rooney, Lynsey Rooney, Lace P Rosaroso, Emily J Rosby, Abigail Rose, Alastair Rose, Annie Rose, Steve Rose, Zoe Rose, Josephine Rosier, Anna Roskilly, Gavin A Ross, Isobel Ross, Jack Ross, Jennifer Rossdale, Andrew Ross-Parker, Anthony Rostron, Alex Rothman, Joanne Rothwell, Lindsay Roughley, Catherine A Rourke, Kathryn Rowan, Neil Rowan, Stephen Rowan, Anna Rowe, Nicola Rowe, Louise Rowe-Leete, Benjamin Rowlands, Elen Rowlands, Megan Rowley, Subarna Roy, Matthew Roycroft, Anna Roynon-Reed, Ann R Royson, Sam Rozewicz, Anna Rudenko, Senthan Rudrakumar, Banu Rudran, Shannon Ruff, Prita Rughani, Rory Rule, Sharon Rundell, Eve Rushforth, Jeremy Rushmer, Darren Rusk, Peter Russell, Richard Russell, Cristina Russo, Marieke Rutgers, Krzysztof Rutkowski, Aidan Ryan, Brendan Ryan, Kathryn Ryan, Lucy Ryan, Matthew Ryan, Pat Ryan, Phil Ryan, Declan Ryan-Wakeling, Elena Rybka, Maggie Ryder, Sophie Ryder, M Saad, Gwendolyn Saalmink, Javeson Sabale, Suganya Sabaretnam, Noman Sadiq, Emma Sadler, Ashiq Saffy, Beth Sage, Harkiran Sagoo, Sobia Sagrir, Rajnish Saha, Sian Saha, Nikhil Sahdev, Sarvjit Sahedra, Jagdeep Sahota, Nooria Said, Shalini Saini, Bryony Saint, Anam Sajid, Sreekanth Sakthi, Hikari Sakuri, Murthy Saladi, Abdul Salam, Armorel Salberg, Erika Salciute, Gina Saleeb, Mumtaz Saleh, Hizni Salih, Laylan Salih, Duha Salim, Sarah Salisbury, SiteEneye Saliu, Rustam Salman, Jenny Salmon, Rebecca Salmon, Dario Salutous, Mfon Sam, Sally Sam, Tinashe Samakomva, Razan Saman, Sakeena Samar, Selva Saminathan, Renaldo Samlal, Emily Sammons, David Sammut, Mark Sammut, Saud Sammut, Thomas Sammut, Sunitha Sampath, Claire Sampson, Julia Sampson, Aashna Samson, Anda Samson, Johnson Samuel, Merna Samuel, Reena Samuel, Thomas D L Samuel, Younan Samuel, Kevin Samuels, Theo Samuels, Joanna Samways, Manjula Samyraju, Ilves Sana, Veronica Sanchez, Amada Sanchez Gonzalez, Alina Sanda-Gomez, Peter Sandercock, Jack Sanders, Amy Sanderson, Tom Sanderson, Kuljinder Sandhu, Loveleen Sandhu, Sam Sandow, Victoria Sandrey, Sarah Sands, Langizya Sanga, Harwinder Sangha, Jasmine Sanghera, Mirriam Sangombe, Mathew Sanju, Lakshmi Sankaran, Filipa Santos, Catarina Santos Ferreira De Almeida, Rojy Santosh, Jayanta Sanyal, Aureo F Sanz-Cepero, Yunima Sapkota, Dan Saragih, Dinesh Saralaya, Anggita Saraswati, Arun Saraswatula, Pira Saravanamuthu, Joshua Sarella, Avishay Sarfatti, Rebecca Sargent, Beatrix Sari, Diah Sari, Khatija Sarkar, Rahuldeb Sarkar, Sruthi Sarma, Paulo Sarmiento, Zainab Sarwar, Thea Sass, Krishna Satchithananthasivam, Sonia Sathe, Sobitha Sathianandan, Abilash Sathyanarayanan, Lavanya S J P Sathyanarayanan, Thozhukat Sathyapalan, Prakash Satodia, Vera Saulite, Andrew Saunders, Rachel Saunders, Samantha Saunders, Anne Saunderson, Heather Savill, Karishma Savlani, Gauri Saxena, Matthew Saxton, Amrinder Sayan, Ian Sayers, Diane Scaletta, Deborah Scanlon, Jeremy Scanlon, Lyndsay Scarratt, Sean Scattergood, Alvin Schadenberg, Jenna Schafers, Wendy Schneblen, Ella Schofield, Rebecca Schofield, Samuel Schofield, David Scholes, Karen Scholes, Alex Schoolmeesters, Natasha Schumacher, Nicola Schunke, Martin Schuster Bruce, Karin Schwarz, Antonia Scobie, Thomas Scoones, Tim Scorrer, Alex Scott, Alistair Scott, Anne Scott, Catherine Scott, Christine Scott, Emily Scott, Kathyn Scott, Leanne Scott, Martha Scott, Stephen Scott, Timothy Scott, Zoe Scott, Sarah Scourfield, Wendy Scrase, Nicholas A Scriven, Angela Scullion, Therese Scullion, Emily Seager, Cathy Seagrave, Rebecca Seaman, Eleanor Sear, Isabella Seaton, Annette Seatter, Anna Seckington, Joanna Sedano, Gabrielle Seddon, Gemma Sedgwick, Yee Yong See, Muhammad A Seelarbokus, Christopher Sefton, Matias Segovia, Fatima Seidu, Gillian Sekadde, Faye Selby, Georgina Selby, Claire Sellar, Rachael Sellars, Katharine Sellers, Joseph Selley, Victoria Sellick, Gobika Selvadurai, Brintha Selvarajah, Haresh Selvaskandan, Subothini S Selvendran, Jeyakumar Selwyn, Alison Semmens, Gary Semple, Michael Sen, Nandini Sen, Seema Sen, Aditya Sengupta, Niladri Sengupta, Susana Senra, HoJan Senya, Teona Serafimova, Elisa Sernicola, Dheeraj Sethi, Shivam Sethi, Niranjan Setty, Abigail Seward, Teswaree Sewdin, Terri-Ann Sewell, Jack Seymour, Katherine Seymour, Hussam Shabbir, Fiona Shackley, Tariq Shafi, Faiza Shafique, Aashni Shah, Ahmar Shah, Anand Shah, Bhavni Shah, Hussun-Ara Shah, Momin Shah, Neil Shah, Pallav Shah, Priyank Shah, Qasim Shah, Rupal Shah, Sarfaraz H Shah, Snehal Shah, Suraj Shah, Syed Shah, Wajid Shah, Saarma Shahad, Sousan Shahi, Sipan Shahnazari, Najam Shahzad, Muhammad Shahzeb, Aisha Shaibu, Zara Shaida, Amina Y Shaikh, Maliha Shaikh, Rajit Shail, Mariya Shaji, Muhammad Shakeel, Korah Shalan, Jack Shallcross, Nadia Shamim, Ummulbaneen Shamji, Anindya Shams, Kazi Shams, Rasha Shamsah, Thomas Shanahan, Hamed Sharaf, Asir Sharif, Ajay Sharma, Ash Sharma, Bhawna Sharma, Mona Sharma, Ojasvi Sharma, Poonam Sharma, Rajeev Sharma, Sagar Sharma, Sanjeev Sharma, Sarkhara Sharma, Seema D Sharma, Shriv Sharma, Sonal Sharma, Alexander Sharp, Charles Sharp, Gemma Sharp, Kerry Sharp, Louise M Sharp, Paula Sharratt, Phoebe Sharratt, Katherine Sharrocks, Serene Shashaa, Alexandra Shaw, Christopher Shaw, Daisy Shaw, David Shaw, Deborah Shaw, Joanne Shaw, Jonathon Shaw, Lisa Shaw, Michele Shaw, Tomos G Shaw, Anna Shawcross, James Shawcross, Jill Shawe, Lou Shayler, Sophy Shedwell, Jonathan Sheffield, Zak Shehata, Arshiya Sheik, Asif Sheikh, Noorann Sheikh, Benjamin Shelley, Sarah Shelton, Anil Shenoy, Julie Shenton, Sarah Shephardson, Amy Shepherd, Kate Shepherd, Lorna Shepherd, Scott Shepherd, Gareth Sheppard, Rhian Sheppeard, Helen Sheridan, Ray Sheridan, Samuel Sherridan, Leanne Sherris, Susanna Sherwin, Shaad Shibly, Fathimath F Shiham, Cassandra Shilladay, Ben Shillitoe, Delane Shingadia, Chiaki Shioi, Anand Shirgaonkar, Kim Shirley, Helen Shirt, Adebusola Shonubi, Jane Shoote, Ruth Shorrocks, Rob Shortman, Rohan Shotton, Sarah Shotton, Charmaine Shovelton, Ervin Shpuza, Anil Shrestha, Apurba Shrestha, Nora Shrestha, Suchita Shrestha, Karen Shuker, Jonathan Shurlock, Jack Shurmer, Erfanur R Shuvo, Gilbert Siame, Loria Siamia, Seshnag Siddavaram, Nasir Siddique, Shahla Siddique, Sohail Siddique, Elaine Siddle, Emma Sidebotham, Jo Sidebottom, Roy Sievers, Katie Siggens, Nyma Sikondari, Sofia V Silva, Claudia Silva Moniz, Malcolm Sim, Theresa Simangan, Vimbai Simbi, Robert Sime, George Simmons, Oliver Simmons, Richard Simms, Laura Simon, Merritt Simon, Natalie Simon, Samantha Simpkins, Angela Simpson, Anna Simpson, Danny Simpson, Georgina Simpson, Joanne Simpson, Kerry Simpson, Maria Simpson, Phillip Simpson, Thomas Simpson, Kathryn Simpson, Simon Sinclair, Cindy Sing, Ankita Singh, Claire Singh, Davinder Singh, Jayaprakash Singh, Jyoti Singh, Lokeshwar Singh, Manjeet Singh, Nadira Singh, Pankaj Singh, Prabhsimran Singh, Salil Singh, Saurabh Singh, Parag Singhal, Bryan Singizi, Vicky Singler, Manas Sinha, Punam Sinha, Sharad Sinha, Utkarsh Sinha, Guy Sisson, Sarah Sithiravel, Karthikadevi Sivakumar, Shanmugasundaram Sivakumar, Darsh Sivakumran, Sivanthi Sivanadarajah, Pasupathy-Rajah Sivasothy, Asok Skaria, Nicole Skehan, Robert Skelly, Orlagh Skelton, Imogen Skene, Denise Skinner, Tabitha Skinner, Victoria Skinner, Agnieszka Skorko, Iwona Skorupinska, Mariola Skorupinska, Amy Slack, Katie Slack, Heather Slade, Mark Slade, Lynda Slater, Nicola Slawson, Rachel Slingsby, Andrew Sloan, Brendan Sloan, Derek Sloan, Geraldine Sloane, Malgorzata Slowinska, Benjamin Small, Ellen Small, Samuel Small, Adam Smallridge, Dawn Smalls, Karen D Smallshaw, Andy Smallwood, Brittany Smart, Lynne Smart, Justyna Smeaton, Carien Smit, Aileen Smith, Alex Smith, Amanda Smith, Amy Smith, Andrew Smith, Catherine Smith, Chris Smith, Christopher Smith, Coral Smith, Debra Smith, Dominic Smith, Eleanor Smith, Harriet Smith, Hazel Smith, Helen Smith, Jacky Smith, Jessica Smith, Kate Smith, Kerry Smith, Lara Smith, Linda Smith, Lisa Smith, Loren Smith, Maria Smith, Mark Smith, Martha A Smith, Mel Smith, Nicholas Smith, Oliver Smith, Philippa Smith, Rachel Smith, Rebecca Smith, Rheanna Smith, Richard Smith, Sally Smith, Samantha Smith, Stacey Smith, Stephanie Smith, Susan Smith, Tracy Smith, Victoria Smith, Imogen Smith, John Smith, Sue Smolen, Sara Smuts, Jennifer Smyth, Naoise Smyth, Annette Snell, David Snell, Luke Snell, Alfred So, Beng So, Michelle Soan, Toluleyi Sobande, Sonia Sobowiec Kouman, Alberto Sobrino Diaz, Basit Sohail, Bina Sohail, Herminder Sohal, Roy Soiza, Olajumoke Solademi, Babak Soleimani, Amanda Solesbury, Mohamed Soliman, Bernadette Solis, Reanne Solly, Louise Solomon, Subash Somalanka, Chandrashekaraiah Somashekar, Sven Sommerfield, Govind Soni, Raj Sonia, Temi Sonoiki, Shiu-Ching Soo, Pavandeep Soor, Germander Soothill, Jennifer Soren, Apina Sothinathan, Pragalathan Sothirajah, Jessica Sousa, Najwa Soussi, Donna Southam, David Southern, Iain Southern, Louise Southern, Sara M Southin, Jessica Southwell, Thomas Southworth, Sandra Sowden, Jason Sowter, Claudia Spalding, Enti Spata, Catherine Speare, Katie Spears, Mark Spears, Lynne Speirs, Susan Speirs, Michelle Spence, Niamh Spence, Branwell Spencer, Gisele Spencer, Rebecca Spencer, Sue Spencer, Tom Spencer, Helen Spickett, Jennifer Spillane, William Spiller, Kerry Spinks, Michelle Spinks, Nick Spittle, Sarah Spray, Janet Spriggs, Oliver Spring, Gemma Squires, Jack Squires, Rebecca Squires, Ram Sreenivasan, Shiva Sreenivasan, K Sri Paranthamen, Rajesh Srikantaiah, Koottalai Srinivasan, Ramesh Srinivasan, Asha Srirajamadhuveeti, Vino Srirathan, Rhodri Stacey, Sybil Stacpoole, Louise Stadon, William J Stagg, Jocasta Staines, Nikki Staines, Katie Stammers, Roxana Stanciu, Grazyna Stanczuk, Thomas Standley, Brittany Staniforth, Andrew Stanton, Lisa Stanton, Robyn Staples, Simon Stapley, Natalie Staplin, Adam Stark, Elizabeth Starkey, David S Starnes, Michelle Starr, Rachel Stead, Clare Stebbing, Conor Steele, Henry Steer, John Steer, Vergnano Stefania, Paula Stefanowska, Femke Steffensen, Caroline Stemp, Emma Stenson, Alison Stephens, David Stephensen, Elaine Stephenson, Monique Sterrenburg, Jennifer Stevens, Melanie Stevens, Will Stevens, Amy Stevenson, Andrew Stevenson, Lesley Stevenson, Sarah Stevenson, Matthew Steward, Christopher Stewart, Claire Stewart, Colin Stewart, Dobree A Stewart, Katelyn Stewart, Keely Stewart, Kristina Stewart, McKenna Stewart, Melissa Stewart, Rachel Stewart, Richard Stewart, Jo Stickley, Gemma Stiller, Sarah Stirrup, Sarah Stock, Alexander Stockdale, Danielle Stocker, Lynne Stockham, Paul Stockton, Emma Stoddard, Chelsea Stokes, Chris Stokes, Ben Stone, Roisin Stone, Sarah Stone, Emma-Jane Stoner, Imogen Storey, Kim Storton, Frederick Stourton, Angela Strachan, Catherine Strait, Emma Stratton, Jane Stratton, Sam Straw, Dieter Streit, Emma Stride, Sally Stringer, Sophia Strong-Sheldrake, Siske Struik, Carmel Stuart, Anna Stubbs, Harrison Stubbs, Ann Sturdy, Sharon Sturney, Matt Stuttard, Cristina Suarez, Karuna Subba, Christian P Subbe, Keerth Subramaniam, Manjula Subramanian, Vaidyanathan Subramanian, Venkatram Subramanian, Chinari Subudhi, Rebecca Suckling, Srivatsan Sudershan, Peter Sugden, Putri A Suherman, Rudresh Sukla, Anila Sukumaran, Ezoubi Suleiman, Ali Suliman, Fatimah Suliman, Sugrah Sultan, Samyukta Sundar, Radha Sundaram, Reka Sundhar, Edmond Sung, Nadia Sunni, Jay Suntharalingam, Amitava Sur, Dharmic Suresh, Nayanika Suresh, Shilpa Suresh, Michael Surtees, C Susan, Danielle Suter, Rohan Suthar, Helen Sutherland, Rachel Sutherland, Rebecca Sutherland, Sara-Beth Sutherland, Dovile Sutinyte, Deborah Sutton, Sam Sutton, Siobhan Sutton, Mihaela Sutu, Marie-Louise Svensson, Sima Svirpliene, Andrew Swain, Rose Swain, Thomas Swaine, Christopher Swales, Carla Swanson-Low, Tirion Swart, Stephen Sweetman, Ealish Swift, Paul Swift, Pauline Swift, Rachael Swift, Rachel Swingler, Sophie Swinhoe, Katarzyna Swist-Szulik, Luke Swithenbank, Omair Syed, Catriona Sykes, Daisy Sykes, Eliot Sykes, Luke Sylvester, Declan Symington, Dominic Symon, Andrew Syndercombe, Zoe Syrimi, Jen Syson, Gemma Szabo, Denise Szabó, Tamas Szakmany, Nina Szarazova, Megan Szekely, Adele Szekeres, Matthew Szeto, Kinga Szymiczek, Maria Tadros, Amr Tageldin, Lucy Tague, Hasan Tahir, Muhammad Tahir, Michael Tai, Jennifer Tait, Abigail Takyi, Peter Talbot, Alison Talbot -Smith, James Talbot-Ponsonby, Richard Tallent, Bradley Tallon, Adrian Tan, Bee T T Tan, Hock Tan, Huey Tan, Jenny Tan, Jun Seng Tan, Keith Tan, Wei Teen Tan, Anand Tana, Alex Tanner, Christina Tanney, Tabitha Tanqueray, Emma Tanton, Anita Tantri, Tm Tanzil-Al-Imran, Hayley Tarft, Priyal Taribagil, Obaid Tarin, Syed Tariq, David Tarpey, Esther Tarr, Lisa Tarrant, Antonia Tasiou, Alex Tate, Margaret L Tate, Matthew Tate, Paul Tate, Kate Tatham, Silvia S Tavares, Vera Tavoukjian, Shao An Isaac Tay, Alexander Taylor, Beverley Taylor, Charlie Taylor, Charlotte A Taylor, David Taylor, Elisabeth Taylor, Holly Taylor, Janet Taylor, Jennifer Taylor, Joanne Taylor, Julie Taylor, Karen Taylor, Leanne Taylor, Margaret Taylor, Matthew Taylor, Melanie Taylor, Natalie Taylor, Rachael Taylor, Rachel Taylor, Samantha Taylor, Suzanne Taylor, Tracey Taylor, Vicky Taylor, Victoria Taylor, Michelle Taylor-Siddons, Thomas Taynton, Amelia Te, Francesca Teasdale, Jessica Teasdale, Katherine Teasdale, Julie Tebbutt, Caroline Tee, Izla Teeluck, Borja Tejero Moya, Rajni Tejwani, Adam Telfer, Vibha Teli, Jennifer Tempany, Julie Temple, Natalie Temple, Helen Tench, Yi He Teoh, Radoslaw Tereszkowski-Kaminski, Lynne Terrett, Louise Terry, Tesha I M Tesha, Dariusz Tetla, Shirish Tewari, Daniel Tewkesbury, Joana Texeira, ChiaLing Tey, Miriam Thake, Clare Thakker, Manish Thakker, Jayna Thakrar, Bhavneet Thamu, Hilary Thatcher, Andrew Thayanandan, Krishna Thazhatheyil, Eaint Thein, Lambrini Theocharidou, Phyu Thet, Kapeendran Thevarajah, Mayooran Thevendra, Nang Thiri Phoo, Yvette Thirlwall, Muthu Thirumaran, Alice Thomas, Andrew Thomas, Caradog Thomas, Emma Thomas, Enson Thomas, Esther Thomas, Helen Thomas, James Thomas, Jordan L Thomas, Karen Thomas, Koshy Thomas, Lucy Thomas, Rachel Thomas, Rebecca Thomas, Rhys Thomas, Ruth Thomas, Sarah Thomas, Sherine Thomas, Tessy Thomas, Vicky Thomas, Kirsty Thomasson, Rhian Thomas-Turner, Catherine Thompson, Christopher Thompson, Clara Thompson, Emma Thompson, Fiona Thompson, Hilary Thompson, Jessica Thompson, Katharine Thompson, Laura Thompson, Liz Thompson, Luke Thompson, Michael Thompson, Orla Thompson, Rebecca Thompson, Roger Thompson, Yolanda Thompson, Nicola Thomson, Patrick Thorburn, Natasha Thorn, Charlotte Thorne, Nicola Thorne, Alice Thornton, Danielle Thornton, Jim Thornton, Richard Thornton, Sara Thornton, Susan Thornton, Thomas Thornton, Tracey Thornton, Christopher Thorpe, Nigel Thorpe, Sarah Thorpe, Paradeep Thozthumparambil, Laura Thrasyvoulou, Hannah Thraves, Genevieve Thueux, Pinky Thu-Ta, Vicky Thwaiotes, Guy Thwaites, Simon Tiberi, Serena Tieger, Carey Tierney, Caroline Tierney, Mark Tighe, Sorrell Tilbey, Christopher Till, Amanda Tiller, Heather Tiller, John Timerick, Elizabeth Timlick, Alan Timmins, Alison Timmis, Hayley Timms, Anne-Marie Timoroksa, Samakomva Tinashe, Stephanie Tingley, Nicola Tinker, Heather Tinkler, Marianne Tinkler, Jacqui Tipper, Ajay Tirumalai Adisesh, Helen Tivenan, Katarzyna Tluchowska, Helen T-Michael, Anne Todd, Jackie Todd, Shirley Todd, Stacy Todd, Olga Toffoletti, Mohamed Tohfa, Sue Tohill, Melanie Tolson, Ana L Tomas, Natalia Tomasova, Sharon Tomlin, Simon Tomlins, Jo Tomlinson, Kerry Tomlinson, James Tonkin, Ivan Tonna, Catherine Toohey, Kirsty Topham, Mathew Topping, Audrey Torokwa, Collette Torrance, Omar Touma, Laura Tous Sampol, Ruhaif Tousis, Melissa Tout, Peter Tovey, Gareth Towersey, Jill Townley, Richard Tozer, Michelle Tran, Helen Tranter, Jenny Travers, Christopher Travill, Sarah Traynor, Leanne Trethowan, Estefania Treus Gude, Mary Trevelyan, Nicola A Trewick, Ascanio Tridente, Sanchia Triggs, Fiona Trim, Alex Trimmings, Tom Trinick, Kate Trivedi, Sven Troedson, Emily Tropman, Amy Trotter, Spyridon Trous, Helen Trower, Madeleine Trowsdale Stannard, Nigel Trudgill, Robert Truell, Nick Truman, Maria Truslove, Shaun Trussell, Tariq Trussell, Kyriaki Tsakiridou, Christine Tsang, Peter Tsang, Tan Tsawayo, Kyriaki K Tsilimpari, Georgios Tsinaslanidis, Simon Tso, Natasha Tucker, Sally Tucker, Stephanie Tucker, Daisy E Tudor, Aisha Tufail, Jess Tuff, Jemma Tuffney, Redmond Tully, Grace Tunesi, Killiam Turbitt, Rezon Turel, Tolga Turgut, Claudia Turley, Alison Turnbull, Aine Turner, Ash Turner, Charlotte Turner, Gail Turner, Georgina Turner, Kate Turner, Kelly Turner, Kim Turner, Lucy Turner, Lynne C Turner, Mark Turner, Patricia Turner, Sally Turner, Samantha Turner, Sarah Turner, Victoria Turner, Ian Turner-bone, Sharon Turney, Jon Turvey, Conor Tweed, David Tweed, Rebecca Twemlow, Emma Twohey, Bhavya Tyagi, Vedang Tyagi, Abigail Tyer, Amanda Tyler, Jayne Tyler, Jennifer Tyler, Alison Tyzack, Petros Tzavaras, Ioannis Tzinieris, A Wahid Uddin, Mohammad S Uddin, Ruhama Uddin, Ruzena Uddin, Jacinta Ugoji, Edith N Ukaegbu, Waqar Ul Hassan, Zia Ul-Haq, Salamat Ullah, Sana Ullah, Sanda Ullah, Ju Um, Athavan Umaipalan, Anil Umate, Judith Umeadi, Akudo Umeh, Wilfred Umeojiako, Ben Ummat, Emma Underhill, Charlotte Underwood, Jonathan Underwood, Adam Unsworth, Veerpal S Uppal, Gerry Upson, Masood Ur Rasool, Alison Uriel, Sebastian Urruela, Hiromi Uru, Jess Usher, Miranda Usher, Rebecca Usher, Alexandra Usher-Rea, Andrew Ustianowski, Hiromi Uzu, Linda C Vaccari, Uddhav Vaghela, Abhay Vaidya, Denny Vail, Bernardas Valecka, Jennifer Valentine, Balan Valeria, Pramodh Vallabhaneni, Nick Vallotton, Luke Vamplew, Ekaterini Vamvakiti, Joannis Vamvakopoulos, Lucia van Bruggen, Maud van de Venne, Alex van der Meer, Nora van der Stelt, Lynne van Koutrik, Alexander Van Loggerenberg, Joseph Vance-Daniel, Rama Vancheeswaran, Samuel I Vandeyoon, Padma Vankayalapati, Piyush Vanmali, Chloe Vansomeren, William Van't Hoff, Sejal Vara, Stehen J Vardy, Anu Varghese, Maria Varghese, William Varney, Giulia Varnier, Anna-Nektaria Varouxaki, Resti Varquez, Valeria Vasadi, Olivia Vass, Katherine Vassell, Vimal Vasu, Vasanthi Vasudevan, Manu Vatish, Sue Vaughan, Heloyes Vayalaman, Darini Vayapooree, Christopher Vaz, Niki Veale, Sachuda Veerasamy, Swati Velankar, Luxmi Velauthar, Neyme Veli, Nicola Vella, Aravind Velugupati, Anitha Velusamy, Ian Venables, Mavi Venditti, Ramya Venkataramakrishnan, Richard Venn, Robert Venn, Lyn Ventilacion, Joanne Vere, Mark Veres, Stefania Vergnano, Will Verling, Amit Verma, Rachel Vernall, Britney Vernon, Mark Vertue, Liberty Verueco, Jerik Verula, Natalie Vethanayagam, Shambavi Vettikumaran, Lucy Veys, Carinna Vickers, Saji Victor, SImpson Victoria, Celine Vidaillac, Jennifer Vidler, Bavithra Vijayakumar, Vinod W Vijayaraghavan Nalini, Brigita Vilcinskaite, Alicija Vileito, Neringa Vilimiene, Lynn Vinall, Sylvia Vinay, Latha Vinayakarao, Olivia Vincent, Rachel Vincent, Rosie Vincent, Pritpal Virdee, Emma Virgilio, Abdullah M Virk, Elisa Visentin, Martina Vitaglione, Karunakaran Vithian, Sorice Vittoria, Sankkita Vivekananthan, Elena Vlad, Ben Vlies, Linda von Oven, Claire Vooght, Kritchai Vutipongsatorn, Alain Vuylsteke, Eleftheria Vyras, Richard Wach, Beverley Wadams, Susan Wadd, Natalia Waddington, Phillip Wade, Jonathan Wadsley, Kirsten Wadsworth, Syed E I Wafa, Daniel Wagstaff, Lynda Wagstaff, Dalia Wahab, Zaroug Wahbi, Abiodun Waheed Adigun, Sawan Waidyanatha, Alicia Waite, Rachel Wake, Alice Wakefield, William Wakeford, Fiona Wakinshaw, Emma Waldeck, Andrew Walden, Lorna Walding, Alexandria Waldron, Joanne Waldron, Emma Wales, Batool Wali, Danielle Walker, Gemma Walker, Harriet Walker, Ian Walker, Kevin Walker, Kim Walker, Lauren Walker, Linda Walker, Lynne Walker, Olivia Walker, Rachel Walker, Rebecca Walker, Sara Walker, Susan Walker, Gillian Wallace, Rebecca Wallbutton, Jessica Wallen, Karl Wallendszus, Arabella Waller, Rosemary Waller, Gabiel Wallis, Gabriel Wallis, Louise Wallis, Matthew Wallis, Emma Walmsley, Donna Walsh, Elizabeth Walsh, Livia Walsh, Deborah Walstow, Daniel Walter, Alex Walters, Holt Walters, James Walters, Jocelyn Walters, Eileen Walton, Lucy Walton, Maisie Walton, Michael Walton, Olivia Walton, Sharon Walton, Susan Walton, Mandy Wan, Junita Wanda, Mary Wands, Rachel Wane, Frank Wang, Nick Wang, Ran Wang, Shentong Wang, Deborah Warbrick, Samantha Warburton, Christopher Ward, Deborah Ward, Emma Ward, Hannah Ward, Janice Ward, Joanna Ward, Luke Ward, Nicola Ward, Rachael Ward, Thomas Ward, Tom Ward, Scott A Warden, Gibril Wardere, Steve Wardle, Hassan Wardy, Gareth Waring, Scott Waring, Jenny Warmington, Ben Warner, Christian Warner, Lewis Warnock, Sarah Warran, Jade Warren, Lisa Warren, Rebecca Warren, Yolanda Warren, Davina Warrender, Hannah Warren-Miell, Adilia Warris, Gill Warwick, Helen Wassall, Elizabeth Wasson, Hazel Jane Watchorn, Holly Waterfall, Abby Waters, Donald Waters, Mark Waterstone, Abigail Watkin, Catherine Watkins, Catrin Watkins, Eleanor Watkins, Karen Watkins, Lynn Watkins, Abigail Watson, Adam J R Watson, Ekaterina Watson, Eleanor Watson, Felicity Watson, J G R Watson, Louisa Watson, Paul Watson, Rebecca Watson, Robert Watson, Kathleen Watson, Malcolm Watters, Donna Watterson, Khairiah Wattimena, Daniel Watts, John Watts, Merlin Watts, Victoria Waugh, Emma Wayman, Matthew Wayman, Akhlaq Wazir, Mark Weatherhead, Nick Weatherly, Chloe Webb, Claire Webb, Hayley Webb, Kathryn Webb, Kylie Webb, Stephen Webb, Cheryl Websdale, Deborah Webster, Ian Webster, Jemma Webster, Tim Webster, Jordan Wedlin, Ling Wee, Rebecca Weerakoon, Thanuja Weerasinghe, Janaka Weeratunga, Maria Weetman, Shuying Wei, Immo Weichert, Emma Welch, Hugh Welch, James Welch, Leanne Welch, Steven Welch, Benjamin Welham, Samantha Weller, Lucy Wellings, Brian Wells, Susan Wellstead, Berni Welsh, Richard Welsh, Ingeborg Welters, Rachael Welton, Victoria Wenn, Lauren Wentworth, Joan Wesonga, Kate Wesseldine, Joe West, Magdelena West, Raha West, Ruth West, Sophie West, Luke Western, Ruth Westhead, Heather Weston, Alice Westwood, Kelly Westwood, Sharon Westwood, Bill Wetherill, Sharon Wheaver, Helen Wheeler, Ben Whelan, Matthew Whelband, Amanda Whileman, Alison Whitcher, Andrew White, Ayesha White, Benjamin White, Catherine White, Christopher White, Duncan White, James White, Jonathan White, Katie White, Marie White, Nick White, Nikki White, Sarah White, Sonia White, Tracey White, Catherine Whitehead, Kelly Whitehorn, Anne Whitehouse, Claire Whitehouse, Tony Whitehouse, Julia Whiteley, Louiza Whiteley, Sophie Whiteley, Rachael Whitham, Gabriel Whitlingum, David Whitmore, Elizabeth Whittaker, Lindsay Whittam, Ashley Whittington, Helen Whittle, Robert Whittle, Eunice Wiafe, Lou Wiblin, Olivia Wickens, John Widdrington, Jason Wieboldt, Hannah Wieringa, Cornelia Wiesender, Laura Wiffen, Andrew Wight, Ashleigh Wignall, Christopher Wignall, Annamaria Wilce, Danielle Wilcock, Emma Wilcock, Louise Wilcox, Ben Wild, Laura Wild, Stephen Wild, Michael Wilde, Laura Wilding, Peter Wilding, Tracey Wildsmith, Joe Wileman, Joy Wiles, Kate Wiles, Elva Wilhelmsen, Thomas Wiliams, Janet Wilkie, David Wilkin, Hannah Wilkins, Joy Wilkins, Suzanne Wilkins, Iain Wilkinson, Lesley Wilkinson, Nicola Wilkinson, Sophia Wilkinson, Susan Wilkinson, Tim Wilkinson, Sylvia Willetts, Aimee Williams, Alexandra Williams, Alison Williams, Angharad Williams, Anna Williams, Ava Williams, Carl Williams, Caroline V Williams, Claire Williams, Dewi Williams, Eleri Williams, Gail Williams, Gemma Williams, Gina Williams, Hannah Williams, Heledd Williams, James Williams, Jennie Williams, Jill Williams, John Williams, Joseph Williams, Karen Williams, Kathryn Williams, Mandy Williams, Marie Williams, Mark Williams, Matthew Williams, Patricia Williams, Penny Williams, Rachel Williams, Robin Williams, Rupert Williams, Samson Williams, Sarah Williams, Sophie Williams, Steffan Williams, Tamanna Williams, Tania Williams, Annie Williamson, Cath Williamson, Catherine Williamson, Dawn Williamson, James D Williamson, Jodie Williamson, Rachel Williamson, Cath Williamson, Helen Williamson, Elaine Willis, Elizabeth Willis, Emily Willis, Heather Willis, Herika Willis, Joanna Willis, Louise Wills, Lucy Willsher, Catherine Willshire, Francesca Willson, Jayne Willson, Alison Wilson, Andrea Wilson, Anna Wilson, Antoinette Wilson, Billy Wilson, David Wilson, Imogen Wilson, James Wilson, Karen Wilson, Kate Wilson, Kristy-Ann Wilson, Lucinda Wilson, Mark Wilson, Stephen Wilson, Toni Wilson, John Wilson, Khin Lei Yee Win, Marlar Win, Than Win, Tin Tin Win, Wut Yee Win Win, Lucinda Winckworth, Laura Winder, Piers Winder, Stuart Winearl, Helen Winmill, Simon Winn, Carmen Winpenny, Helen Winslow, Helen Winter, Jonathan Winter, Barbara Winter-Goodwin, Joseph Winterton, Hannah Winwood, Jack Wischhusen, Stephen Wisdom, Matthew Wise, Martin Wiselka, Rebecca Wiseman, Sophie Wiseman, Steven Wishart, Tabitha WIshlade, Eric Witele, Nicholas Withers, Janet Wittes, Donna Wixted, Therese Wodehouse, Will Wolf, Nicola Wolff, Kirsten Wolffsohn, Rebecca Wolf-Roberts, Elena Wolodimeroff, Adam Wolstencroft, Alan Wong, Charlotte Wong, Chi-Hung Wong, Chi-Man Wong, Edwin Wong, Jessica S Y Wong, Kit Y Wong, Mei Yin Wong, Nick Wong, Sam Wong, Ting Wong, Amanda Wood, Caroline Wood, Dianne Wood, Fiona Wood, Gordon Wood, Hannah Wood, Jennifer Wood, Joe Wood, Lisa Wood, Louise Wood, Madeleine Wood, Stephen Wood, Tracy Wood, Katharine Woodall, Rebecca Woodfield, Christopher Woodford, Elizabeth Woodford, Jill Woodford, Louise Woodhead, Timothy Woodhead, Philip Woodland, Marc Woodman, Myles Woodman, Stephen Woodmansey, Charlotte Woods, Jane Woods, Katherine Woods, Sarah Woods, Zoe Woodward, Megan Woolcock, Gemma Wooldridge, Rebecca Woolf, Chris Woollard, Christopher Woollard, Louisa Woollen, Emma Woolley, Jade Woolley, Daniel Woosey, Dan Wootton, Joanne Wootton, Daniel Worley, Stephy Worton, Jonathan Wraight, Maria Wray, Kim Wren, Lynn Wren, Caroline Wrey Brown, Catherine Wright, Demi Wright, Francesca Wright, Holly Wright, Imogen Wright, Lianne Wright, Rachel Wright, Sarah Wright, Stephanie Wright, Tim Wright, Caroline Wroe, Hannah Wroe, Henry Wu, Peishan Wu, Pensee Wu, Jonathan Wubetu, Fitria Wulandari, Retno Wulandari, Subie Wurie, Craig Wyatt, Frederick Wyn-Griffiths, Inez Wynter, Bindhu Xavier, Arnold Xhikola, Bingru E Xia, Zhongyang Xia, Effua Yacoba, Masseh Yakubi, May Yan, Yuuki Yanagisawa, Freda Yang, Yingjia Yang, Michael Yanney, Woei Lin Yap, Nabil Yaqoob, Salima Yasmin, Bryan Yates, David Yates, Edward Yates, Helen Yates, Thomas Yates, Mark Yates, Jessica Ye, Charlotte Yearwood Martin, Khin Yein, Fiona Yelnoorkar, Alastair Yeoh, Chun Yu Yeung, Peter Yew, Durgesh Yewatkar, Laura B Ylquimiche Melly, Inez Ynter, H Yong, Jemma Yorke, Jasmine Youens, Abdel Younes Ibrahim, Eoin Young, Gail Young, Louise Young, Asfand Yousafzar, Sajeda Youssouf, Ahmed Yousuf, Chrissie Yu, Jack S J Yuan, Nindya Yufaniaputri, Bernard Yung, Daniel Yusef, Said Yusef, Intekhab Yusuf, Anna-Sophia Zafar, Silvia Zagalo, Su Zaher, Aqsa Zahoor, Mahrukh Zainab, Thornton Zak, Kareem Zaki, Nabhan Zakir, Kasia Zalewska, Ane Zamalloa, Mohsin Zaman, Shakir Zaman, Julie Zamikula, Louise Zammit, Marie Zammit-Mangion, Marcelina Zawadzka, Mohammed Zayed, Esther Zebracki, Daniel Zehnder, Lisa Zeidan, Darius Zeinali, Jing Zhang, Xiaobei Zhao, Dongling Zheng, Doreen Zhu, Madiha Zia, Omar Zibdeh, Rabia Zill-E-Huma, Ei Thankt Zin, Eva Zincone, Garikai Zindoga, Eleanor Zinkin, Vivian Zinyemba, Christos Zipitis, Letizia Zitter, Arkadiusz Zmierczak, Garazi Zubikarai, Azam Zubir, Naz Zuhra, Rasha Zulaikha, Sabrina Zulfikar, Carol Zullo, Ana Zuriaga-Alvaro

## Abstract

**Background:**

We aimed to evaluate the use of baricitinib, a Janus kinase (JAK) 1–2 inhibitor, for the treatment of patients admitted to hospital with COVID-19.

**Methods:**

This randomised, controlled, open-label, platform trial (Randomised Evaluation of COVID-19 Therapy [RECOVERY]), is assessing multiple possible treatments in patients hospitalised with COVID-19 in the UK. Eligible and consenting patients were randomly allocated (1:1) to either usual standard of care alone (usual care group) or usual care plus baricitinib 4 mg once daily by mouth for 10 days or until discharge if sooner (baricitinib group). The primary outcome was 28-day mortality assessed in the intention-to-treat population. A meta-analysis was done, which included the results from the RECOVERY trial and all previous randomised controlled trials of baricitinib or other JAK inhibitor in patients hospitalised with COVID-19. The RECOVERY trial is registered with ISRCTN (50189673) and ClinicalTrials.gov (NCT04381936) and is ongoing.

**Findings:**

Between Feb 2 and Dec 29, 2021, from 10 852 enrolled, 8156 patients were randomly allocated to receive usual care plus baricitinib versus usual care alone. At randomisation, 95% of patients were receiving corticosteroids and 23% were receiving tocilizumab (with planned use within the next 24 h recorded for a further 9%). Overall, 514 (12%) of 4148 patients allocated to baricitinib versus 546 (14%) of 4008 patients allocated to usual care died within 28 days (age-adjusted rate ratio 0·87; 95% CI 0·77–0·99; p=0·028). This 13% proportional reduction in mortality was somewhat smaller than that seen in a meta-analysis of eight previous trials of a JAK inhibitor (involving 3732 patients and 425 deaths), in which allocation to a JAK inhibitor was associated with a 43% proportional reduction in mortality (rate ratio 0·57; 95% CI 0·45–0·72). Including the results from RECOVERY in an updated meta-analysis of all nine completed trials (involving 11 888 randomly assigned patients and 1485 deaths) allocation to baricitinib or another JAK inhibitor was associated with a 20% proportional reduction in mortality (rate ratio 0·80; 95% CI 0·72–0·89; p<0·0001). In RECOVERY, there was no significant excess in death or infection due to non-COVID-19 causes and no significant excess of thrombosis, or other safety outcomes.

**Interpretation:**

In patients hospitalised with COVID-19, baricitinib significantly reduced the risk of death but the size of benefit was somewhat smaller than that suggested by previous trials. The total randomised evidence to date suggests that JAK inhibitors (chiefly baricitinib) reduce mortality in patients hospitalised for COVID-19 by about one-fifth.

**Funding:**

UK Research and Innovation (Medical Research Council) and National Institute of Health Research.

## Introduction

In patients admitted to hospital with severe COVID-19, the host immune response is thought to play a key role in driving an acute inflammatory process resulting in hypoxic respiratory failure that might require mechanical ventilator support or lead to death.^1^, ^2^ It has previously been shown that the use of dexamethasone and other corticosteroids reduces the risk of death in patients with severe hypoxic COVID-19 and that the addition of an interleukin-6 (IL-6) receptor blocker further reduces the risk of death in these patients.^3^, ^4^, ^5^, ^6^

Baricitinib is an inhibitor of Janus kinase (JAK)1 and JAK2, which is licensed in the UK for the treatment of rheumatoid arthritis and atopic dermatitis. The JAKs are a family of four transmembrane protein kinases (JAK1, JAK2, JAK3, and tyrosine kinase 2 [TYK2]) that mediate intracellular signalling of a range of extracellular cytokines and interferons.^7^ JAK inhibition prevents downstream phosphorylation and hence activation of signal transducers and activators of transcription (STAT). Since the JAK-STAT pathway mediates the effect of several cytokines, including IL-6, which are raised in severe COVID-19, JAK inhibitors have been proposed as a potential therapeutic option for severe COVID-19.^8^, ^9^ Baricitinib also has moderate inhibitory activity against TYK2 and genetic data support a causal link between high TYK2 expression and life-threatening COVID-19.^10^ Baricitinib was also predicted, by use of artificial intelligence, to reduce endocytosis of SARS-CoV-2 into lung cells by inhibiting AP2-associated protein kinase 1 and cyclin G associated kinase.^8^

**Research in context**

**Evidence before this study**

We searched MEDLINE, Embase, MedRxiv and the WHO International Clinical Trials Registry Platform for trials published between Sept 1, 2019, and Feb 13, 2022, for randomised controlled trials evaluating the effect of baricitinib or another Janus kinase (JAK) inhibitor in patients hospitalised with COVID-19 using the search terms (“SARS-CoV-2.mp” OR “SARS-CoV2” OR “SARSCoV2.mp” OR “COVID.mp” OR “COVID-19.mp” OR “COVID19.mp” OR “2019-nCoV.mp” OR “Coronavirus.mp” or “Coronavirinae/”) AND (“JAK inhibitor.mp or Janus kinase inhibitor/” OR “Janus kinase inhibitor.mp” OR “Baricitinib.mp or baricitinib/” OR terms for other specific JAK inhibitors (listed in the appendix p 28) and using validated filters to select for randomised controlled trials. No language restrictions were applied.

We identified eight relevant randomised trials with results available that assessed JAK inhibitors in patients hospitalised with COVID-19: three assessed baricitinib, three assessed ruxolitinib, and two assessed tofacitinib. Six of the trials had been fully published of which four were considered to have low risk of bias for the 28-day mortality outcome with two having some concerns (one because of lack of information about prespecified analyses and some imbalances between randomised groups of other interventions given during the trial; the other because of lack of information about the randomisation process, inconsistency in reporting of outcome endpoint timing, and lack of information about prespecified analyses). A meta-analysis of these eight trials, which included a total of 425 deaths among 3732 patients, suggested that allocation to a JAK inhibitor was associated with a 43% proportional reduction in 28-day mortality (rate ratio 0·57 [95% CI 0·45–0·72]).

**Added value of this study**

The Randomised Evaluation of COVID-19 Therapy (RECOVERY) trial is the largest randomised trial of the effect of a JAK inhibitor in patients hospitalised with COVID-19. We found that in 8156 patients admitted to hospital with COVID-19, baricitinib reduced 28-day mortality by 13%, increased the probability of discharge alive within 28 days, and, among patients who were not receiving invasive mechanical ventilation at randomisation, reduced the probability of progression to the composite outcome of invasive mechanical ventilation or death. The benefits were consistent in all subgroups of patients, including those receiving a systemic corticosteroid or an interleukin-6 (IL-6) receptor blocker.

**Implications of all the available evidence**

The randomised evidence from all nine completed JAK inhibitor trials to date suggest that treatment with baricitinib or an alternative JAK inhibitor reduces mortality by about one-fifth (rate ratio 0·80 [95% CI 0·72–0·89]) in patients hospitalised with COVID-19, including those already receiving a systemic corticosteroid or an IL-6 receptor blocker.

Baricitinib was tested in combination with remdesivir in the Adaptive Covid-19 Treatment Trial-2 (ACTT-2) and was shown to improve time to recovery compared with remdesivir alone (rate ratio for recovery 1·16, 95% CI 1·01–1·32). There was also a suggestion that 28-day mortality might be reduced by baricitinib (hazard ratio 0·65, 95% CI 0·39–1·09).^11^ As a consequence, the US Food and Drug Administration (FDA) issued an emergency use authorisation for the use of baricitinib for the treatment of COVID-19 in patients who are hospitalised and requiring oxygen, invasive mechanical ventilation, or extracorporeal membrane oxygenation.^12^ Since then, a further seven randomised trials of JAK inhibitors have reported (NCT04362137, NCT04377620 13, 14, 15, 16, 17), of which two have reported a significant reduction in mortality (NCT04377620^16^). Here we report the results of a large randomised controlled trial of baricitinib in patients hospitalised with severe COVID-19.

## Methods

### Study design and participants

The Randomised Evaluation of COVID-19 Therapy (RECOVERY) trial is an investigator-initiated, individually randomised, controlled, open-label, platform trial to evaluate the effects of potential treatments in patients hospitalised with COVID-19. Details of the trial design and results for other possible treatments (dexamethasone, hydroxychloroquine, lopinavir–ritonavir, azithromycin, tocilizumab, convalescent plasma, colchicine, aspirin, and casirivimab plus imdevimab) have been published previously.^3^, ^5^, ^18^, ^19^, ^20^, ^21^, ^22^, ^23^, ^24^ The trial is underway at 177 hospital organisations in the UK supported by the National Institute for Health Research Clinical Research Network (appendix pp 3–27). Of these, 159 UK hospitals enrolled participants in the evaluation of baricitinib. The trial is coordinated by the Nuffield Department of Population Health at the University of Oxford (Oxford, UK), the trial sponsor. The trial is done in accordance with the principles of the International Conference on Harmonisation–Good Clinical Practice guidelines and approved by the UK Medicines and Healthcare products Regulatory Agency and the Cambridge East Research Ethics Committee (reference 20/EE/0101). The protocol and statistical analysis plan are available in the appendix (pp 68–145) with additional information available on the study website.

Patients aged at least 2 years admitted to hospital were eligible for the study if they had clinically suspected or laboratory confirmed SARS-CoV-2 infection and no medical history that might, in the opinion of the attending clinician, put the patient at substantial risk if they were to participate in the trial. Patients were ineligible for the comparison of baricitinib versus usual care if younger than 2 years, had estimated glomerular filtration rate (eGFR) of less than 15 mL/min per 1·73 m^2^ or were on dialysis or haemofiltration, had a neutrophil count of less than 0·5 × 10^9^ per L, had evidence of active tuberculosis infection, or were pregnant or breastfeeding. Written informed consent was obtained from all patients, or a legal representative if patients were too unwell or unable to provide consent.

### Randomisation and masking

Baseline data were collected by means of a web-based case report form that included demographics, amount of respiratory support, major comorbidities, suitability of the study treatment for a particular patient, SARS-CoV-2 vaccination status, and treatment availability at the study site (appendix pp 38–42).

Eligible and consenting patients were assigned in a 1:1 ratio to either usual standard of care plus baricitinib or usual standard of care alone, by means of web-based simple (unstratified) randomisation with allocation concealed until after randomisation (appendix pp 33–37). For some patients, baricitinib was unavailable at the hospital at the time of enrolment or was considered by the managing physician to be either definitely indicated or definitely contraindicated. These patients were excluded from the randomised comparison between baricitinib versus usual care. Patients allocated to baricitinib were to receive baricitinib 4 mg daily for 10 days (or until discharge if sooner). The dose was to be reduced for patients with eGFR of less than 60 mL/min per 1·73 m^2^ or receiving probenecid, and for children younger than 9 years (see appendix p 30 for dosing details). Previous or subsequent administration of tocilizumab was permitted at the discretion of the managing physician who was also responsible for considering the risk of infection and gastrointestinal perforation (particularly in the context of corticosteroid use).

As a platform trial, and in a factorial design, patients could be simultaneously randomised to other treatment groups: colchicine versus usual care, aspirin versus usual care, dimethyl fumarate versus usual care, casirivimab plus imdevimab versus usual care, and empagliflozin versus usual care. Further details of when these factorial randomisations were open are provided in the appendix (pp 38–39). Participants and local study staff were not masked to the allocated treatment. The Trial Steering Committee, investigators, and all other individuals involved in the trial were masked to outcome data during the trial.

### Procedures

An online follow-up form was completed by site staff when patients were discharged, had died, or at 28 days after randomisation, whichever occurred first (appendix pp 45–50). Information was recorded on adherence to allocated trial treatment, receipt of other COVID-19 treatments, duration of admission, receipt of respiratory or renal support, new cardiac arrhythmia, thrombosis, clinically significant bleeding, non-COVID-19 infection, and vital status (including cause of death). In addition, routinely collected health-care and registry data were obtained, including information on vital status at day 28 (with date and cause of death); discharge from hospital; and receipt of respiratory support or renal replacement therapy.

### Outcomes

Outcomes were assessed at 28 days after randomisation, with further analyses specified at 6 months. The primary outcome was 28-day all-cause mortality. Secondary outcomes were time to discharge from hospital, and, among patients not on invasive mechanical ventilation at randomisation, the composite outcome of invasive mechanical ventilation (including extra-corporeal membrane oxygenation) or death. Prespecified subsidiary clinical outcomes were use of invasive or non-invasive ventilation among patients not on any ventilation at randomisation, time to successful cessation of invasive mechanical ventilation (defined as cessation of invasive mechanical ventilation within, and survival to, 28 days), and use of renal dialysis or haemofiltration. Prespecified safety outcomes were cause-specific mortality, major cardiac arrhythmia, thrombotic and major bleeding events, and other infections. Information on suspected serious adverse reactions was collected in an expedited fashion to comply with regulatory requirements. Details of the methods used to ascertain and derive outcomes are provided in the appendix (pp 146–66).

### Statistical analysis

For all outcomes, intention-to-treat analyses compared patients randomly assigned to baricitinib with patients randomly assigned to usual care. Through the play of chance in the unstratified randomisation, patients in the baricitinib group were slightly older than patients in the usual care group (table 1). In accordance with the prespecified statistical analysis plan for dealing with baseline imbalances in important prognostic factors (appendix p 130), estimates of the effect of allocation to baricitinib on major outcomes were adjusted for age in three groups (<70 years, ≥70 to <80 years, and ≥80 years). Sensitivity analyses were done without this adjustment and, separately, with further adjustment for other predefined subgroups of interest.

**Table 1:** Baseline characteristics

		**Baricitinib group (n=4148)**	**Usual care group (n=4008)**
Age, years		58·5 (15·4)	57·7 (15·5)
	<70	3142 (76%)	3086 (77%)
	≥70 to <80	665 (16%)	655 (16%)
	≥80	341 (8%)	267 (7%)
Sex			
	Male	2740 (66%)	2638 (66%)
	Female	1408 (34%)	1370 (34%)
Ethnicity			
	White	3323 (80%)	3203 (80%)
	Black, Asian, and minority ethnic	457 (11%)	469 (12%)
	Unknown	368 (9%)	336 (8%)
Time since symptom onset, days		9 (6–12)	9 (6–11)
Time since admission to hospital, days		1 (1–3)	1 (1–3)
Respiratory support received			
	None	228 (5%)	237 (6%)
	Simple oxygen	2770 (67%)	2743 (68%)
	Non-invasive ventilation	1016 (24%)	911 (23%)
	Invasive mechanical ventilation	134 (3%)	117 (3%)
Laboratory measurements			
	C-reactive protein, mg/L	84 (42–146)	87 (44–143)
	Creatinine, μmol/L	76 (63–93)	77 (63–94)
Previous diseases			
	Diabetes	961 (23%)	941 (23%)
	Heart disease	782 (19%)	706 (18%)
	Chronic lung disease	882 (21%)	783 (20%)
	Tuberculosis	0	0
	HIV	13 (<1%)	9 (<1%)
	Severe liver disease*	33 (1%)	33 (1%)
	Severe kidney impairment†	101 (2%)	79 (2%)
	Any of the above	1957 (47%)	1834 (46%)
SARS-CoV-2 PCR test result			
	Positive	3969 (96%)	3873 (97%)
	Negative	43 (1%)	32 (1%)
	Unknown	136 (3%)	103 (3%)
	Received a COVID-19 vaccine	1755 (42%)	1665 (42%)
Use of other treatments			
	Corticosteroids	3962 (96%)	3809 (95%)
	Remdesivir	878 (21%)	789 (20%)
	Tocilizumab	951 (23%)	921 (23%)
	Plan to use tocilizumab within the next 24 h	391 (9%)	365 (9%)
Other randomly assigned treatments			
	Colchicine	401 (10%)	401 (10%)
	Aspirin	462 (11%)	453 (11%)
	Casirivimab–imdevimab	440 (11%)	449 (11%)

Data are mean (SD), n (%), or median (IQR). 33 children and 4 post-partum women were randomly assigned.

* Defined as requiring ongoing specialist care.

† Defined as estimated glomerular filtration rate <30 mL/min per 1·73 m^2^.

For the primary outcome of 28-day mortality, the hazard ratio from an age-adjusted Cox model was used to estimate the mortality rate ratio. We constructed Kaplan-Meier survival curves to display cumulative mortality over the 28-day period. We used the same method to analyse time to hospital discharge and successful cessation of invasive mechanical ventilation, with patients who died in hospital right-censored on day 29. Median time to discharge was derived from Kaplan-Meier estimates. For the prespecified composite secondary outcome of progression to invasive mechanical ventilation or death within 28 days (among those not receiving invasive mechanical ventilation at randomisation), and the subsidiary clinical outcomes of receipt of ventilation and use of haemodialysis or haemofiltration, the precise dates were not available and so a log-binomial regression model was used to estimate the age-adjusted risk ratio. Estimates of rate and risk ratios (both denoted RR) are shown with 95% CIs.

Prespecified analyses of the primary outcome were done in subgroups defined by six characteristics at the time of randomisation (age, sex, ethnicity, days since symptom onset, amount of respiratory support, and use of corticosteroids) with tests of heterogeneity or trend, as appropriate. The full database is held by the study team which collected the data from study sites and did the analyses at the Nuffield Department of Population Health, University of Oxford.

The independent data monitoring committee reviewed unmasked analyses of the study data and any other information considered relevant to the trial at intervals of around 2 to 4 weeks (depending on speed of enrolment) and was charged with establishing whether, in their view, the randomised comparisons in the study provided evidence on mortality that was strong enough (with a range of uncertainty around the results that was narrow enough) to affect national and global treatment strategies (appendix p 51).

As stated in the protocol, appropriate sample sizes could not be estimated when the trial was being planned. On the advice of the trial steering committee, recruitment to this comparison was closed on Dec 29, 2021 when over 8150 patients had been randomly assigned and the masked 28-day mortality rate was 12·9% (suggesting there would be at least 1050 deaths), giving at least 90% power to detect a proportional risk reduction in the primary outcome of one-fifth at a two-sided significance level of 1%. The trial steering committee and all other individuals involved in the trial were masked to outcome data until after the close of recruitment.

For the primary outcome of 28-day mortality, the results from the RECOVERY trial were subsequently included in a meta-analysis of results from all previous randomised controlled trials of a JAK inhibitor for patients hospitalised with COVID-19. Details of the systematic search methods are provided in the appendix (pp 30–32). For each trial, we compared the observed number of deaths among patients allocated to the JAK inhibitor with the expected number if all patients were at equal risk (ie, we calculated the observed minus expected statistic [o–e], and its variance v). For RECOVERY, these were estimated from the age-adjusted mortality log rate ratio and its standard error but for other trials, where the exact timing of each death was not available, these were calculated from standard formulae for 2 × 2 contingency tables. We then combined trial results using the log of the mortality rate ratio calculated as the inverse-variance weighted average S/V with variance 1/V (and hence with 95% CI S/V ±1·96/√V), where S is the sum over all trials of (o–e) and V is the sum over all trials of v.^25^ Such meta-analyses do not make any assumptions about the nature of any true heterogeneity in the log of the mortality rate ratio between different trials (in particular it does not assume that it is zero). Analyses were done by means of SAS version 9.4 and R version 4.0.3. The trial is registered with ISRCTN (50189673) and ClinicalTrials.gov (NCT04381936).

### Role of the funding source

The funders of the study had no role in study design, data collection, data analysis, data interpretation, or writing of the report.

## Results

**Figure 1:**
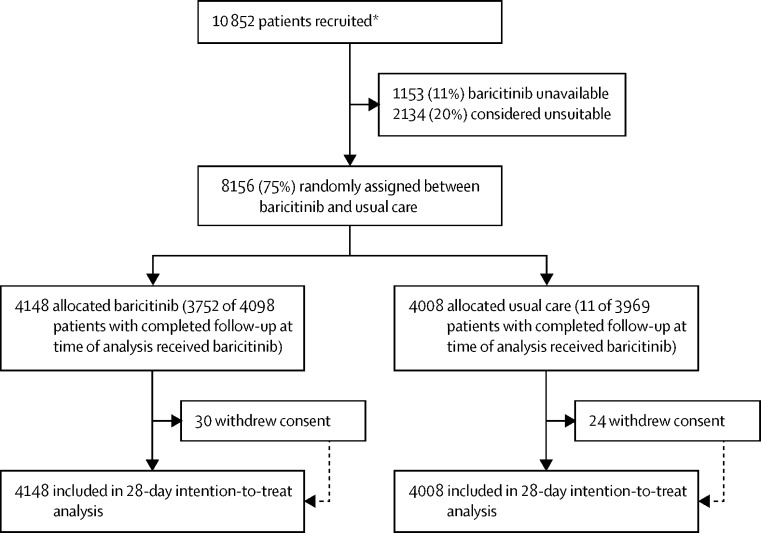
Trial profile Baricitinib unavailable and baricitinib considered unsuitable are not mutually exclusive. *Number recruited overall during period that adult participants could be recruited into the baricitinib comparison.

Between Feb 2 and Dec 29, 2021, 8156 (75%) of 10 852 patients enrolled into the RECOVERY trial were eligible to be randomly allocated to baricitinib (ie, the treatment was available in the hospital at the time and the attending clinician was of the opinion that the patient had no known indication for or contraindication to it, figure 1). 4148 patients were randomly allocated to baricitinib and 4008 were randomly allocated to usual care. The mean age of study participants in this comparison was 58·1 years (SD 15·5) with a chance imbalance whereby patients randomly allocated to baricitinib were, on average, 0·8 years older than those allocated to the usual care group (table 1). At randomisation, 7771 (95%) patients were receiving corticosteroids and 1872 (23%) were receiving tocilizumab (with planned use within the next 24 h recorded for a further 756 [9%]; table 1, appendix p 53). About two-thirds were receiving simple oxygen and one-quarter were receiving non-invasive ventilation, with small numbers receiving invasive mechanical ventilation or no respiratory support at all. 3420 (42%) patients had received at least one dose of a SARS-CoV-2 vaccine.

The follow-up form was completed for 4098 (99%) patients in the baricitinib group and 3969 (99%) patients in the usual care group. Among patients with a completed follow-up form, 92% allocated to baricitinib were reported to have received the treatment compared with less than 1% allocated to usual care (figure 1, appendix p 54). Use of other treatments for COVID-19 was broadly similar among patients allocated to baricitinib and among those allocated to usual care, with nine-tenths receiving a corticosteroid, one-fifth receiving remdesivir, and one-tenth receiving casirivimab plus imdevimab, although use of tocilizumab during the follow-up period was slightly lower in the baricitinib group than in the usual care group (26% *vs* 29%; appendix p 54).

**Figure 2:**
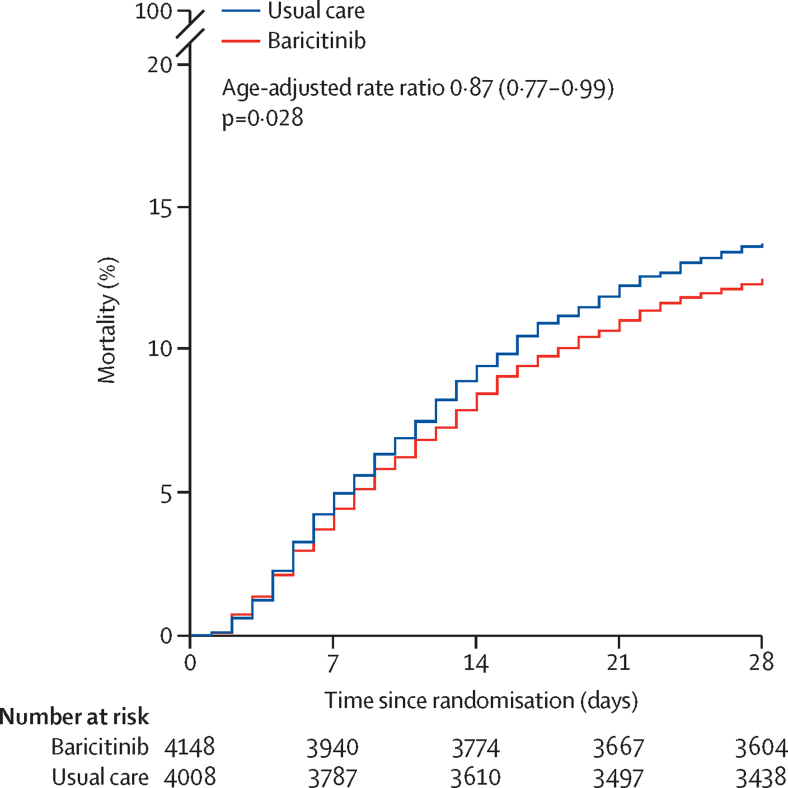
Effect of allocation to baricitinib on 28-day mortality

**Figure 3:**
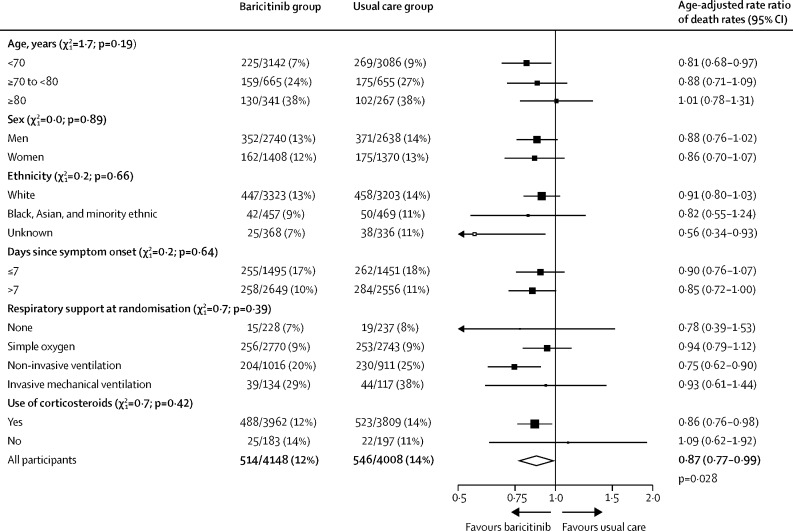
Effect of allocation to baricitinib on 28-day mortality by baseline characteristics Subgroup-specific rate ratio estimates are represented by squares (with areas of the squares proportional to the amount of statistical information) and the lines through them correspond to the 95% CIs. The days since onset and use of corticosteroids subgroups exclude those with missing data, but these patients are included in the overall summary diamond.

Primary and secondary outcome data are known for more than 99% of randomly assigned patients. Allocation to baricitinib was associated with a significant reduction in the primary outcome of 28-day mortality compared with usual care alone: 514 (12%) of 4148 patients in the baricitinib group died *vs* 546 (14%) of 4008 patients in the usual care group (age-adjusted rate ratio 0·87; 95% CI 0·77–0·99; p=0·028; table 2, figure 2). Similar proportional risk reductions were seen in sensitivity analyses adjusted for all prespecified subgroups (as listed in figure 3) and without adjustment for the 0·8-year age imbalance between randomised groups (appendix p 55), and when restricted to participants with a positive SARS-CoV-2 PCR test (age-adjusted rate ratio 0·90, 0·80–1·02).

**Table 2:** Effect of allocation to baricitinib on key study outcomes

		**Treatment allocation**		**RR (95% CI)**	**p value**
		Baricitinib (n=4148)	Usual care (n=4008)		
**Primary outcome**					
28-day mortality		514 (12%)	546 (14%)	0·87 (0·77–0·99)	0·028
**Secondary outcomes**					
Time to being discharged alive, days		8 (5–17)	8 (5–20)	..	..
Discharged from hospital within 28 days		3338 (80%)	3136 (78%)	1·10 (1·04–1·15)	0·0002
Receipt of invasive mechanical ventilation or death*		633/4014 (16%)	678/3891 (17%)	0·89 (0·81–0·98)	0·016
	Invasive mechanical ventilation	287/4014 (7%)	333/3891 (9%)	0·85 (0·73–0·99)	0·033
	Death	475/4014 (12%)	502/3891 (13%)	0·89 (0·80–1·00)	0·049
**Subsidiary clinical outcomes**					
Receipt of ventilation†		595/2998 (20%)	638/2980 (21%)	0·93 (0·84–1·03)	0·16
	Non-invasive ventilation	587/2998 (20%)	623/2980 (21%)	0·94 (0·85–1·04)	0·23
	Invasive mechanical ventilation	131/2998 (4%)	149/2980 (5%)	0·89 (0·71–1·12)	0·32
Successful cessation of invasive mechanical ventilation‡		61/134 (46%)	43/117 (37%)	1·28 (0·87–1·90)	0·21
Use of haemodialysis or haemofiltration§		87/4139 (2%)	110/4003 (3%)	0·78 (0·59–1·03)	0·08

Data are n (%), n/N (%), or median (IQR). RR=rate ratio for the outcomes of 28-day mortality, hospital discharge, and successful cessation of invasive mechanical ventilation, and risk ratio for other outcomes. Estimates of the RR and its 95% CI are adjusted for age in three categories (<70 years, 70–79 years, and 80 years or older).

* Analyses exclude those on invasive mechanical ventilation at randomisation.

† Analyses exclude those on any form of ventilation at randomisation.

‡ Analyses restricted to those on invasive mechanical ventilation at randomisation.

§ Analyses exclude those on haemodialysis or haemofiltration at randomisation.

The proportional effect of baricitinib on mortality was consistent across all six prespecified subgroups (all interaction p values >0·18; figure 3), including by amount of respiratory support received and use of dexamethasone at randomisation and, in five exploratory subgroups, including by use of tocilizumab or remdesivir at baseline (all interaction p values >0·10; appendix p 64). There was no evidence that the effect of baricitinib on mortality varied depending on concurrent randomised allocation to colchicine, aspirin, or casirivimab–imdevimab (all interaction p values >0·32).

Discharge alive within 28 days was more common among those allocated to baricitinib compared with usual care (80% *vs* 78%; age-adjusted rate ratio 1·10, 95% CI 1·04–1·15; median 8 days [IQR 5–17] *vs* 8 days [IQR 5–20]; table 2). Among patients not on invasive mechanical ventilation at baseline, allocation to baricitinib was associated with a lower risk of progressing to the composite secondary outcome of invasive mechanical ventilation or death (16% *vs* 17%, age-adjusted risk ratio 0·89, 0·81–0·98; table 2 and appendix p 65). The proportional effects of baricitinib versus usual care on these secondary outcomes were similar across all prespecified subgroups (appendix pp 65–66). Results for the 33 children included in this comparison are shown in the appendix (p 56).

**Figure 4:**
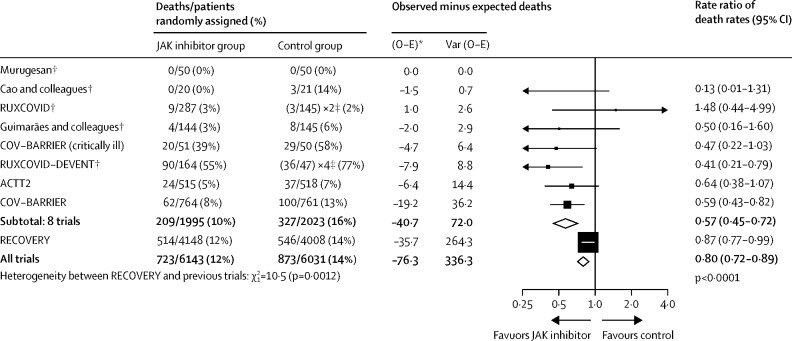
Meta-analysis of mortality in randomised controlled trials of a JAK inhibitor in patients hospitalised with COVID-19 JAK=Janus kinase. O–E=observed–expected. Var=variance. RR=mortality rate ratio. Details of the individual studies, including the use of placebo or other treatments in the control group are shown in the appendix (p 62). *For RECOVERY, the O–E and and its variance are calculated from the age-adjusted log RR and its standard error. For the other trials, the O–E statistics and their variances are calculated from 2 × 2 tables. Rate ratio is calculated by taking ln rate ratio to be (O–E)/V with normal variance 1/V, where V=Var (O–E). Subtotals or totals of (O–E) and of V yield inverse-variance weighted averages of the ln rate ratio values. †These trials assessed a JAK inhibitor other than baricitinib. If the meta-analysis was restricted to RECOVERY plus the three other trials of baricitinib, the RR would be 0·81 (95% CI 0·73–0·91). ‡For balance, controls in the n:1 studies count n times in the control totals and subtotals, but only count once when calculating their O–E or V values.

There were no significant differences in the prespecified subsidiary clinical outcomes of cause-specific mortality other than a reduction in death due to COVID-19 (appendix p 57) or in use of ventilation, successful cessation of invasive mechanical ventilation, or receipt of haemodialysis or haemofiltration (table 2). There were no significant differences in the rates of non-SARS-CoV-2 infection, thrombotic events, or clinically significant bleeding, but allocation to baricitinib was associated with a nominally significant reduction in new onset cardiac arrythmia (2·3% *vs* 3·2%, p=0·017; appendix p 58). In exploratory analyses, allocation to baricitinib versus usual care was not associated with any significant differences in non-COVID-19 causes of death or infection among those recorded as having been treated with tocilizumab at baseline (appendix pp 59–60). There were 12 reports of a serious adverse reaction believed to be related to treatment with baricitinib (appendix p 61), including four participants with a serious non-SARS-CoV-2 infection, three with a bowel perforation, and two with a pulmonary embolism. Our systematic search identified eight previous trials of a JAK inhibitor for the treatment of patients hospitalised with COVID-19, involving a total of 3732 randomly assigned patients and 425 deaths (figure 4, appendix p 62).^11^, ^13^, ^14^, ^15^, ^16^, ^17^, ^18^, ^19^ In these eight trials, allocation to a JAK inhibitor was associated with a significant 43% proportional reduction in mortality (rate ratio 0·57; 95% CI 0·45–0·72). This was significantly greater than the mortality risk reduction seen in RECOVERY (test for heterogeneity, p=0·0012). After inclusion of the results from RECOVERY into this meta-analysis, the average mortality rate ratio from all nine trials, now involving 11 888 randomised patients and 1485 deaths, was 0·80 (0·72–0·89; p<0·0001).

## Discussion

In this large, randomised trial, allocation to baricitinib significantly reduced 28-day mortality by about one-eighth. This is somewhat less than had been suggested by eight previous randomised controlled trials of a JAK inhibitor which, together, suggested that allocation to a JAK inhibitor in patients with COVID-19 reduces 28-day mortality by about two-fifths. RECOVERY was more than three times the size (in terms of statistical information) of these eight previous trials put together. When combined in an updated meta-analysis, allocation to baricitinib or another JAK inhibitor in these nine trials was associated with a significant reduction in 28-day mortality of one-fifth. Although not as large as perhaps previously thought, this still represents an important reduction in mortality risk for patients hospitalised because of COVID-19.

Strengths of the RECOVERY trial included that it was randomised, had a large sample size, had broad eligibility criteria, and more than 99% of patients were followed up for the primary outcome. The study has some limitations: this randomised trial is open label (ie, participants and local hospital staff are aware of the assigned treatment). However, the outcomes are unambiguous and were ascertained without bias through linkage to routine health records. Use of tocilizumab during the follow-up period was slightly lower among those allocated to baricitinib compared with control (26% *vs* 29%). Based on what we already know about the effects of tocilizumab, this would, if anything, lead to a small underestimate of the effects of baricitinib. Furthermore, use of anti-viral or immunomodulatory treatments known to reduce mortality in this setting was similar in those allocated to baricitinib and those allocated to usual care. Information on radiological, virological, or physiological outcomes was not collected. This evaluation of baricitinib was done only in the UK, with low rates of HIV among trial participants (<1%) and no participants with active tuberculosis, since active tuberculosis was a contraindication to inclusion in the baricitinib comparison. The effect of baricitinib on non-SARS-CoV-2 infections might be different in populations with a higher prevalence of tuberculosis or HIV.

The smaller effect size observed in RECOVERY compared with earlier trials of baricitinib might simply be a chance effect. However, several other factors could have contributed. The patient population in RECOVERY might have been broader than some of the other trials, which might have been enriched for patients more likely to benefit from immunomodulatory therapy. The use of concomitant therapies has varied between the trials. For example, the ACTT-2 trial^11^ did not permit the use of dexamethasone as a treatment for COVID-19, and ACTT-2, COV-BARRIER,^16^ RUXCOVID, and the study by Guimarães and colleagues^15^ all excluded the use of an IL-6 receptor blocker (NCT04362137). Other factors that might be different between the trials include the prevalence of SARS-CoV-2 vaccination and the predominant circulating SARS-CoV-2 variant. However, there is no clear reason to believe that, among patients admitted to hospital with severe COVID-19 requiring oxygen or ventilatory support, the proportional risk reduction in mortality with baricitinib, a host-directed therapy, would differ by vaccination status or SARS-CoV-2 variant—and we found no evidence of this. Despite the heterogeneity of effect between RECOVERY and the previous eight trials combined, the overall result of the meta-analysis (which makes no assumptions about the nature of any true differences in treatment effects between the different populations studied) provides the best guide of the proportional benefits that might be expected from the use of baricitinib in clinical practice. The size of the RECOVERY trial allows exploration of the effects of treatment among different subgroups of patients. The benefits of baricitinib on 28-day mortality were consistent across all subgroups, including by age, sex, ethnicity, and amount of respiratory support received (although over 90% of participants were either on simple oxygen or receiving non-invasive mechanical ventilation). The benefits of baricitinib were also consistent regardless of concomitant treatment with remdesivir, a systemic corticosteroid or an IL-6 receptor blocker (tocilizumab or sarilumab), or previous receipt of a SARS-CoV-2 vaccine. Reassuringly, we found no evidence that allocation to baricitinib was associated with excess rates of non-COVID-19 mortality, non-SARS-CoV-2 infection, or thrombosis by comparison with usual care.

On Nov 19, 2020, the FDA granted emergency use authorisation for baricitinib in combination with remdesivir, which was revised on July 28, 2021 to no longer require co-administration with remdesivir. On May 10, 2022 the FDA issued a new indication for the use of baricitinib in adults hospitalised with COVID-19 requiring supplemental oxygen, non-invasive or invasive mechanical ventilation, or extracorporeal membrane oxygenation.^12^, ^26^ US National Institutes of Health guidelines updated in February, 2022 recommend the use of baricitinib for patients on dexamethasone who have rapidly increasing oxygen needs and systemic inflammation.^27^ In January, 2022, the World Health Organization updated their COVID-19 therapeutics guidelines to include a strong recommendation for the use of baricitinib as an alternative to an IL-6 receptor blocker, in combination with corticosteroids, in patients with severe or critical COVID-19.^28^ The results from the RECOVERY trial and our meta-analysis considerably strengthen the evidence that baricitinib can reduce mortality and other adverse clinical outcomes in patients hospitalised with COVID-19 and support the co-administration of baricitinib with dexamethasone or an IL-6 receptor blocker.

In summary, this large, randomised trial confirms evidence from previous smaller trials that treatment with baricitinib can reduce mortality in patients hospitalised with COVID-19, although the size of the benefit is about half that previously thought. The benefits appear to be consistent regardless of treatment with remdesivir, systemic corticosteroids, or an IL-6 receptor blocker such as tocilizumab. The results support the use of baricitinib in addition to other immunosuppressive therapies in patients hospitalised with COVID-19.

## Data sharing

The protocol, consent form, statistical analysis plan, definition and derivation of clinical characteristics and outcomes, training materials, regulatory documents, and other relevant study materials are available online. As described in the protocol, the trial steering committee will facilitate the use of the study data and approval will not be unreasonably withheld. De-identified participant data will be made available to bona fide researchers registered with an appropriate institution within 3 months of publication. However, the steering committee will need to be satisfied that any proposed publication is of high quality, honours the commitments made to the study participants in the consent documentation and ethical approvals, and is compliant with relevant legal and regulatory requirements (eg, relating to data protection and privacy). The steering committee will have the right to review and comment on any draft manuscripts before publication. Data will be made available in line with the policy and procedures. Those wishing to request access should complete the form and email it to data.access@ndph.ox.ac.uk.

For more on **this policy** see https://www.ndph.ox.ac.uk/files/about/ndph-independence-of-research-policy-jun-20.pdf

For **policy and procedures** see https://www.ndph.ox.ac.uk/data-access

For **the form** see https://www.ndph.ox.ac.uk/files/about/data_access_enquiry_form_13_6_2019.docx

For the **trial details** see www.recoverytrial.net

For more on the **RECOVERY trial** see www.recoverytrial.net

## Declaration of interests

The authors declare no competing interests or financial relationships relevant to the submitted work. No form of payment was given to anyone to produce the manuscript. All authors have completed and submitted the International Committee of Journal Editors form for disclosure of potential conflicts of interest. The Nuffield Department of Population Health at the University of Oxford has a staff policy of not accepting honoraria or consultancy fees directly or indirectly from industry.
